# Supplementary material for: Bacterial DNA invasion triggers transposable element proliferation and genome expansion
Source: bioRxiv. 2026 Jul 20:2026.07.14.738529. Preprint. [Version 1] doi: 10.64898/2026.07.14.738529 (PMC13419823; doi:10.64898/2026.07.14.738529)
Supplement: Supplementary file 1 [file media-1.pdf]

Supplementary material for *Bacterial DNA invasion triggers transposable element proliferation and genome expansion*

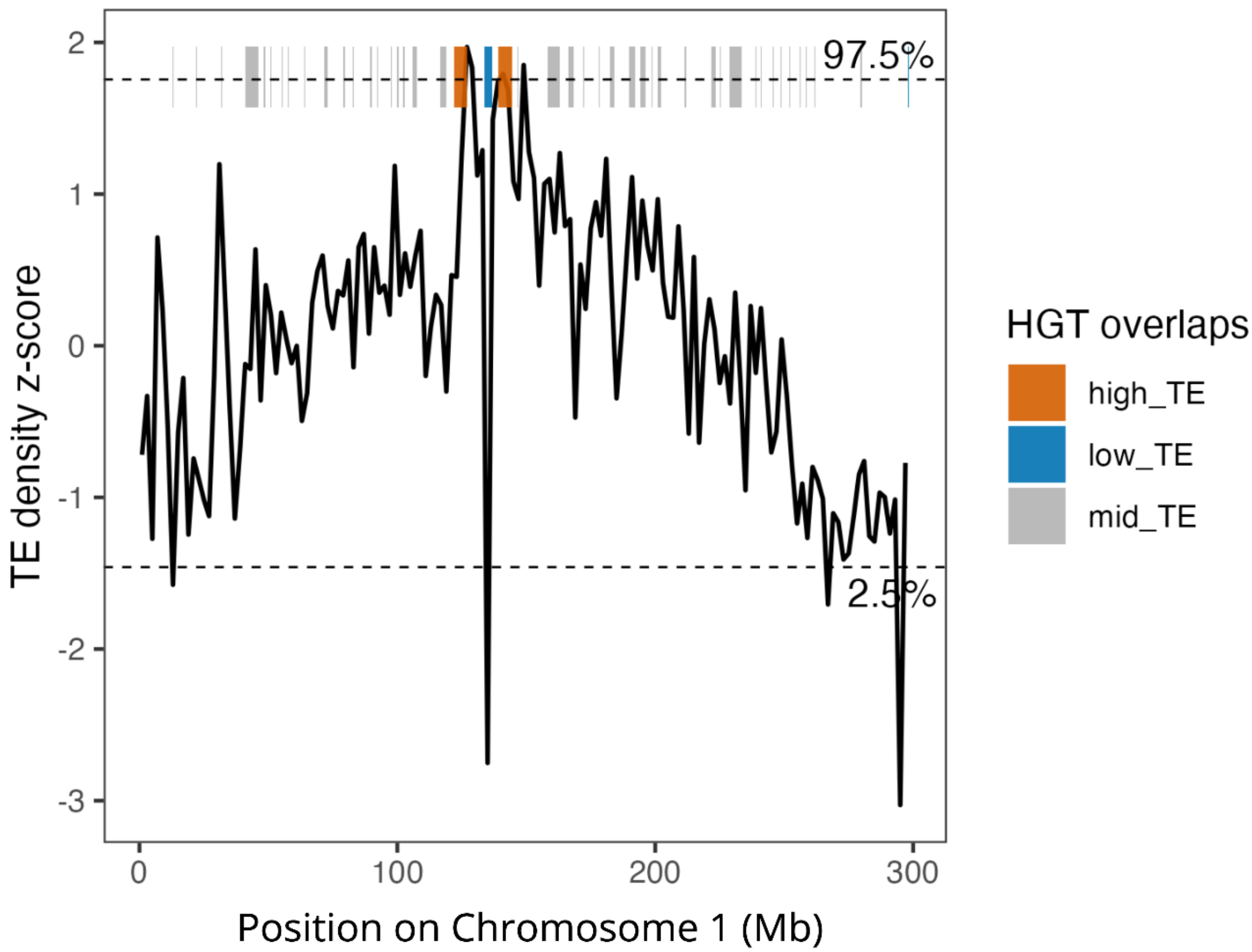

Supplementary Figure 1. Z-score transformed TE density in sliding 2 Mb windows across Chromosome 1 of *C. caryae*. HGTs located within the upper (97.5th percentile) and lower (2.5th percentile) tails of the TE density distribution are indicated by orange and blue bars, respectively. Gray bars indicate HGTs falling outside these tails.

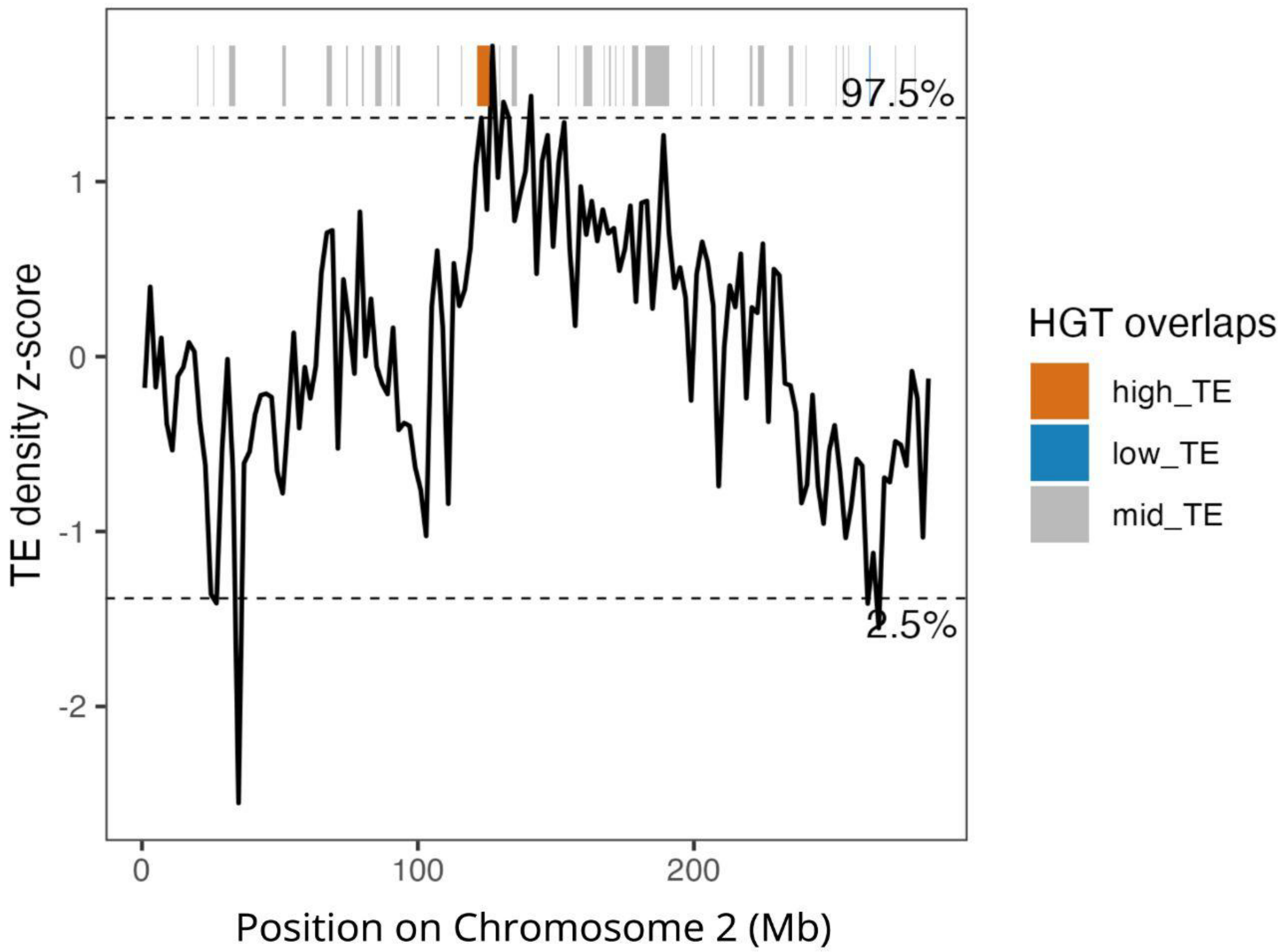

Supplementary Figure 2. Z-score transformed TE density by type in sliding 2 Mb windows across Chromosome 2 of *C. caryae*. HGTs located within the upper (97.5th percentile) and lower (2.5th percentile) tails of the TE density distribution are indicated by orange and blue bars, respectively. Gray bars indicate HGTs falling outside these tails.

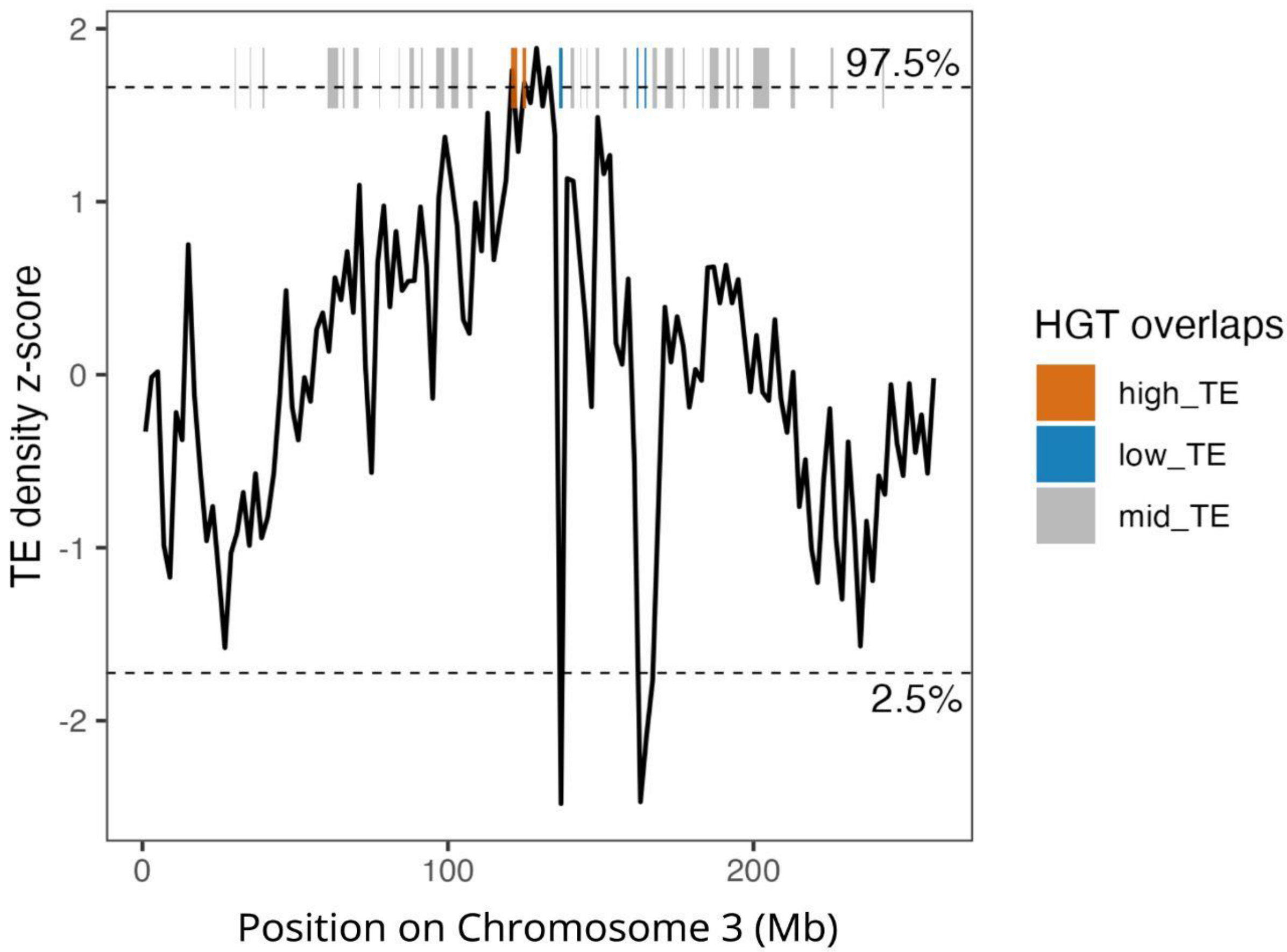

Supplementary Figure 3. Z-score transformed TE density by type in sliding 2 Mb windows across Chromosome 3 of *C. caryae*. HGTs located within the upper (97.5th percentile) and lower (2.5th percentile) tails of the TE density distribution are indicated by orange and blue bars, respectively. Gray bars indicate HGTs falling outside these tails.

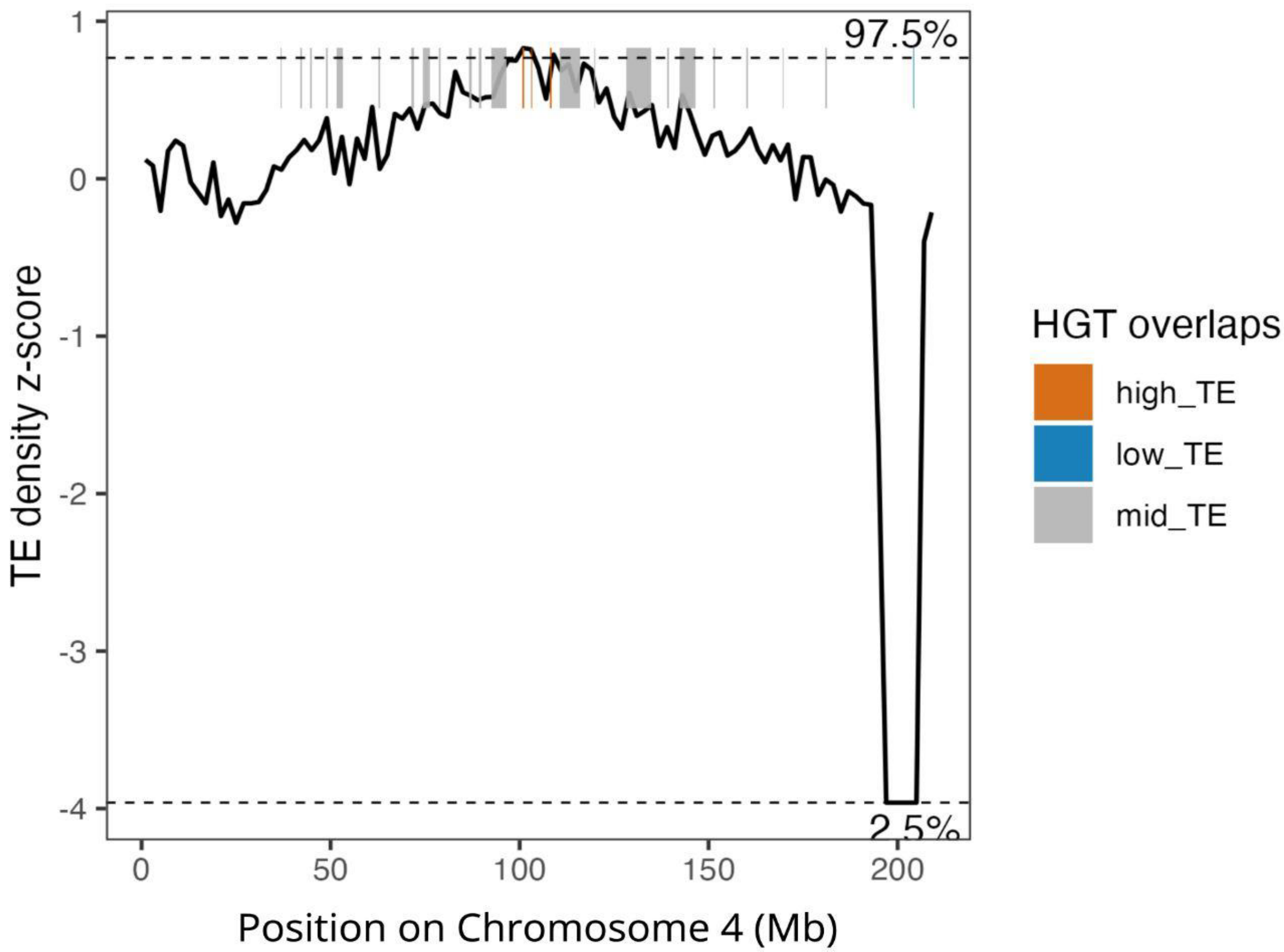

Supplementary Figure 4. Z-score transformed TE density by type in sliding 2 Mb windows across Chromosome 4 of *C. caryae*. HGTs located within the upper (97.5th percentile) and lower (2.5th percentile) tails of the TE density distribution are indicated by orange and blue bars, respectively. Gray bars indicate HGTs falling outside these tails.

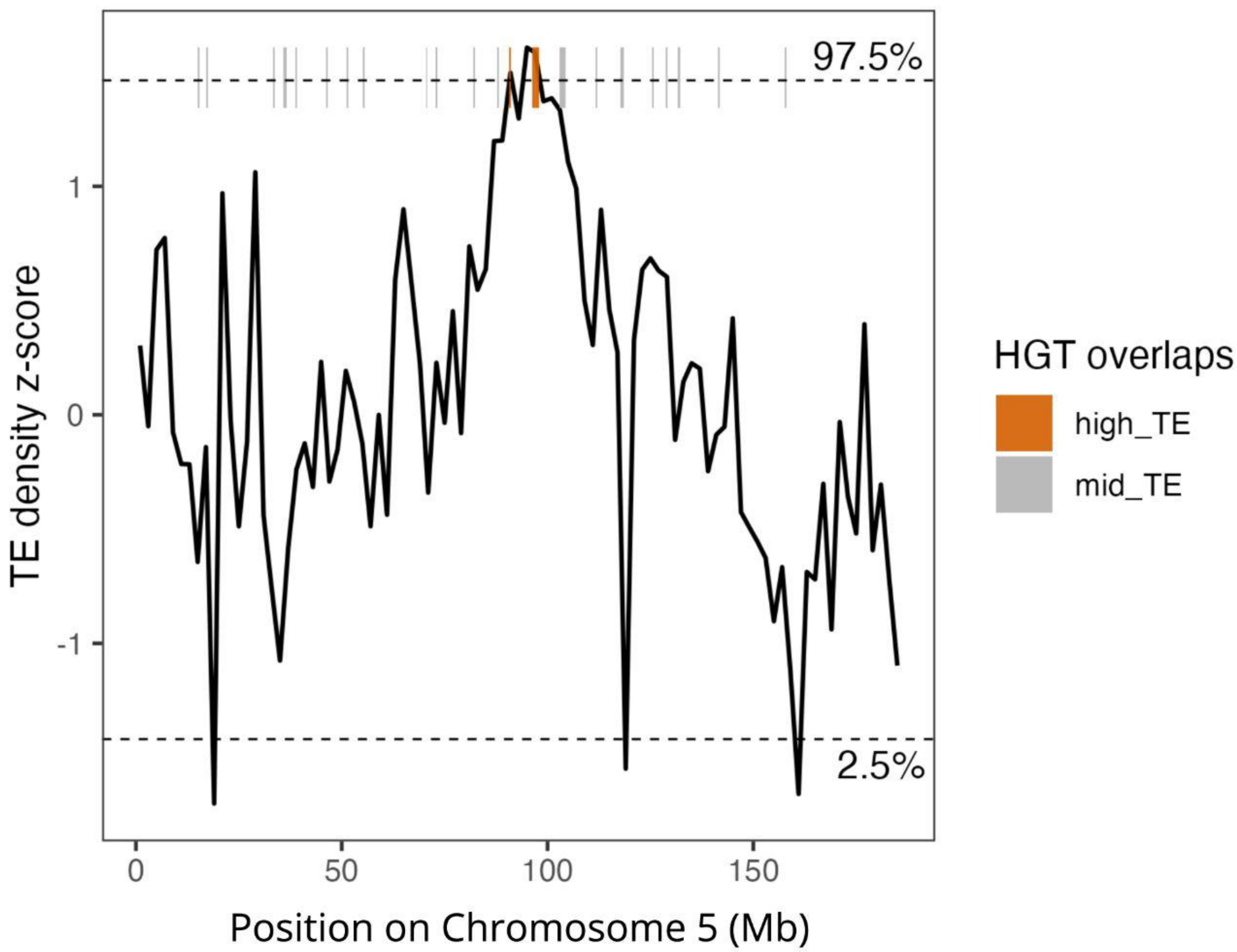

Supplementary Figure 5. Z-score transformed TE density by type in sliding 2 Mb windows across Chromosome 5 of *C. caryae*. HGTs located within the upper (97.5th percentile) tail of the TE density distribution are indicated by orange bars. Gray bars indicate HGTs falling outside this tail.

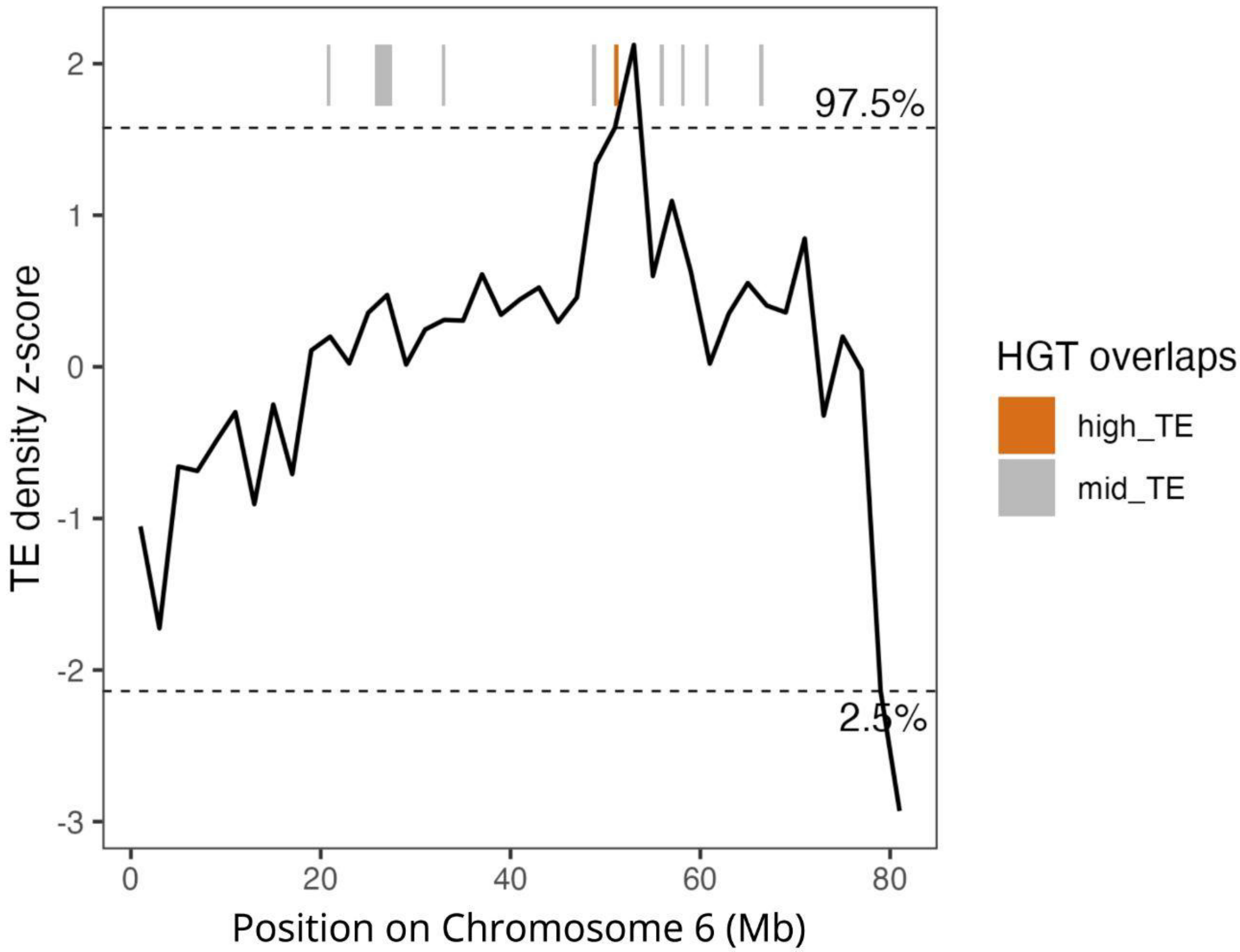

Supplementary Figure 6. Z-score transformed TE density by type in sliding 2 Mb windows across Chromosome 6 of *C. caryae*. HGTs located within the upper (97.5th percentile) and lower (2.5th percentile) tails of the TE density distribution are indicated by orange and blue bars, respectively. Gray bars indicate HGTs falling outside these tails.

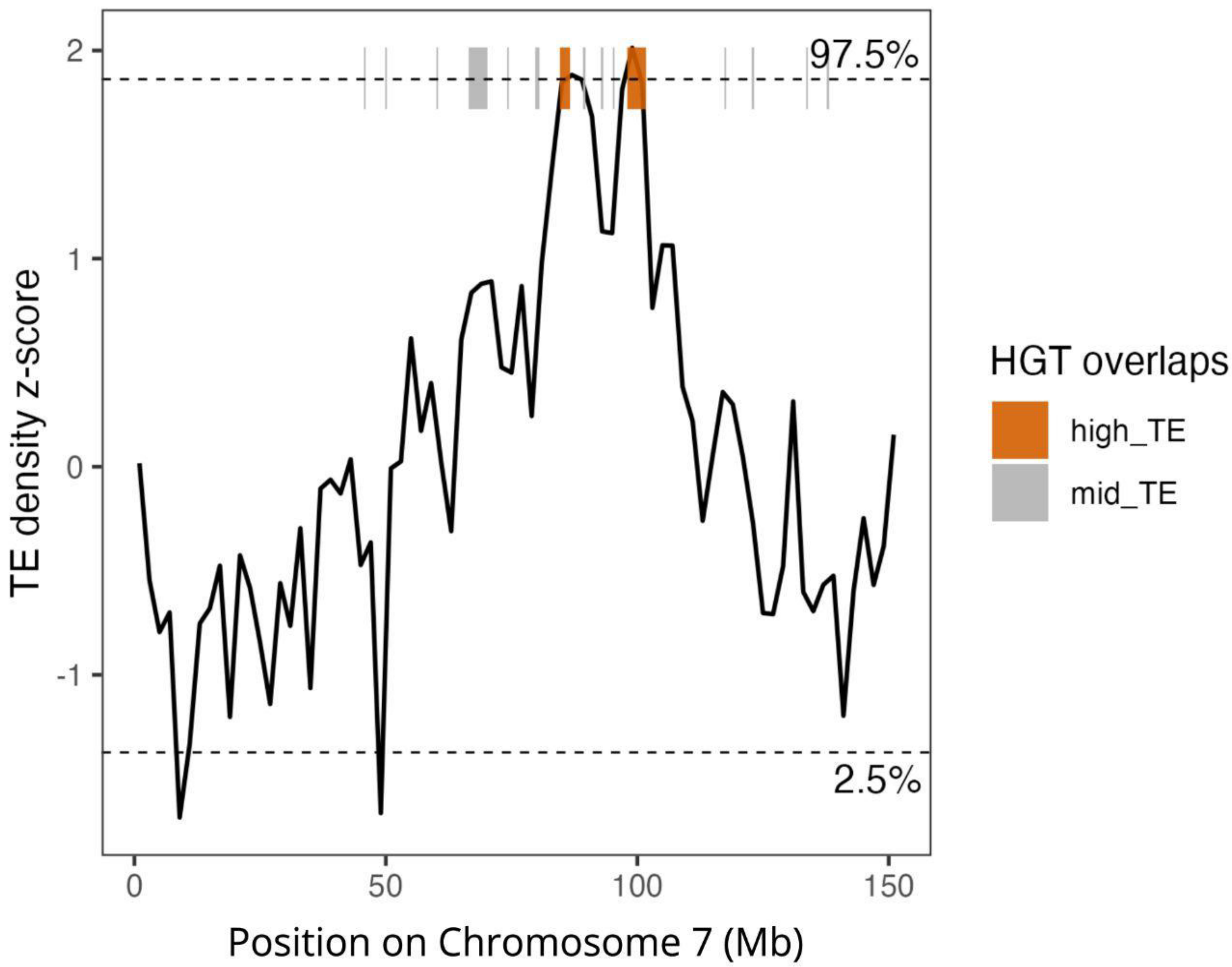

Supplementary Figure 7. Z-score transformed TE density by type in sliding 2 Mb windows across Chromosome 7 of *C. caryae*. HGTs located within the upper (97.5th percentile) tail of the TE density distribution are indicated by orange bars. Gray bars indicate HGTs falling outside this tail.

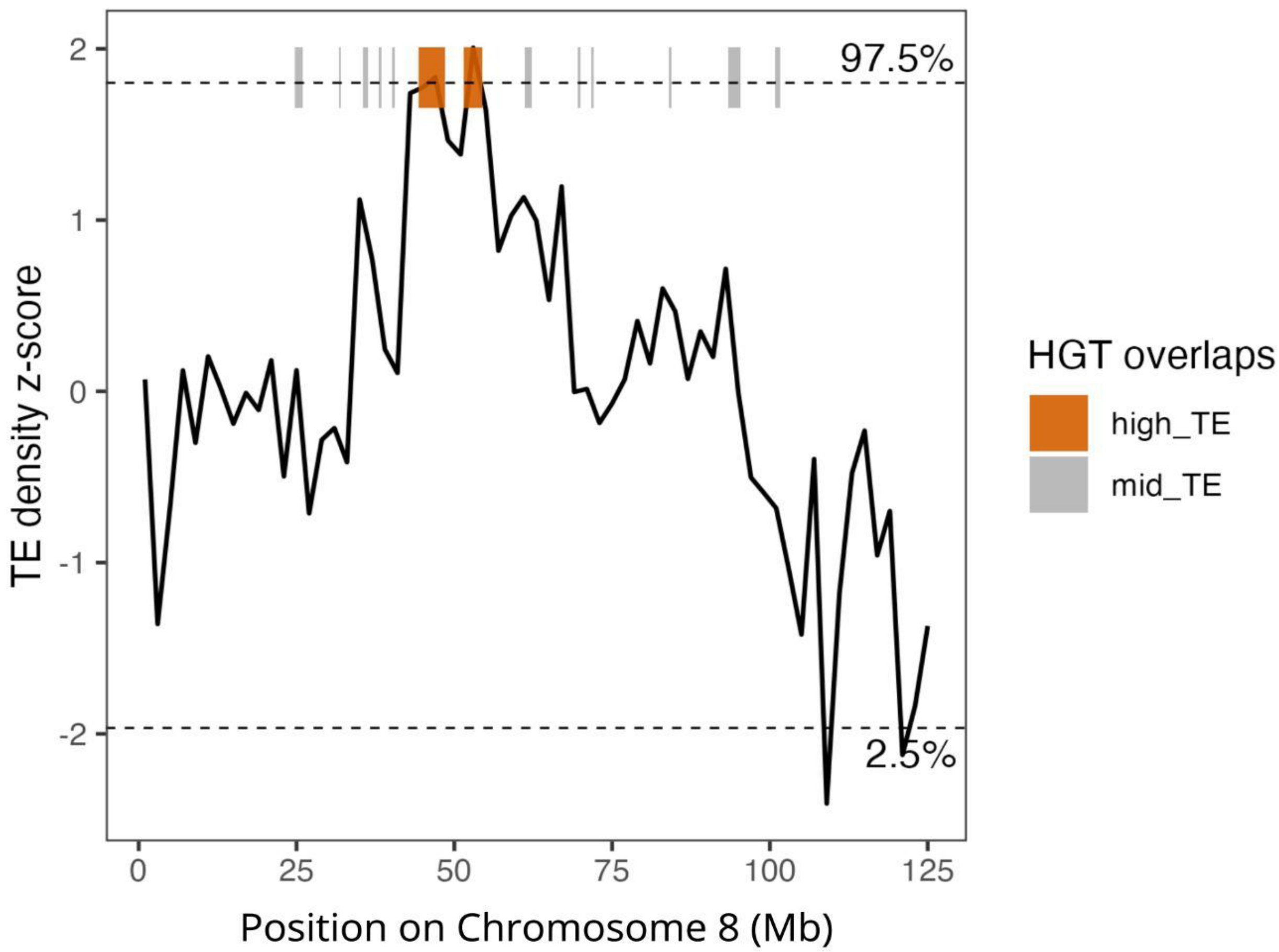

Supplementary Figure 8. Z-score transformed TE density by type in sliding 2 Mb windows across Chromosome 8 of *C. caryae*. HGTs located within the upper (97.5th percentile) tail of the TE density distribution are indicated by orange bars. Gray bars indicate HGTs falling outside this tail.

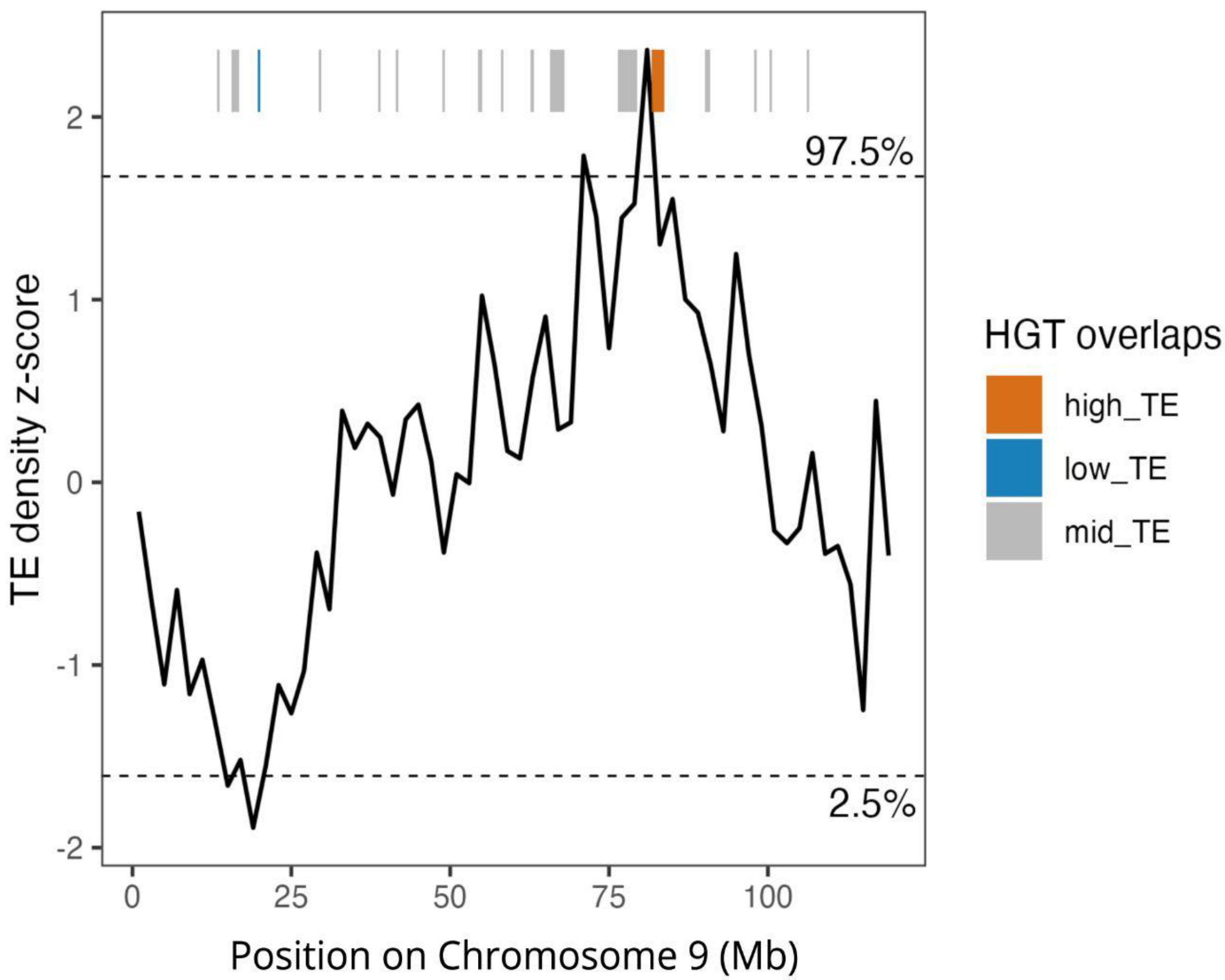

Supplementary Figure 9. Z-score transformed TE density by type in sliding 2 Mb windows across Chromosome 9 of *C. caryae*. HGTs located within the upper (97.5th percentile) and lower (2.5th percentile) tails of the TE density distribution are indicated by orange and blue bars, respectively. Gray bars indicate HGTs falling outside these tails.

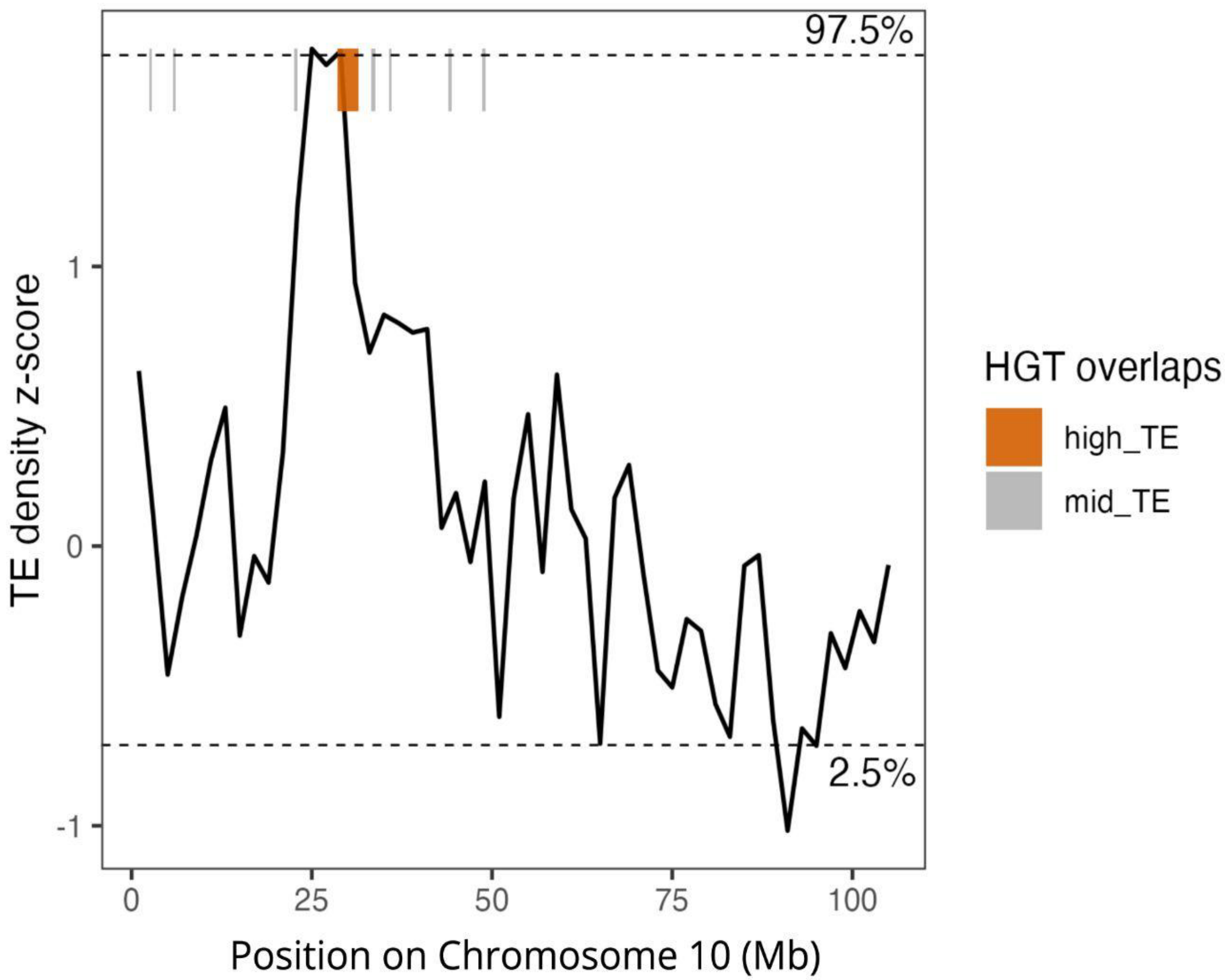

Supplementary Figure 10. Z-score transformed TE density by type in sliding 2 Mb windows across Chromosome 10 of *C. caryae*. HGTs located within the upper (97.5th percentile) tail of the TE density distribution are indicated by orange bars. Gray bars indicate HGTs falling outside this tail.

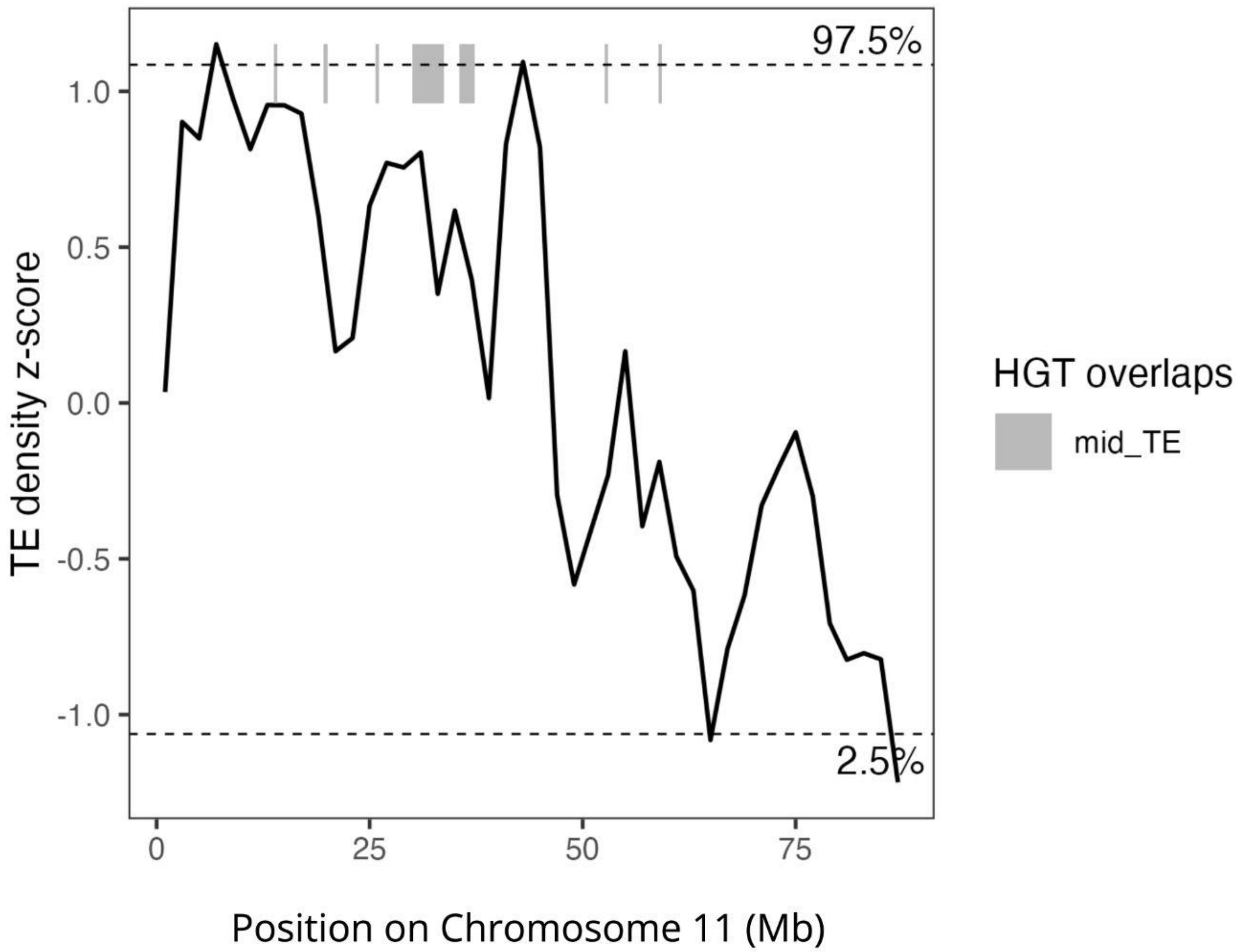

Supplementary Figure 11. Z-score transformed TE density by type in sliding 2 Mb windows across Chromosome 11 of *C. caryae*. Horizontally transferred DNA sequences (HGTs) located outside of the tails of the TE density distribution are indicated with gray bars.

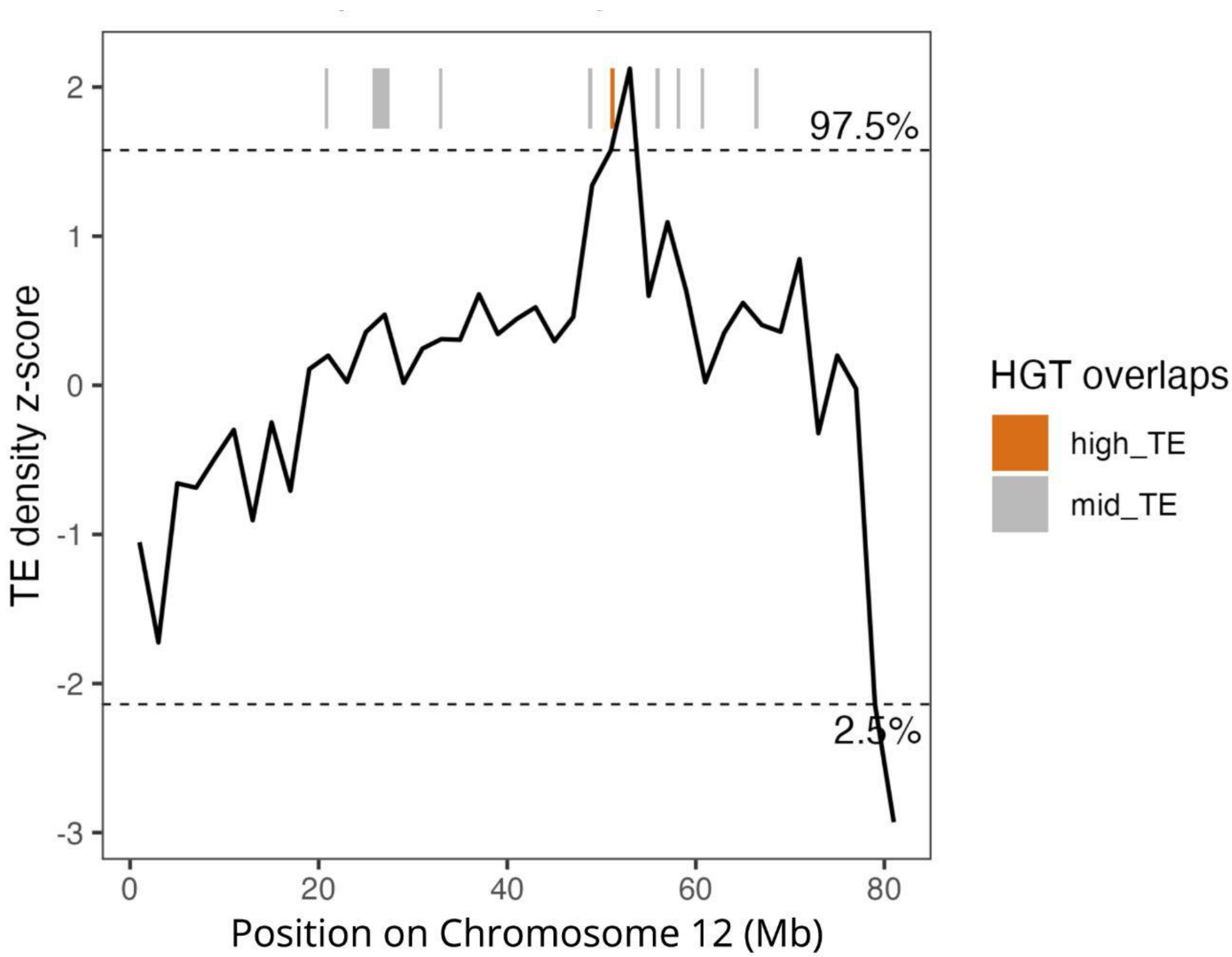

Supplementary Figure 12. Z-score transformed TE density by type in sliding 2 Mb windows across Chromosome 12 of *C. caryae*. HGTs located within the upper (97.5th percentile) tail of the TE density distribution are indicated by orange bars. Gray bars indicate HGTs falling outside this tail.

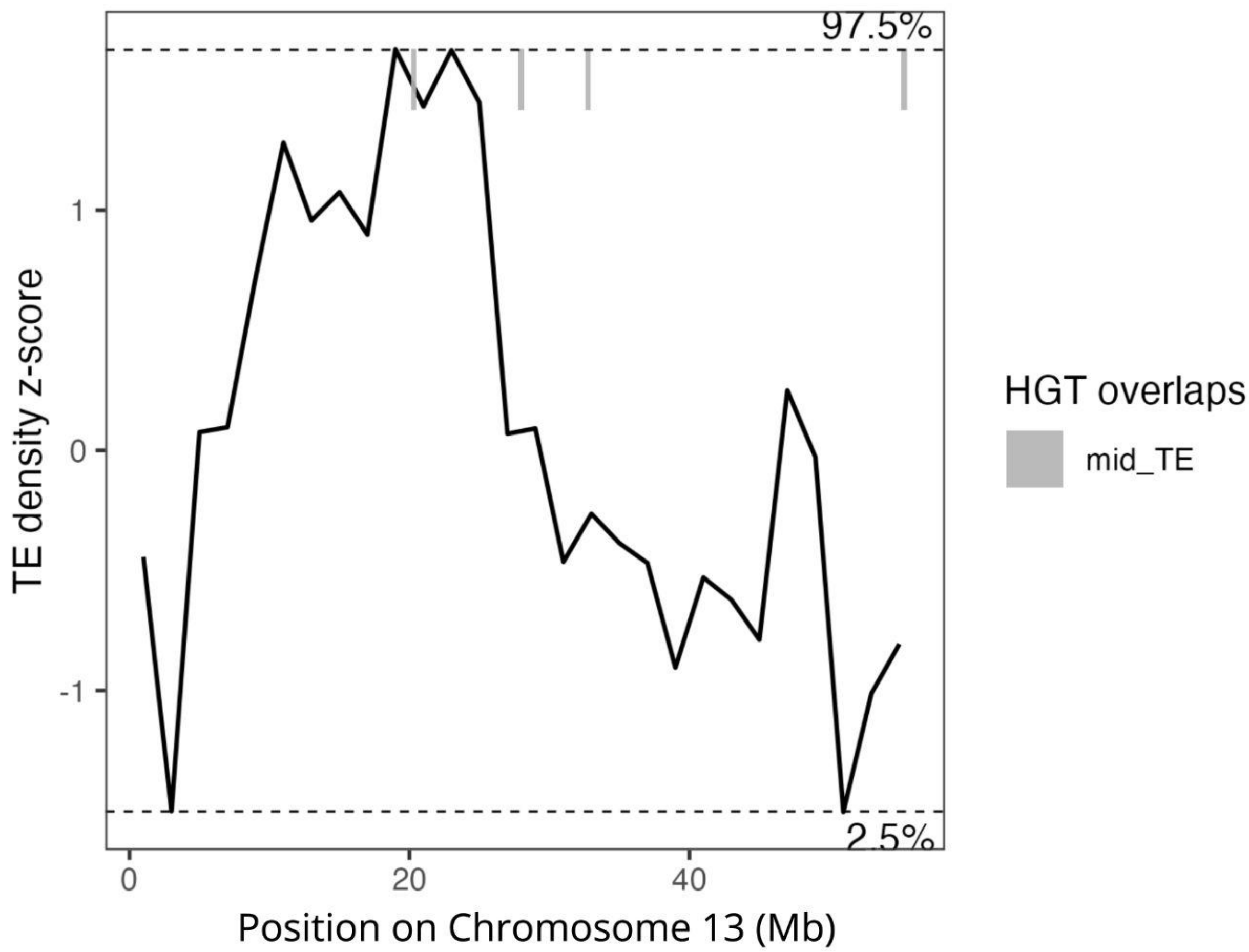

Supplementary Figure 13. Z-score transformed TE density by type in sliding 2 Mb windows across Chromosome 13 of *C. caryae*. Horizontally transferred DNA sequences (HGTs) located outside of the tails of the TE density distribution are indicated with gray bars.

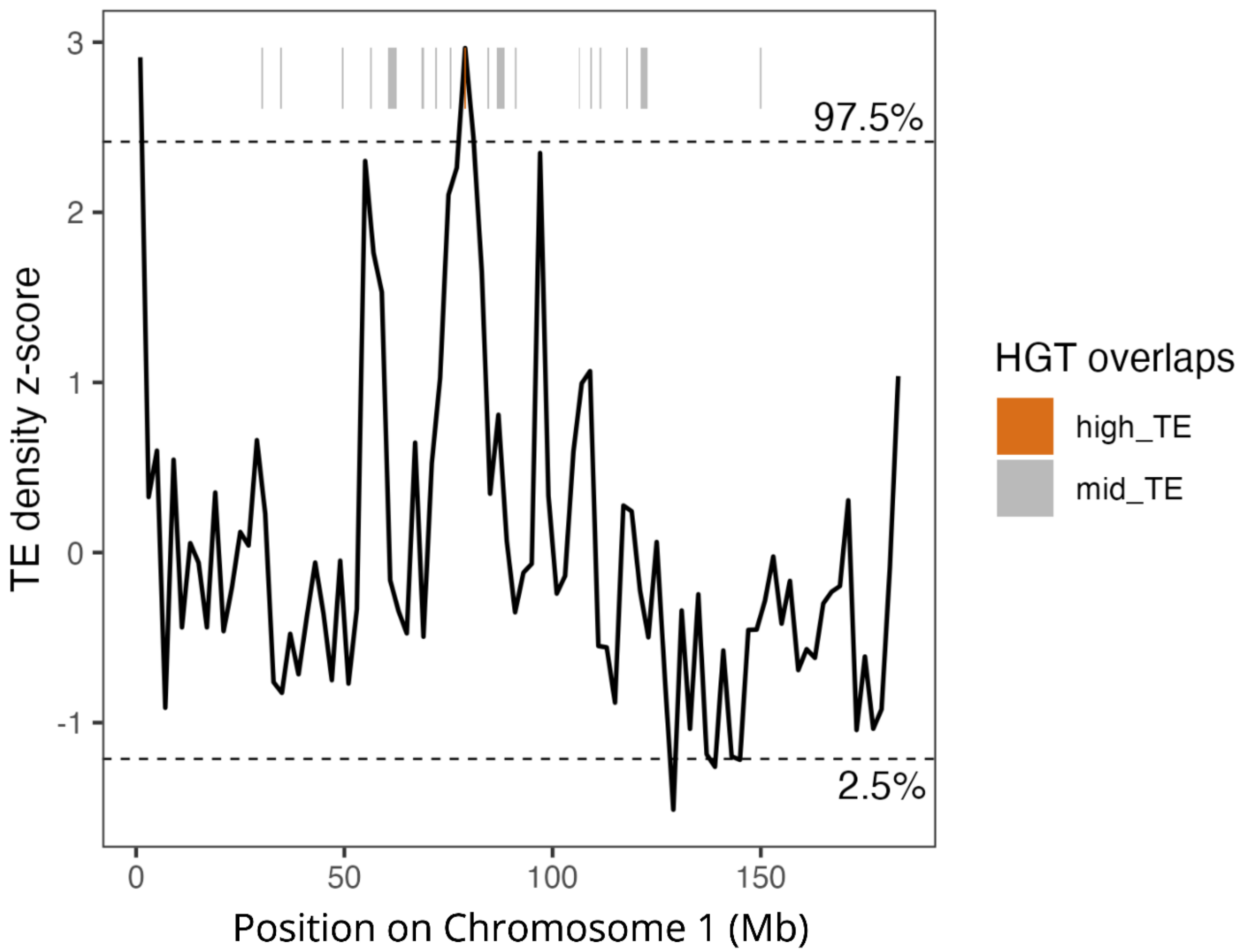

Supplementary Figure 14. Z-score transformed TE density by type in sliding 2 Mb windows across Chromosome 1 of *C. nanulus*. HGTs located within the upper (97.5th percentile) tail of the TE density distribution are indicated by orange bars. Gray bars indicate HGTs falling outside this tail.

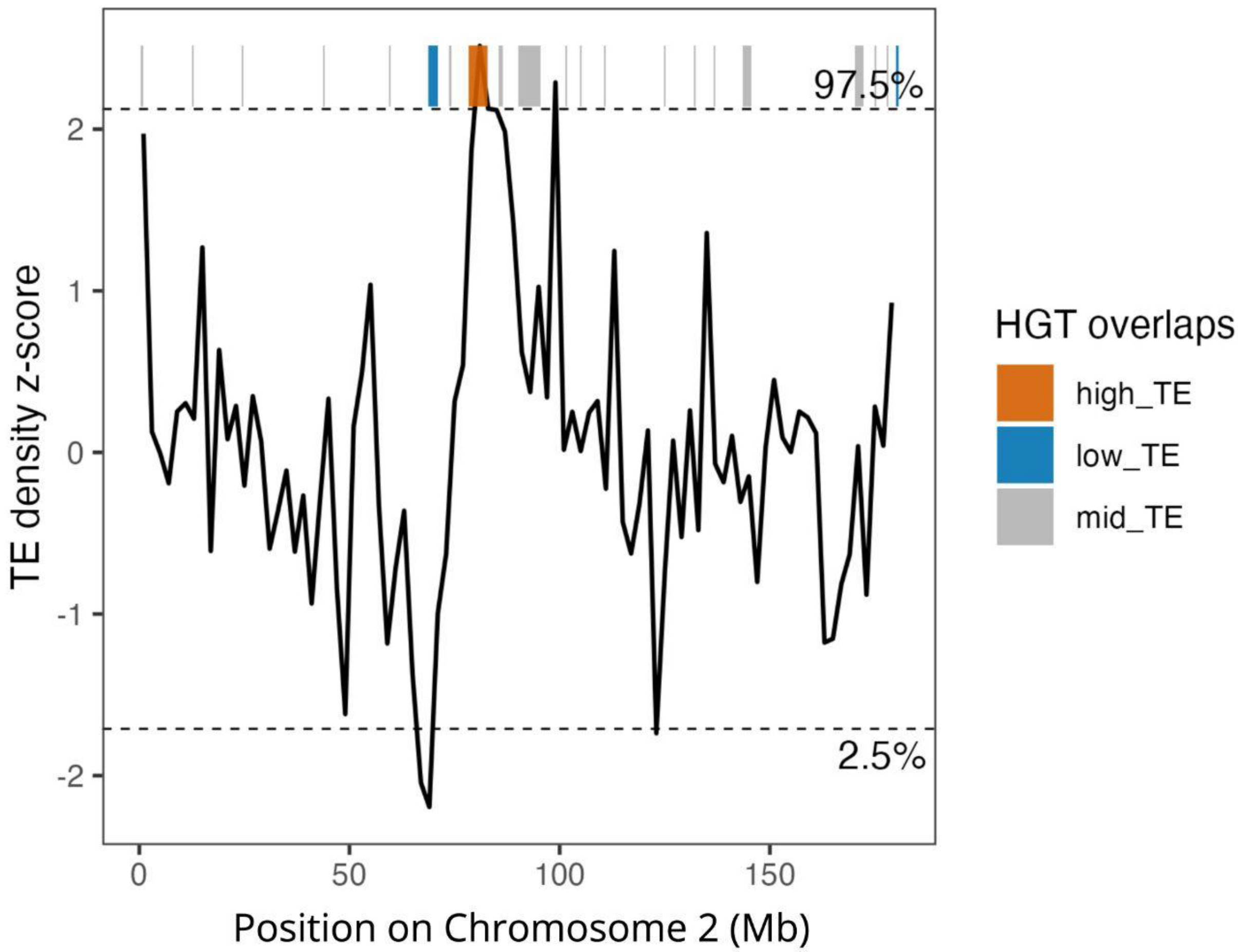

Supplementary Figure 15. Z-score transformed TE density by type in sliding 2 Mb windows across Chromosome 2 of *C. nanulus*. HGTs located within the upper (97.5th percentile) and lower (2.5th percentile) tails of the TE density distribution are indicated by orange and blue bars, respectively. Gray bars indicate HGTs falling outside these tails.

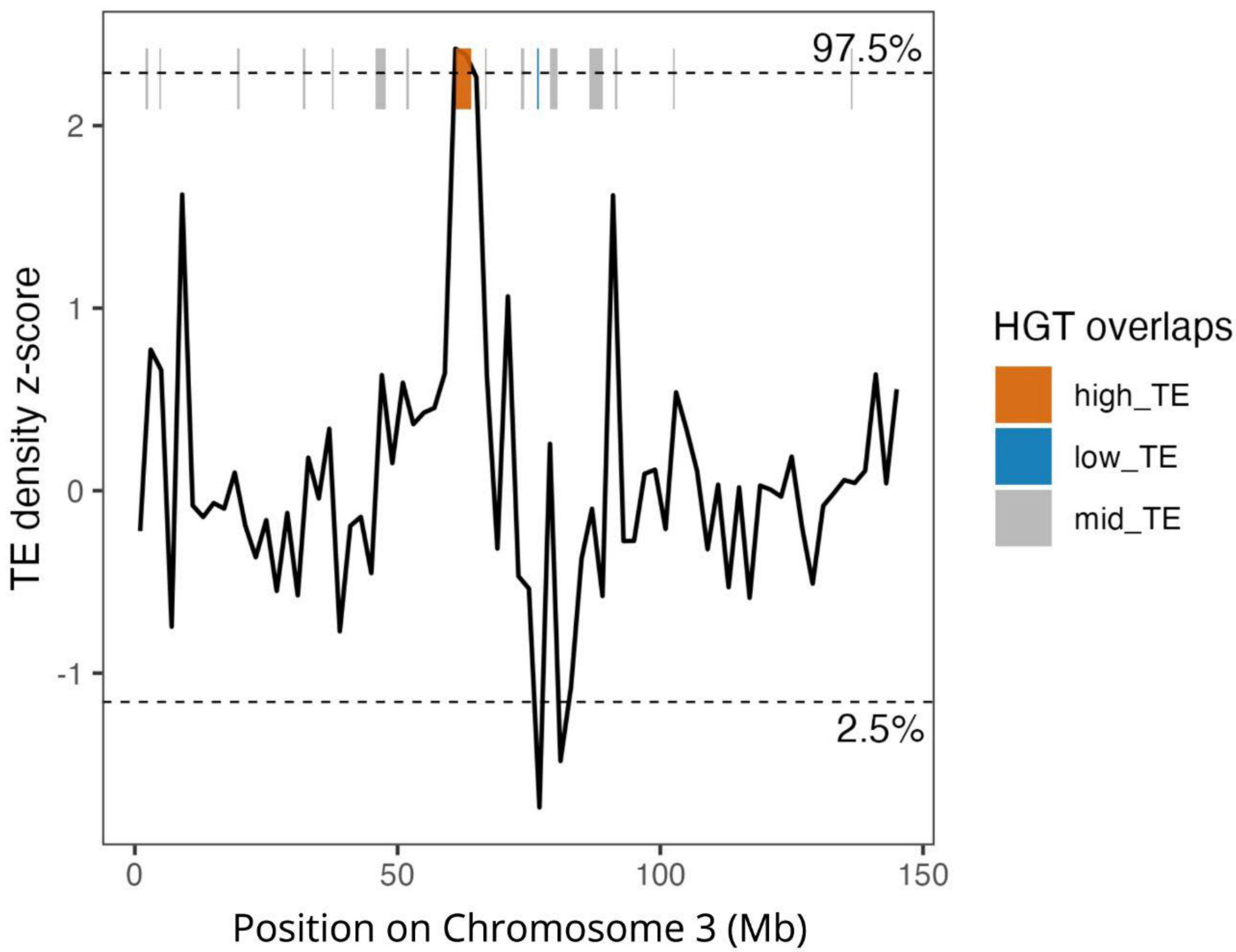

Supplementary Figure 16. Z-score transformed TE density by type in sliding 2 Mb windows across Chromosome 3 of *C. nanulus*. HGTs located within the upper (97.5th percentile) and lower (2.5th percentile) tails of the TE density distribution are indicated by orange and blue bars, respectively. Gray bars indicate HGTs falling outside these tails.

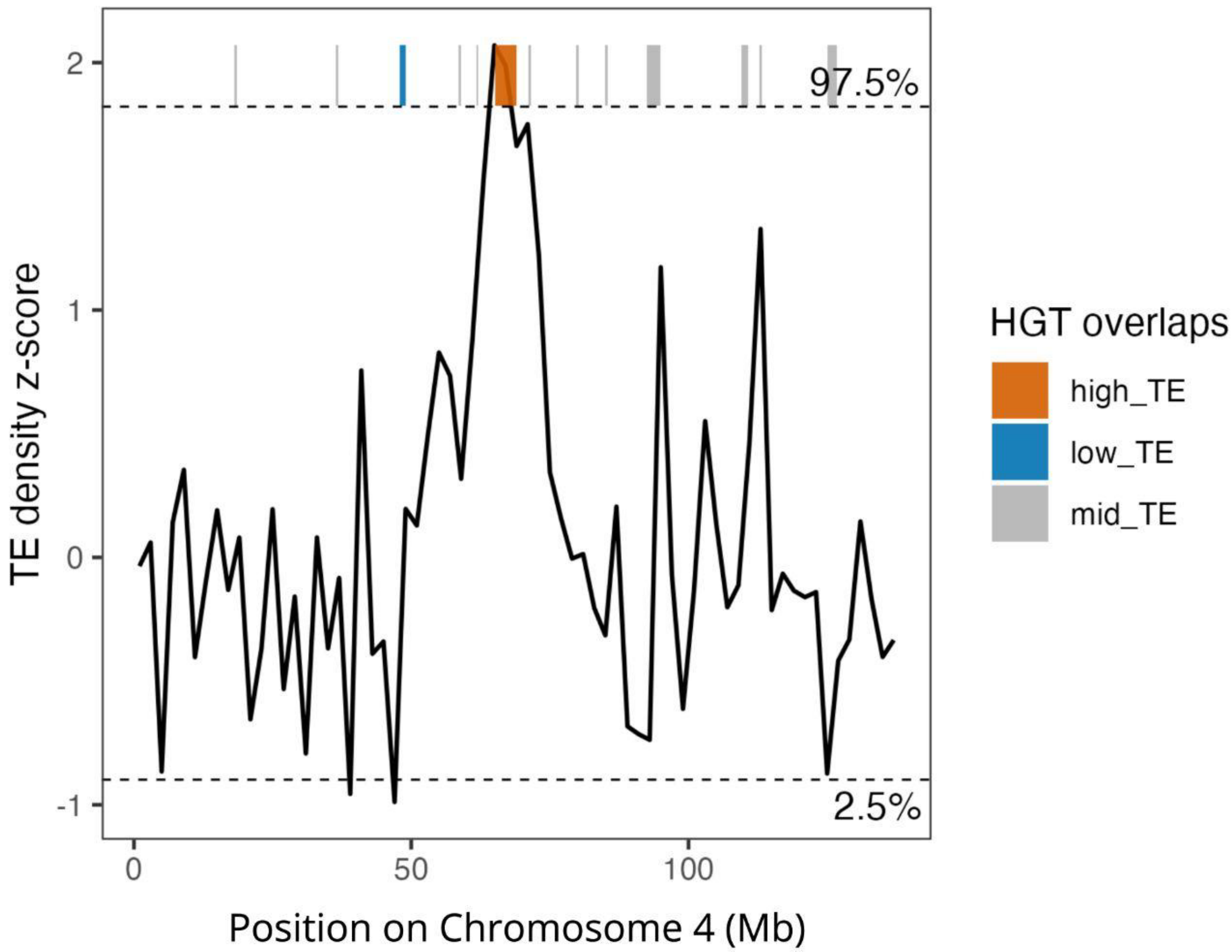

Supplementary Figure 17. Z-score transformed TE density by type in sliding 2 Mb windows across Chromosome 4 of *C. nanulus*. HGTs located within the upper (97.5th percentile) and lower (2.5th percentile) tails of the TE density distribution are indicated by orange and blue bars, respectively. Gray bars indicate HGTs falling outside these tails.

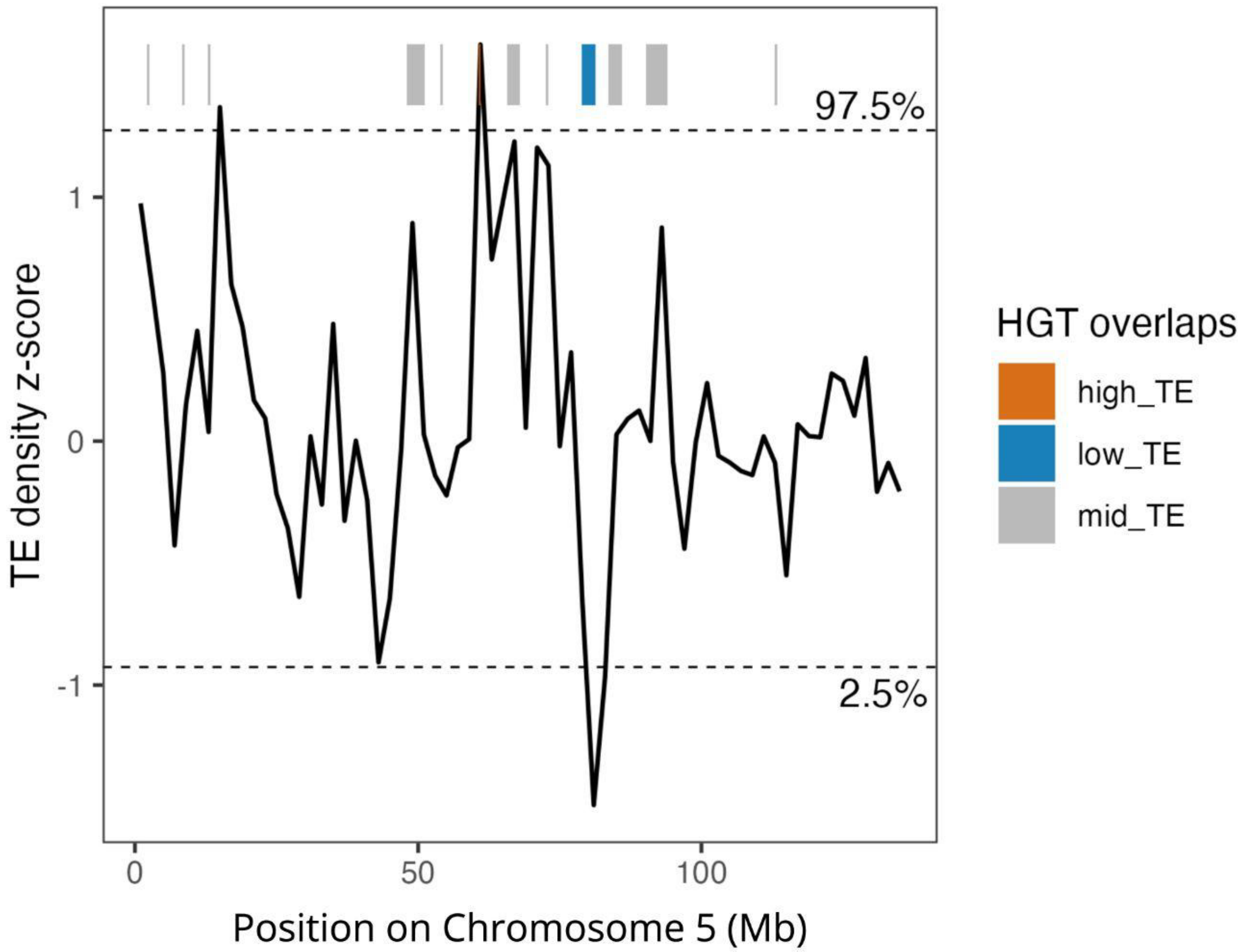

Supplementary Figure 18. Z-score transformed TE density by type in sliding 2 Mb windows across Chromosome 5 of *C. nanulus*. HGTs located within the upper (97.5th percentile) and lower (2.5th percentile) tails of the TE density distribution are indicated by orange and blue bars, respectively. Gray bars indicate HGTs falling outside these tails.

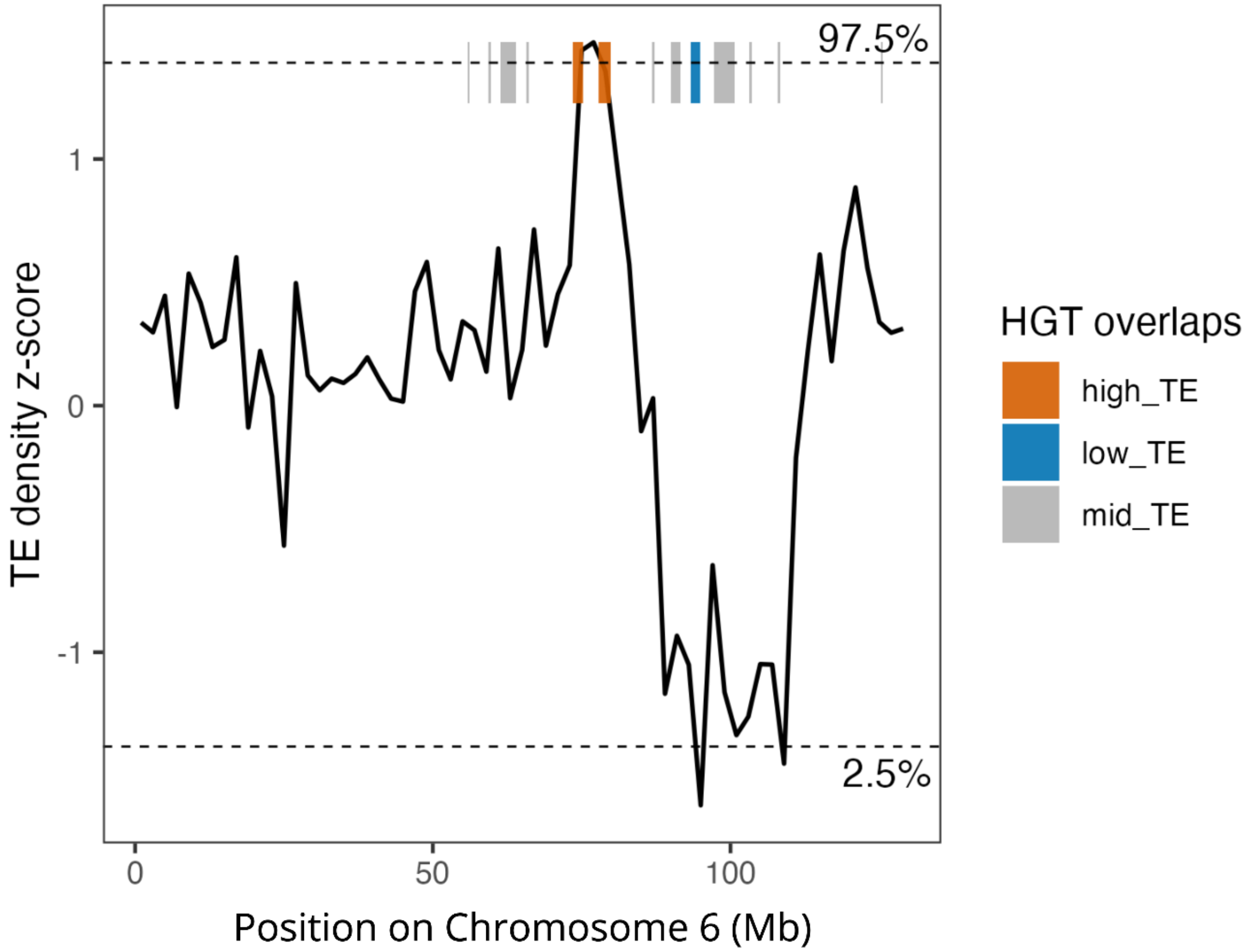

Supplementary Figure 19. Z-score transformed TE density by type in sliding 2 Mb windows across Chromosome 6 of *C. nanulus*. HGTs located within the upper (97.5th percentile) and lower (2.5th percentile) tails of the TE density distribution are indicated by orange and blue bars, respectively. Gray bars indicate HGTs falling outside these tails.

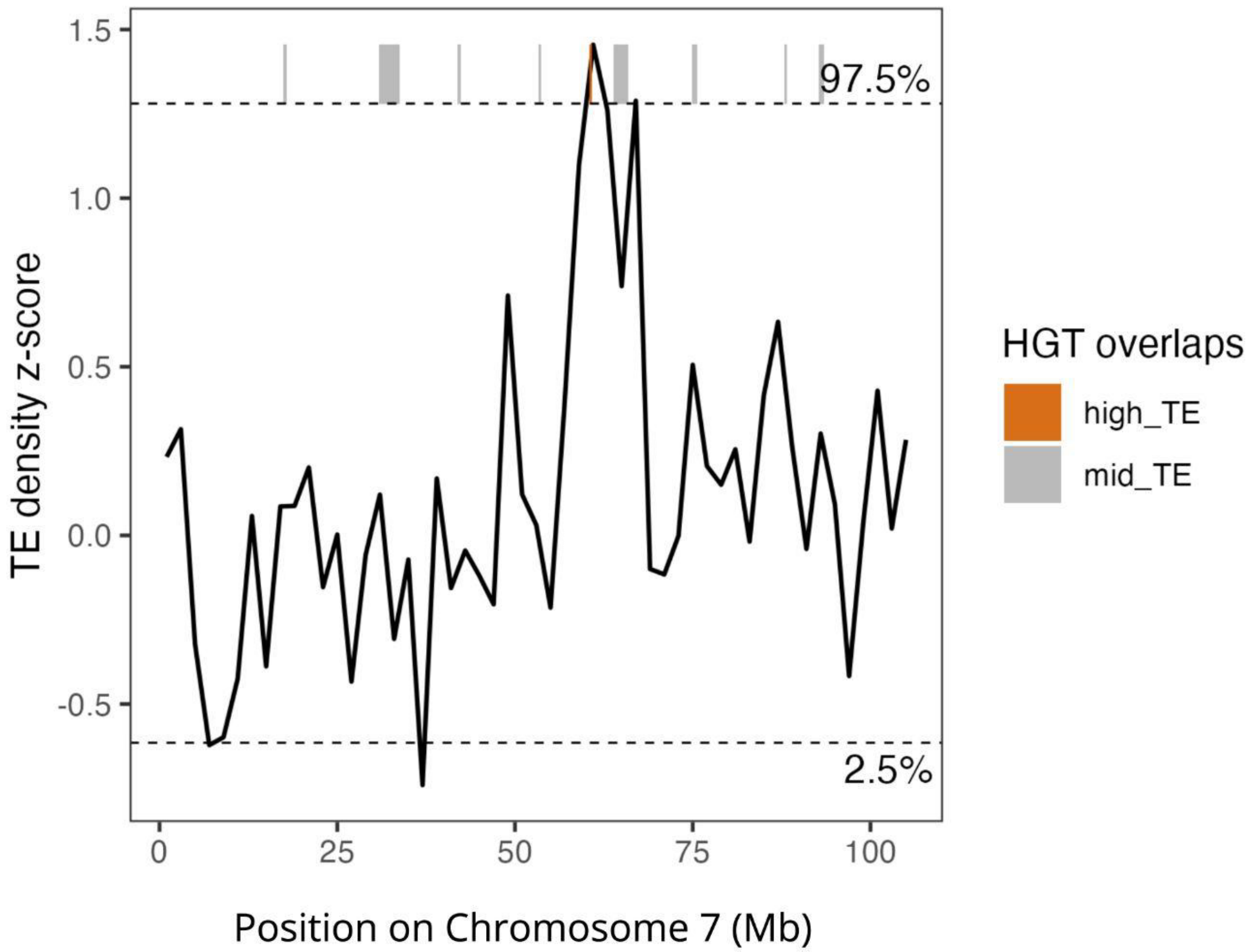

Supplementary Figure 20. Z-score transformed TE density by type in sliding 2 Mb windows across Chromosome 7 of *C. nanulus*. HGTs located within the upper (97.5th percentile) tail of the TE density distribution are indicated by orange bars. Gray bars indicate HGTs falling outside this tail.

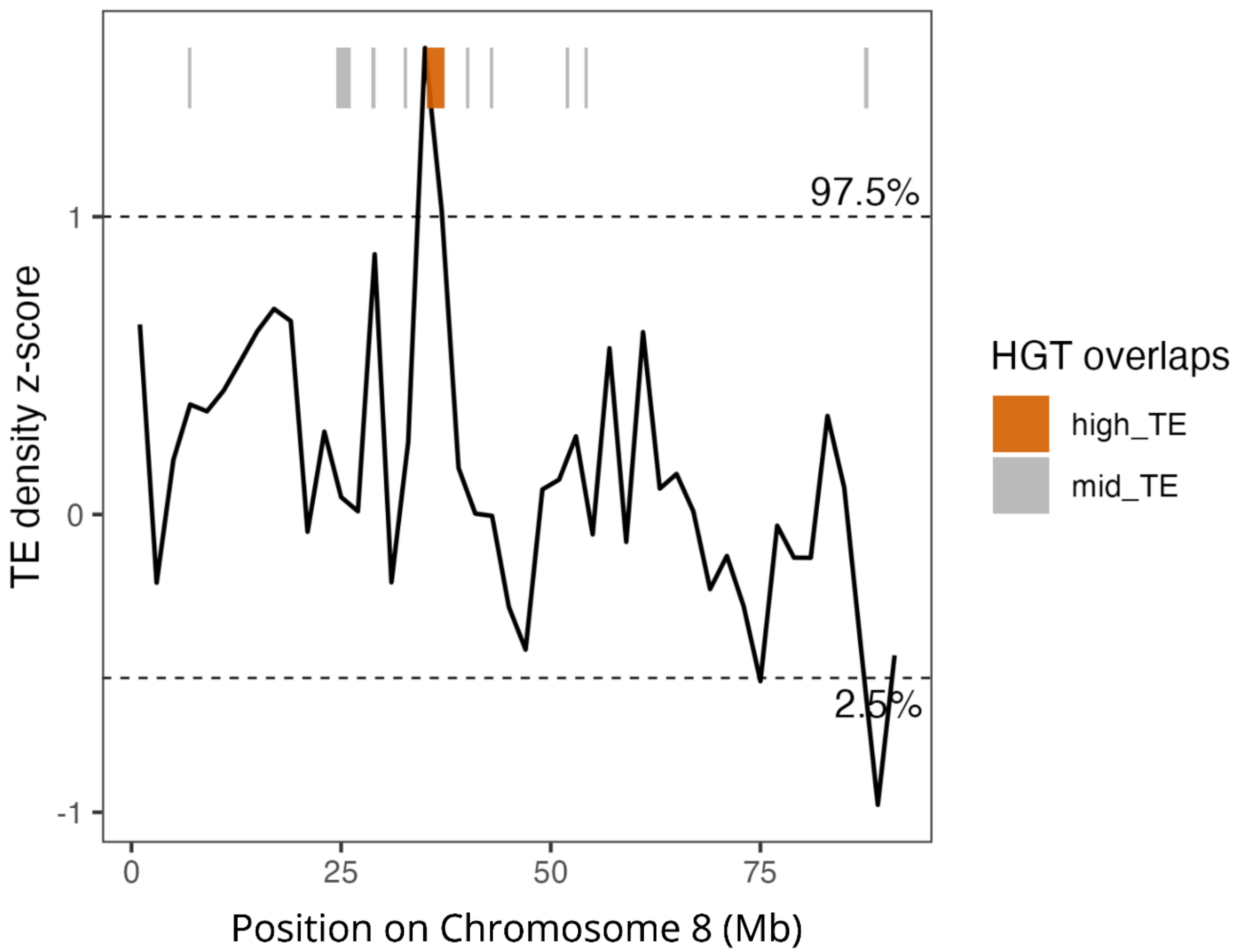

Supplementary Figure 21. Z-score transformed TE density by type in sliding 2 Mb windows across Chromosome 8 of *C. nanulus*. HGTs located within the upper (97.5th percentile) tail of the TE density distribution are indicated by orange bars. Gray bars indicate HGTs falling outside this tail.

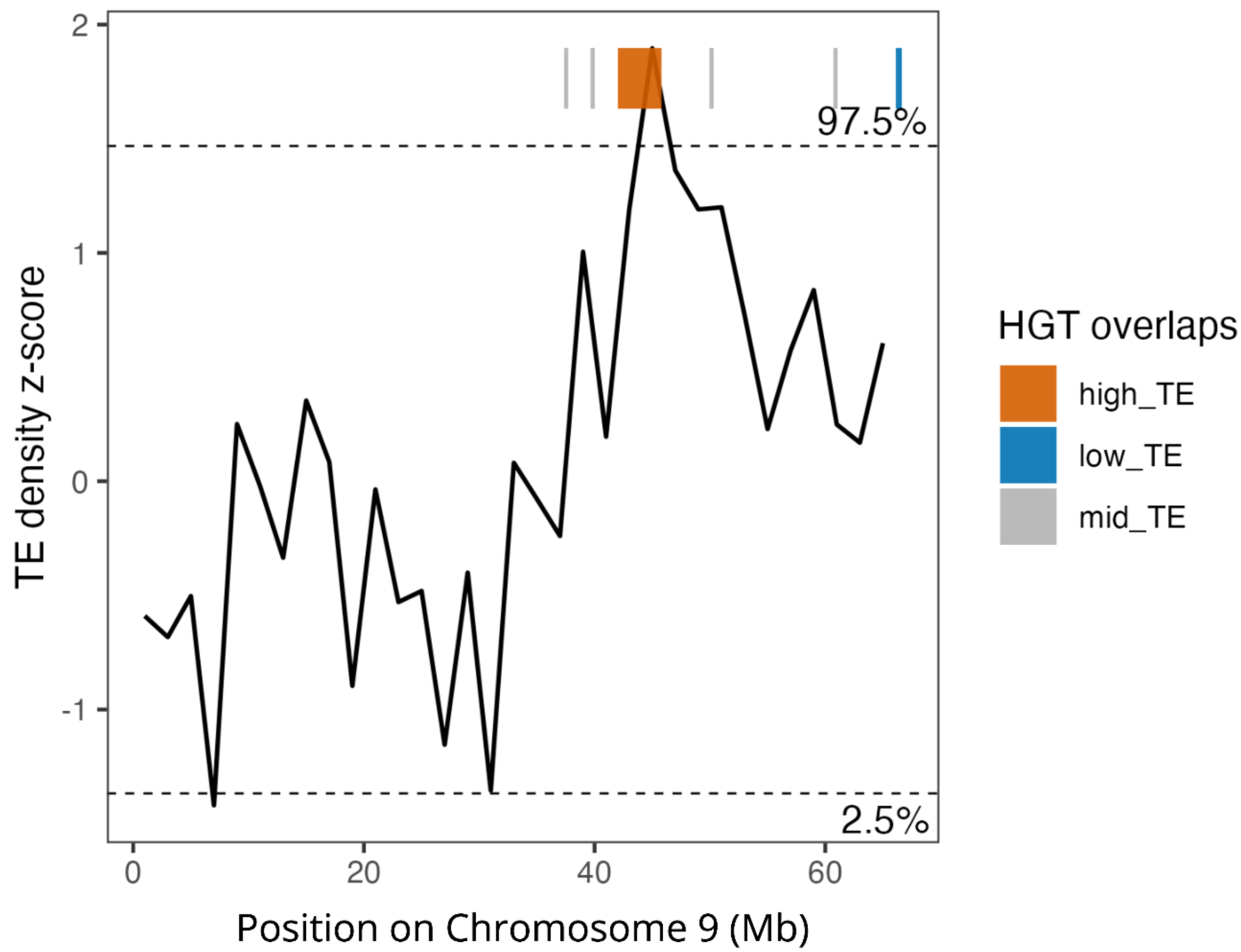

Supplementary Figure 22. Z-score transformed TE density by type in sliding 2 Mb windows across Chromosome 9 of *C. nanulus*. HGTs located within the upper (97.5th percentile) and lower (2.5th percentile) tails of the TE density distribution are indicated by orange and blue bars, respectively. Gray bars indicate HGTs falling outside these tails.

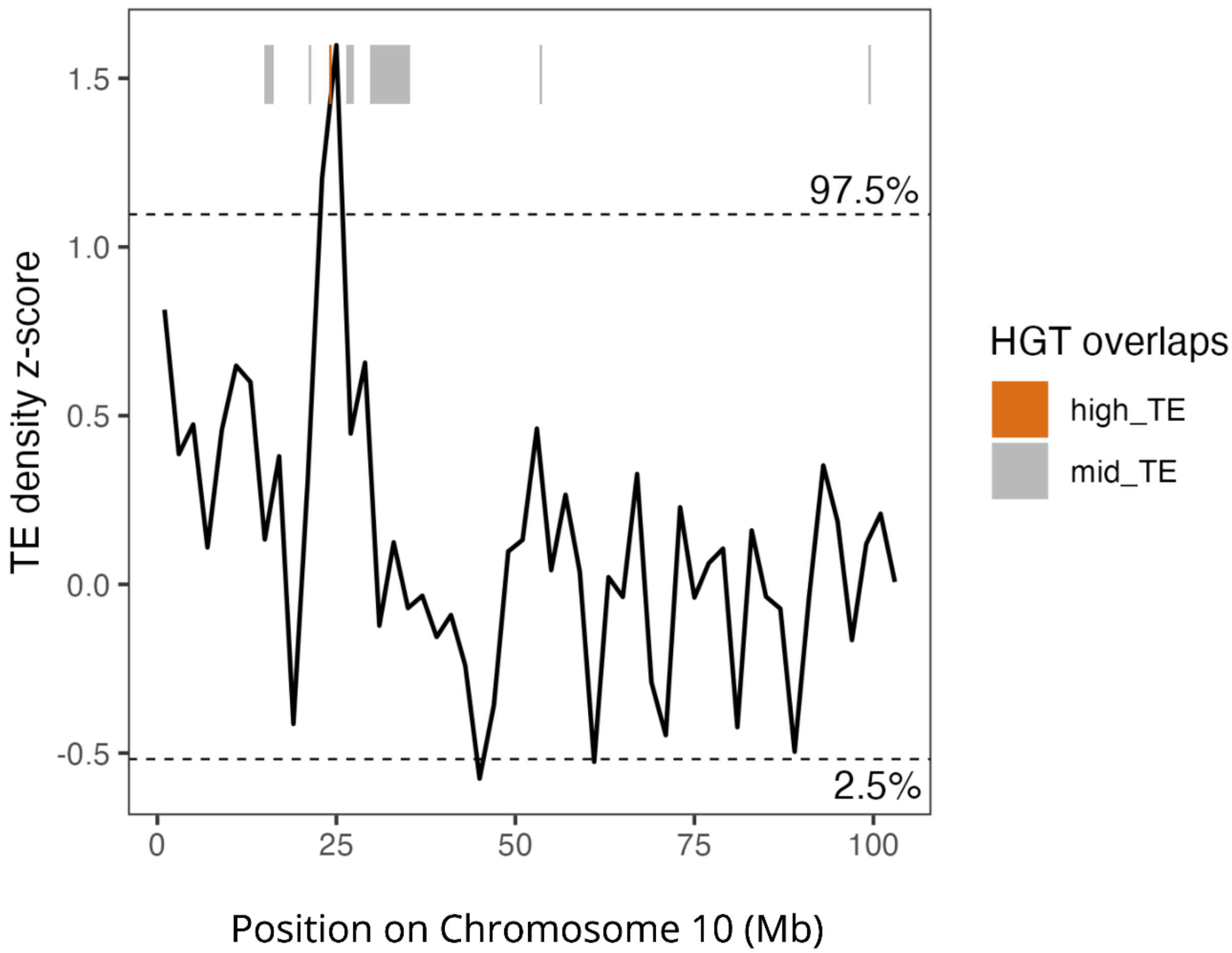

Supplementary Figure 23. Z-score transformed TE density by type in sliding 2 Mb windows across Chromosome 10 of *C. nanulus*. HGTs located within the upper (97.5th percentile) tail of the TE density distribution are indicated by orange bars. Gray bars indicate HGTs falling outside this tail.

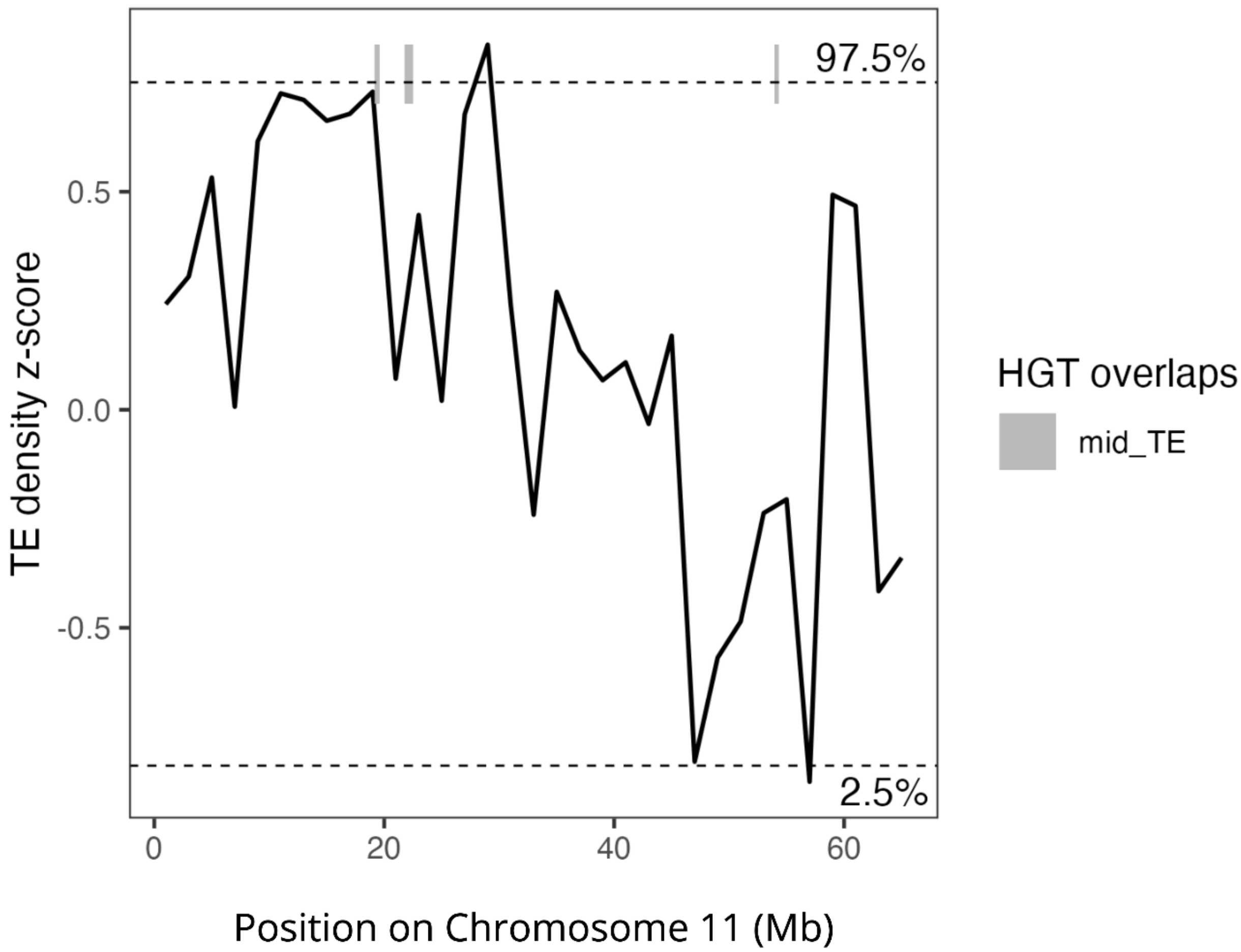

Supplementary Figure 24. Z-score transformed TE density by type in sliding 2 Mb windows across Chromosome 11 of *C. nanulus*. Horizontally transferred DNA sequences (HGTs) located outside of the tails of the TE density distribution are indicated with gray bars.

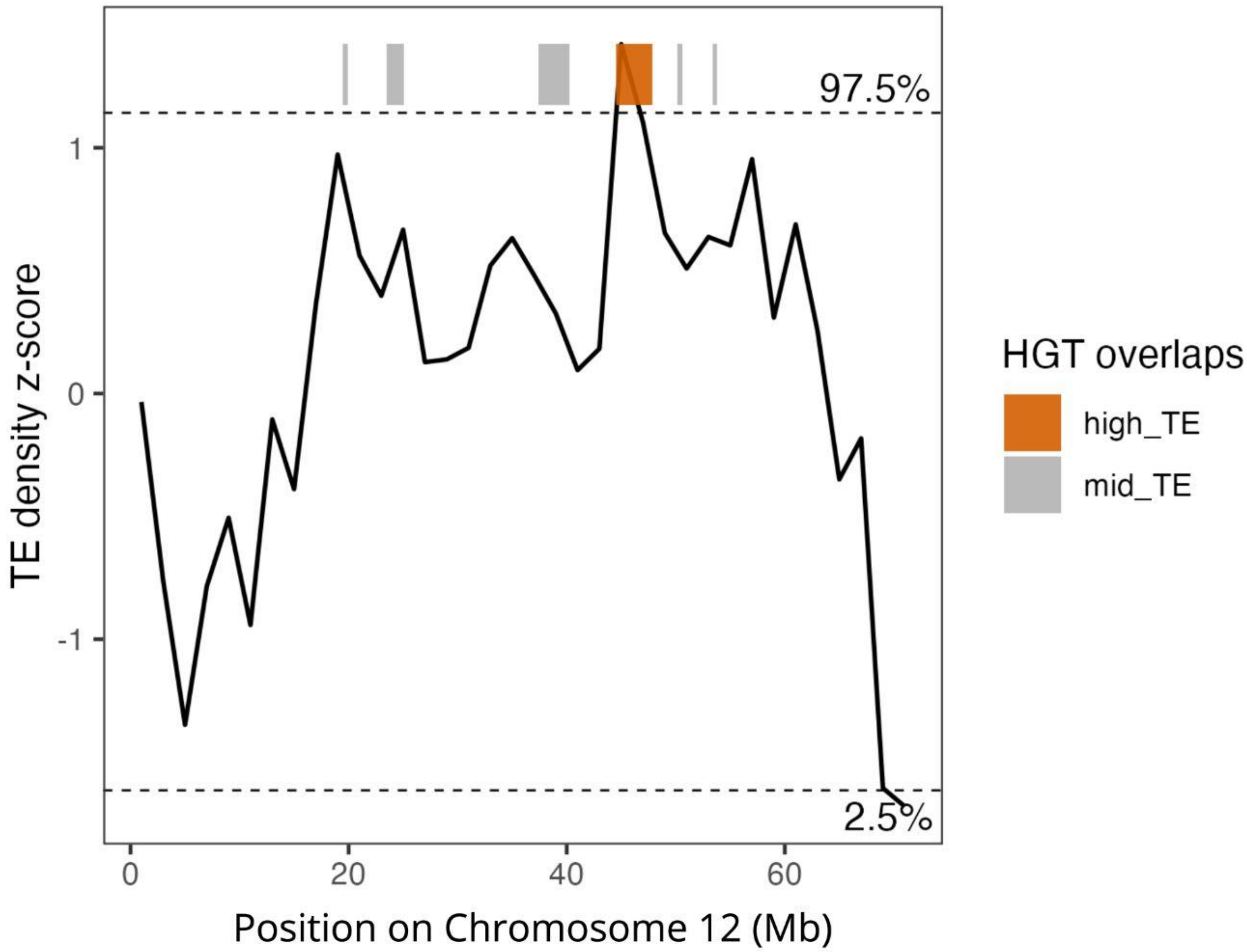

Supplementary Figure 25. Z-score transformed TE density by type in sliding 2 Mb windows across Chromosome 12 of *C. nanulus*. HGTs located within the upper (97.5th percentile) tail of the TE density distribution are indicated by orange bars. Gray bars indicate HGTs falling outside this tail.

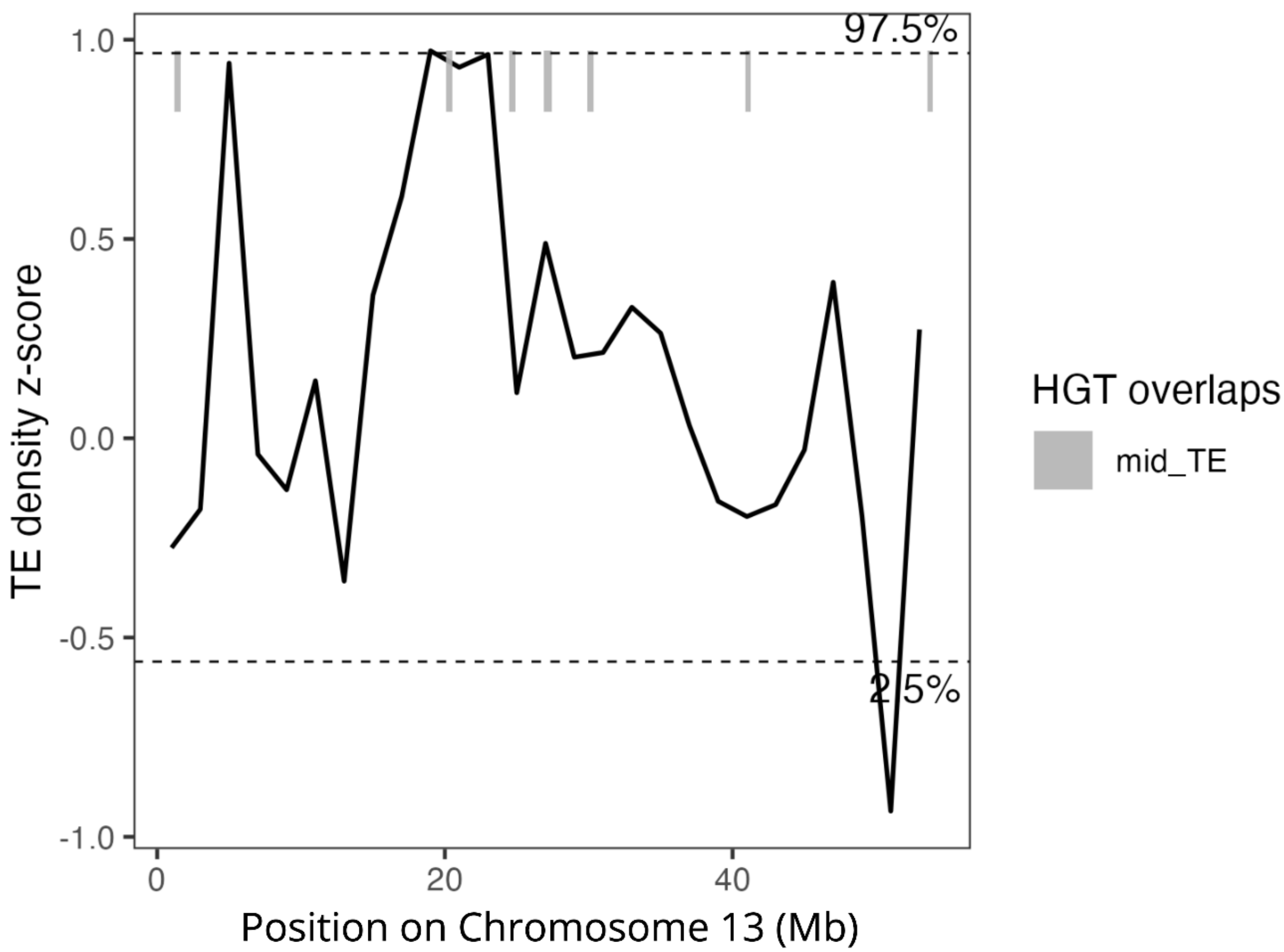

Supplementary Figure 26. Z-score transformed TE density by type in sliding 2 Mb windows across Chromosome 13 of *C. nanulus*. Horizontally transferred DNA sequences (HGTs) located outside of the tails of the TE density distribution are indicated with gray bars.

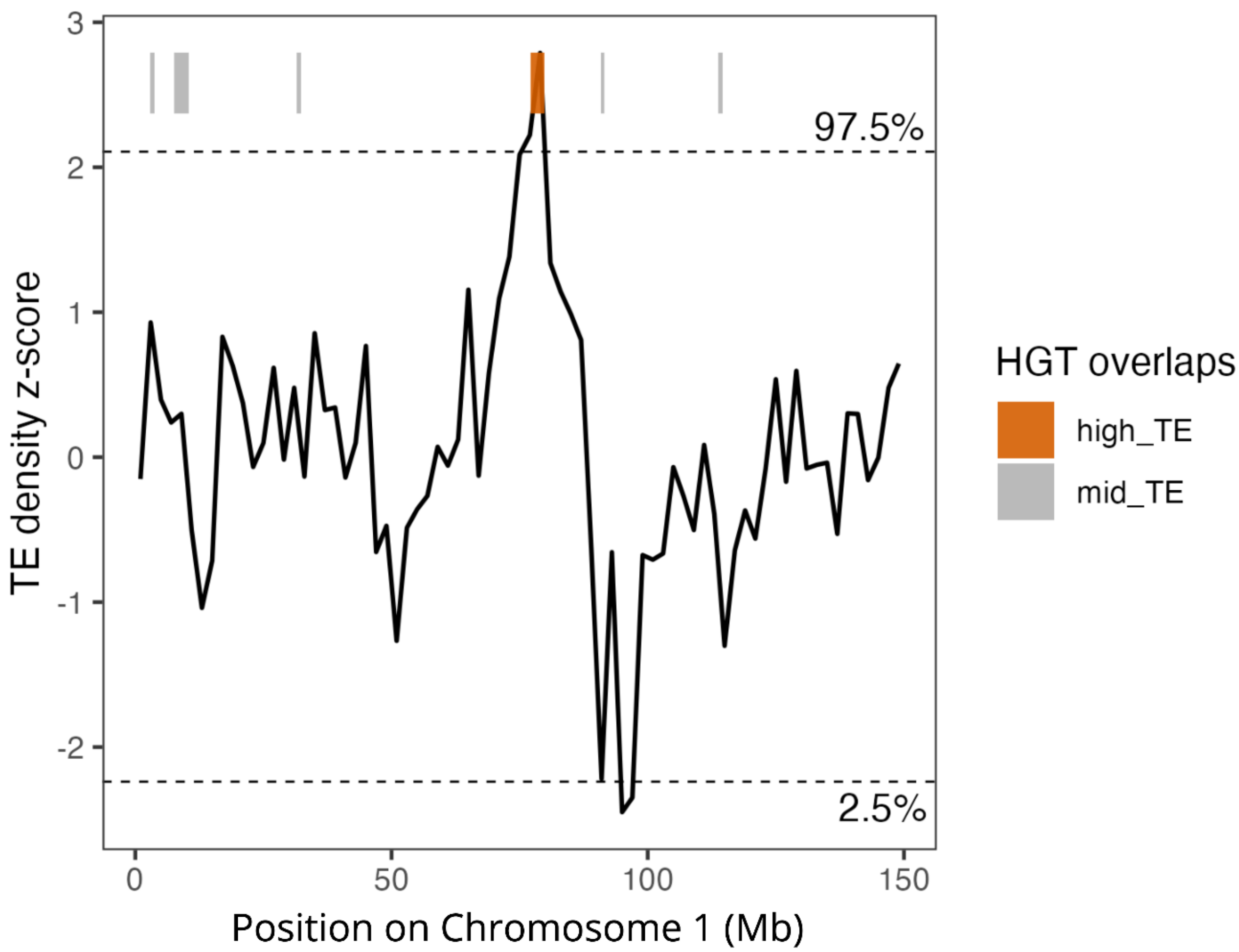

Supplementary Figure 27. Z-score transformed TE density by type in sliding 2 Mb windows across Chromosome 1 of *C. glandium*. HGTs located within the upper (97.5th percentile) tail of the TE density distribution are indicated by orange bars. Gray bars indicate HGTs falling outside this tail.

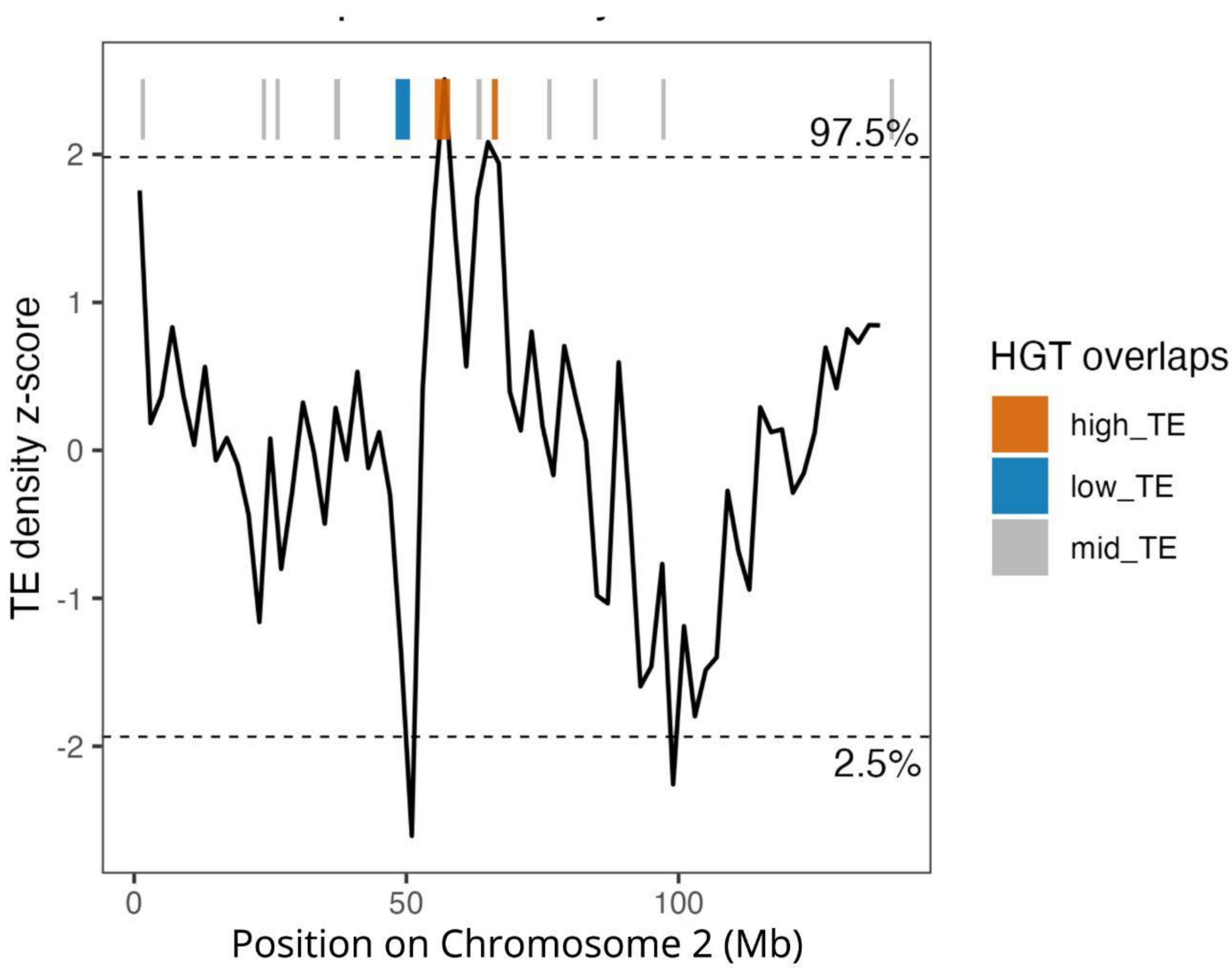

Supplementary Figure 28. Z-score transformed TE density by type in sliding 2 Mb windows across Chromosome 2 of *C. glandium*. HGTs located within the upper (97.5th percentile) and lower (2.5th percentile) tails of the TE density distribution are indicated by orange and blue bars, respectively. Gray bars indicate HGTs falling outside these tails.

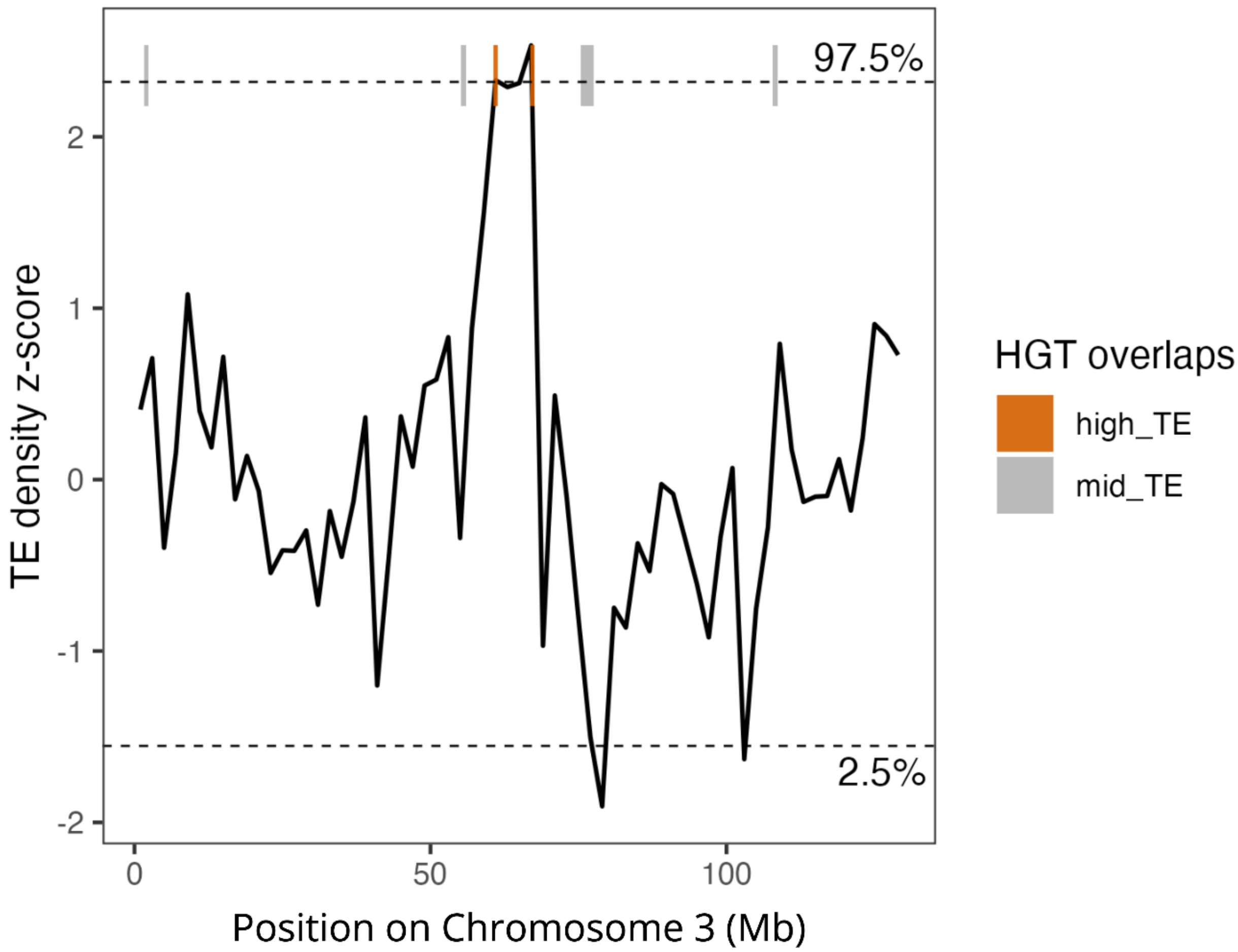

Supplementary Figure 29. Z-score transformed TE density by type in sliding 2 Mb windows across Chromosome 3 of *C. glandium*. HGTs located within the upper (97.5th percentile) tail of the TE density distribution are indicated by orange bars. Gray bars indicate HGTs falling outside this tail.

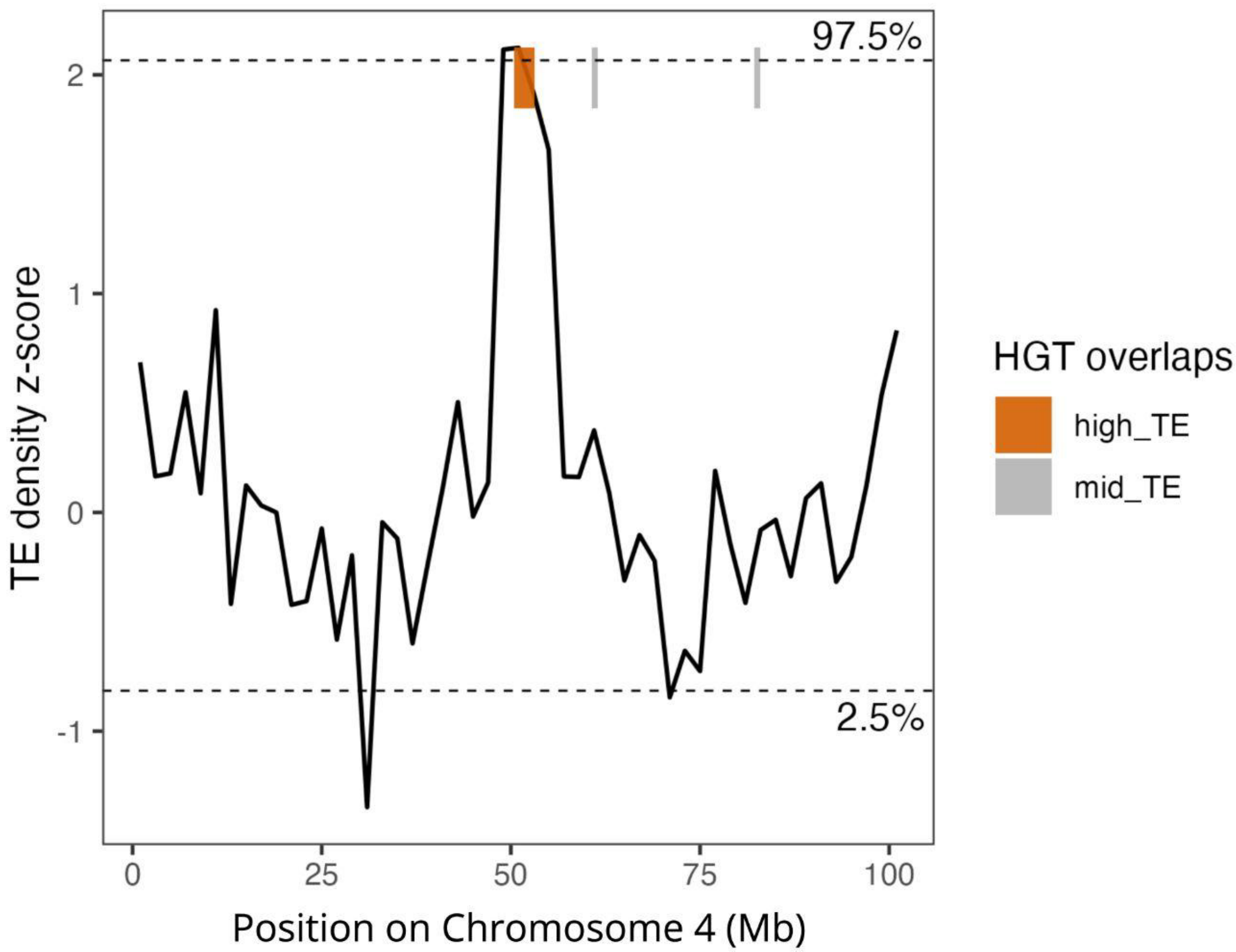

Supplementary Figure 30. Z-score transformed TE density by type in sliding 2 Mb windows across Chromosome 4 of *C. glandium*. HGTs located within the upper (97.5th percentile) tail of the TE density distribution are indicated by orange bars. Gray bars indicate HGTs falling outside this tail.

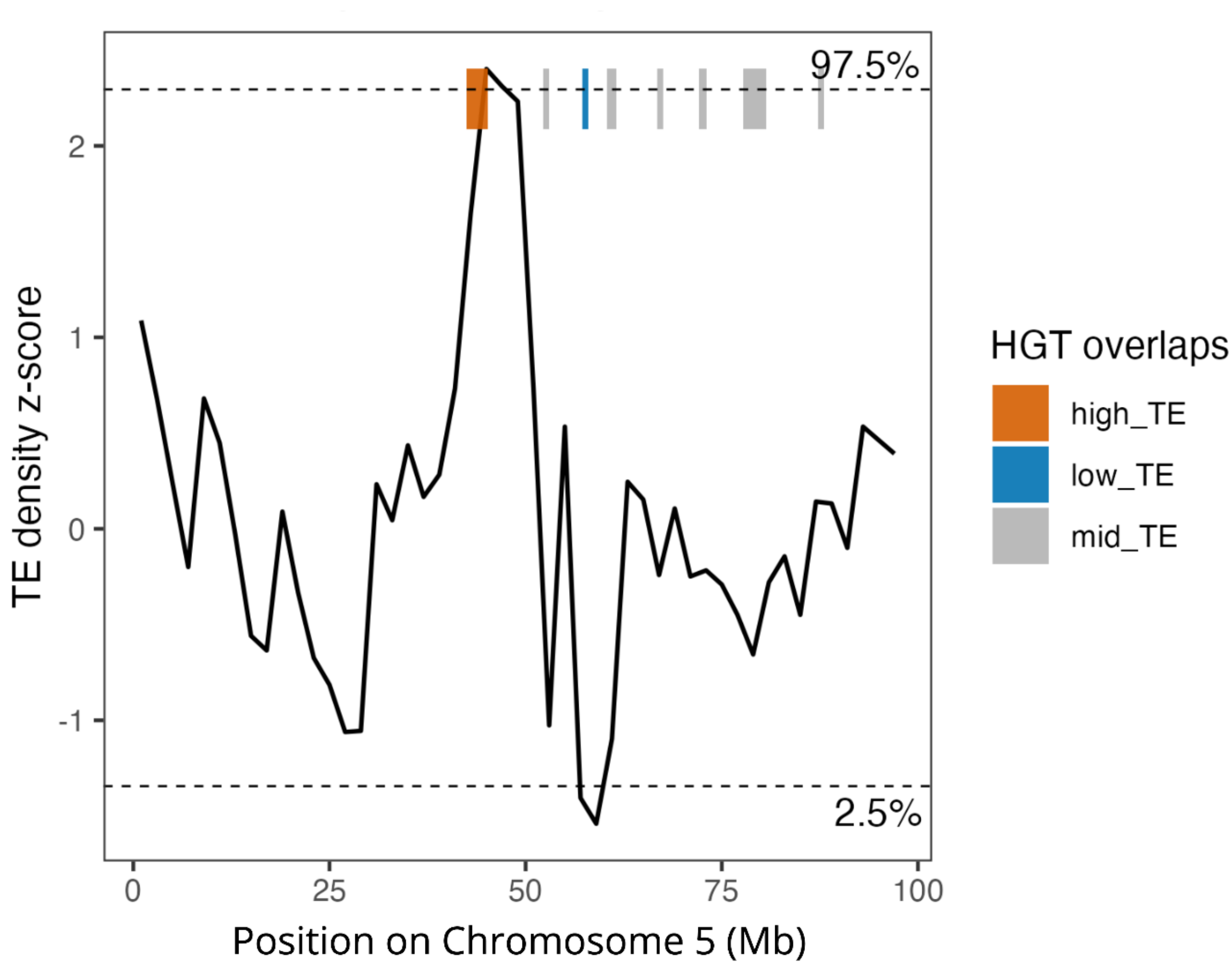

Supplementary Figure 31. Z-score transformed TE density by type in sliding 2 Mb windows across Chromosome 5 of *C. glandium*. HGTs located within the upper (97.5th percentile) and lower (2.5th percentile) tails of the TE density distribution are indicated by orange and blue bars, respectively. Gray bars indicate HGTs falling outside these tails.

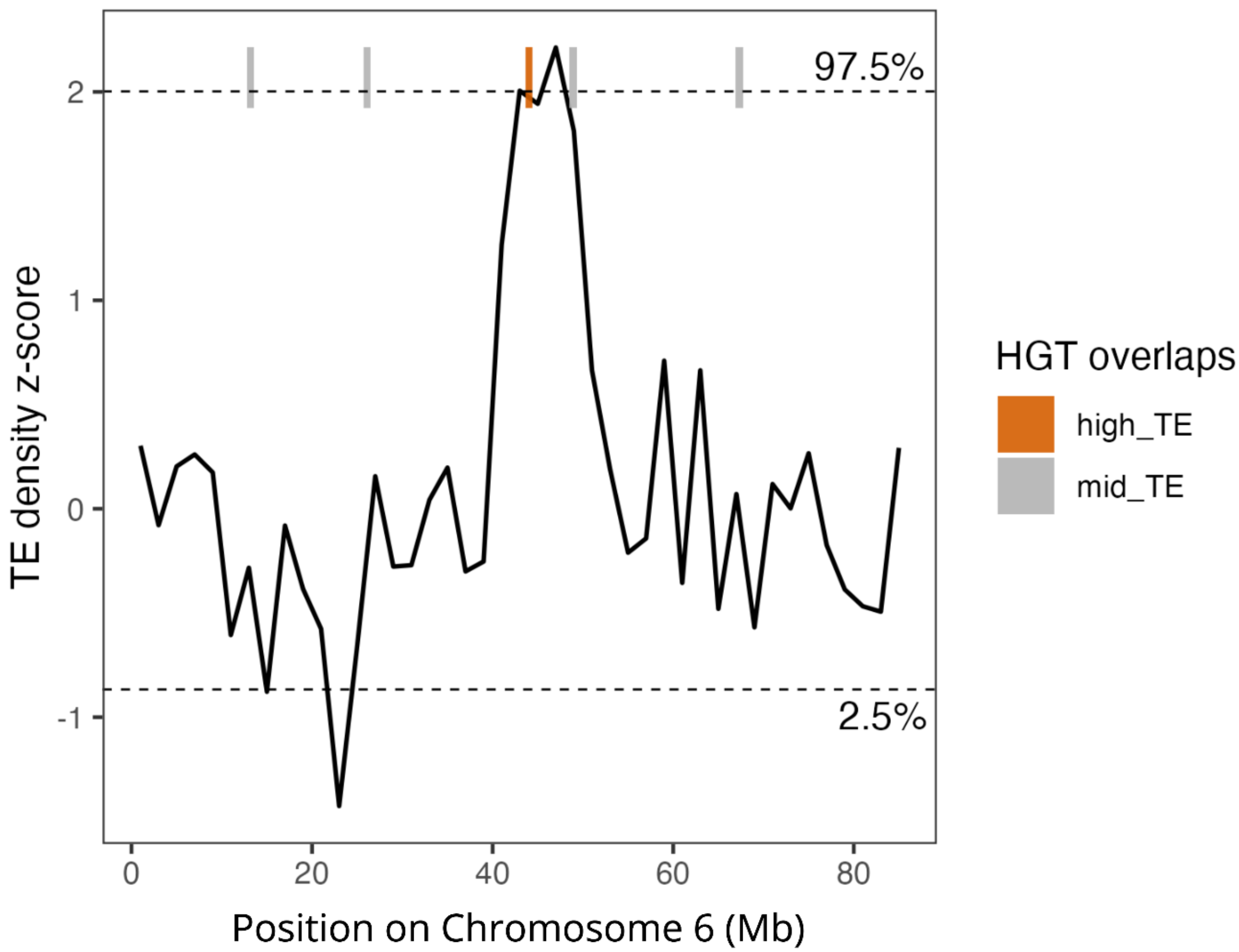

Supplementary Figure 32. Z-score transformed TE density by type in sliding 2 Mb windows across Chromosome 6 of *C. glandium*. HGTs located within the upper (97.5th percentile) tail of the TE density distribution are indicated by orange bars. Gray bars indicate HGTs falling outside this tail.

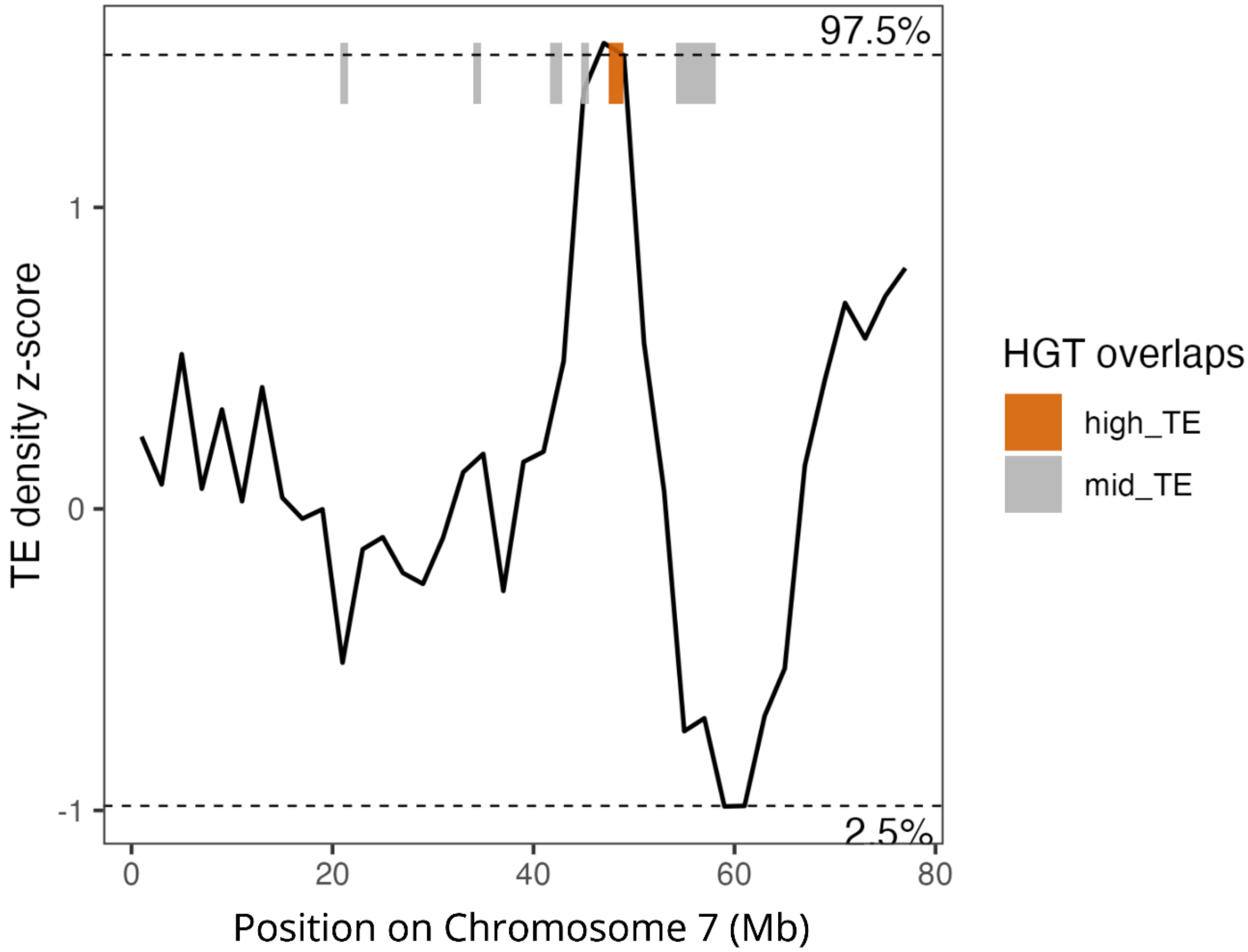

Supplementary Figure 33. Z-score transformed TE density by type in sliding 2 Mb windows across Chromosome 7 of *C. glandium*. HGTs located within the upper (97.5th percentile) tail of the TE density distribution are indicated by orange bars. Gray bars indicate HGTs falling outside this tail.

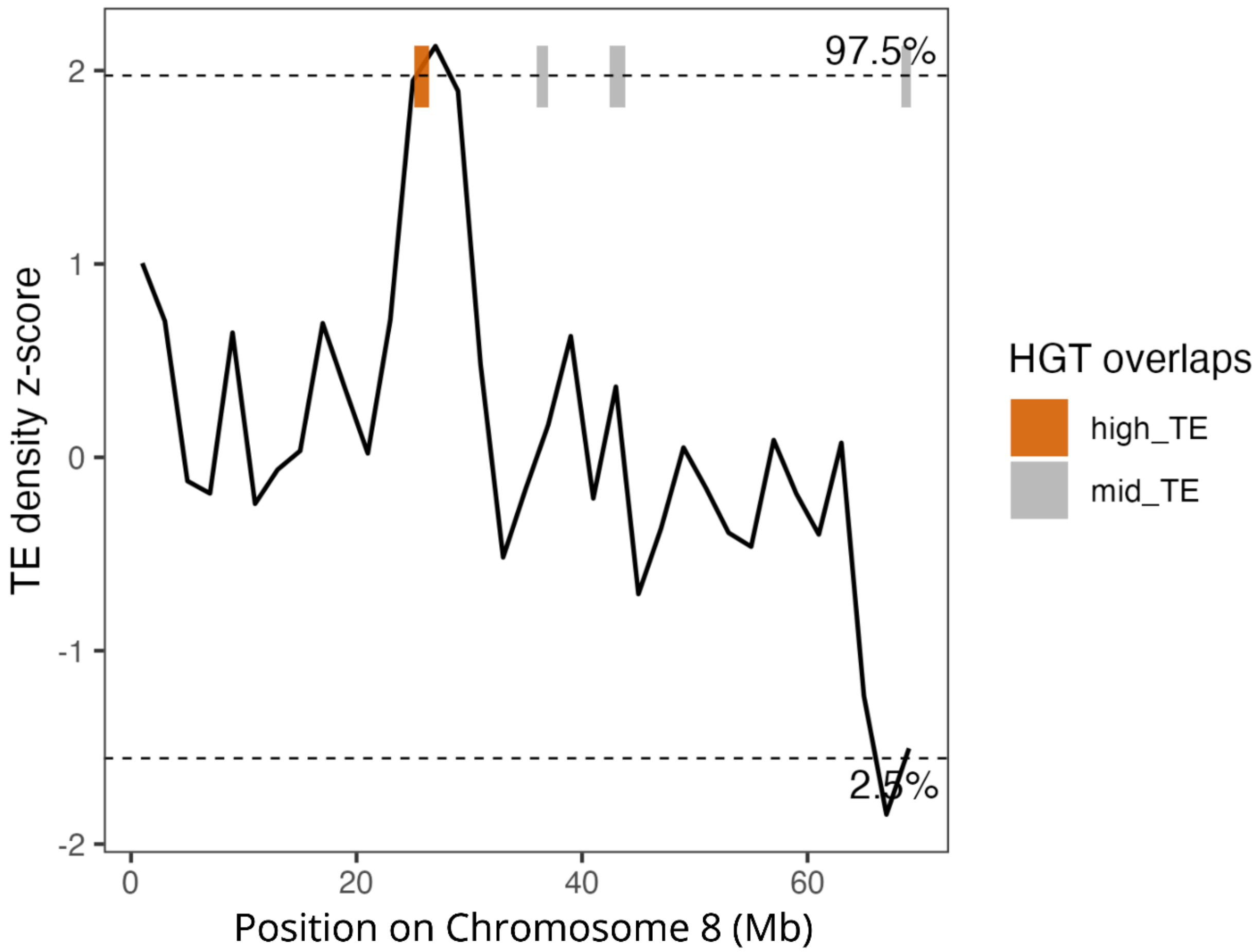

Supplementary Figure 34. Z-score transformed TE density by type in sliding 2 Mb windows across Chromosome 8 of *C. glandium*. HGTs located within the upper (97.5th percentile) tail of the TE density distribution are indicated by orange bars. Gray bars indicate HGTs falling outside this tail.

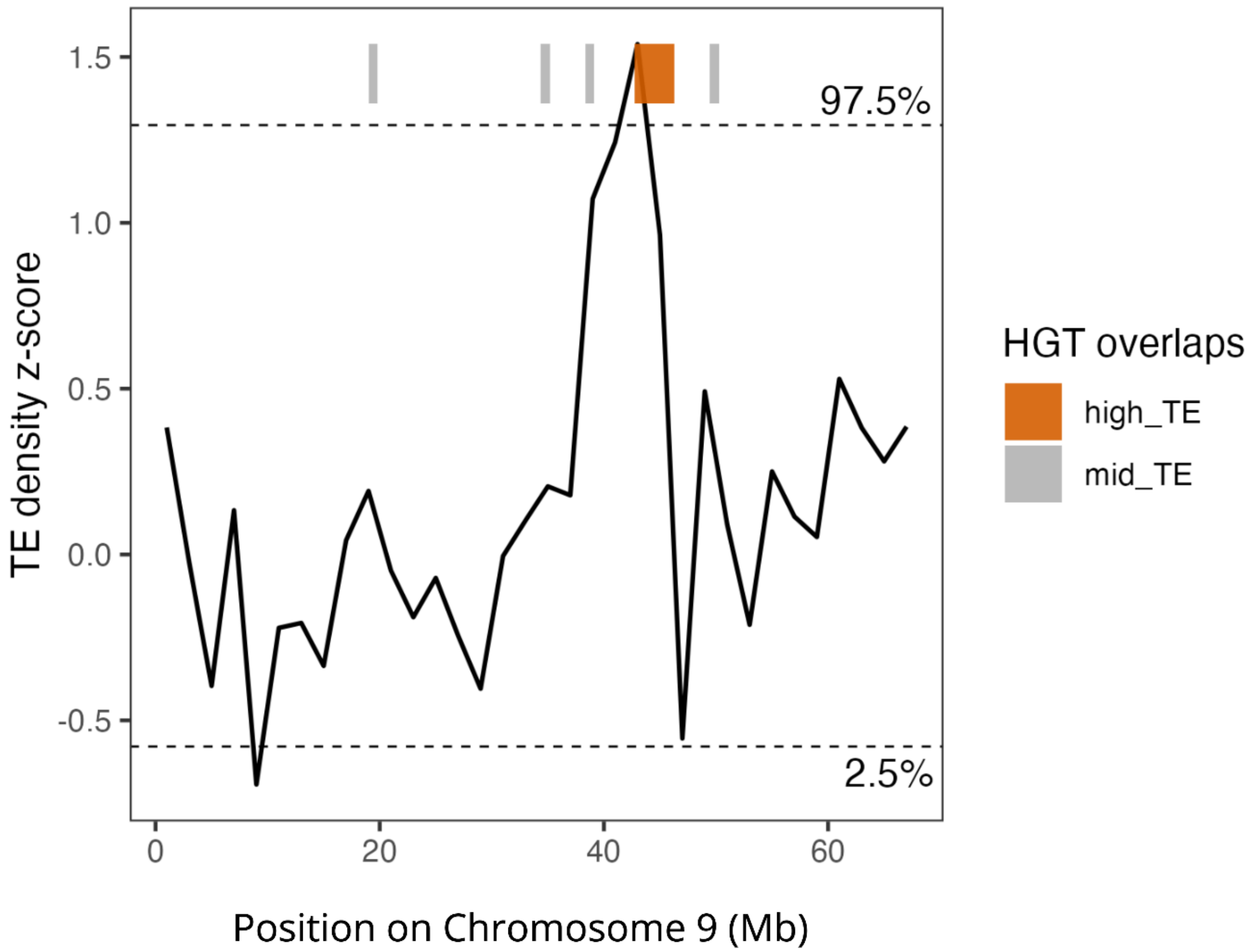

Supplementary Figure 35. Z-score transformed TE density by type in sliding 2 Mb windows across Chromosome 9 of *C. glandium*. HGTs located within the upper (97.5th percentile) tail of the TE density distribution are indicated by orange bars. Gray bars indicate HGTs falling outside this tail.

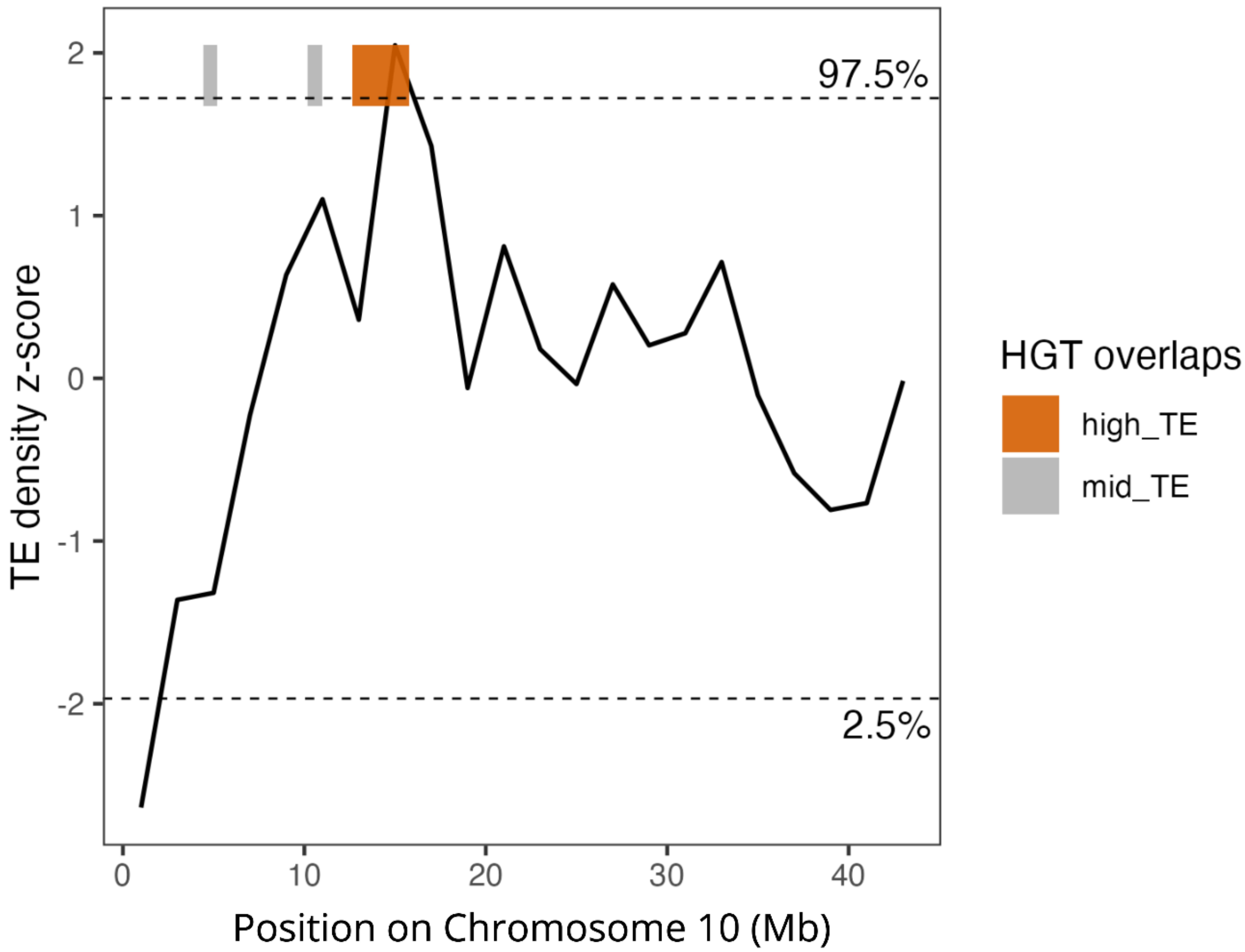

Supplementary Figure 36. Z-score transformed TE density by type in sliding 2 Mb windows across Chromosome 10 of *C. glandium*. HGTs located within the upper (97.5th percentile) tail of the TE density distribution are indicated by orange bars. Gray bars indicate HGTs falling outside this tail.

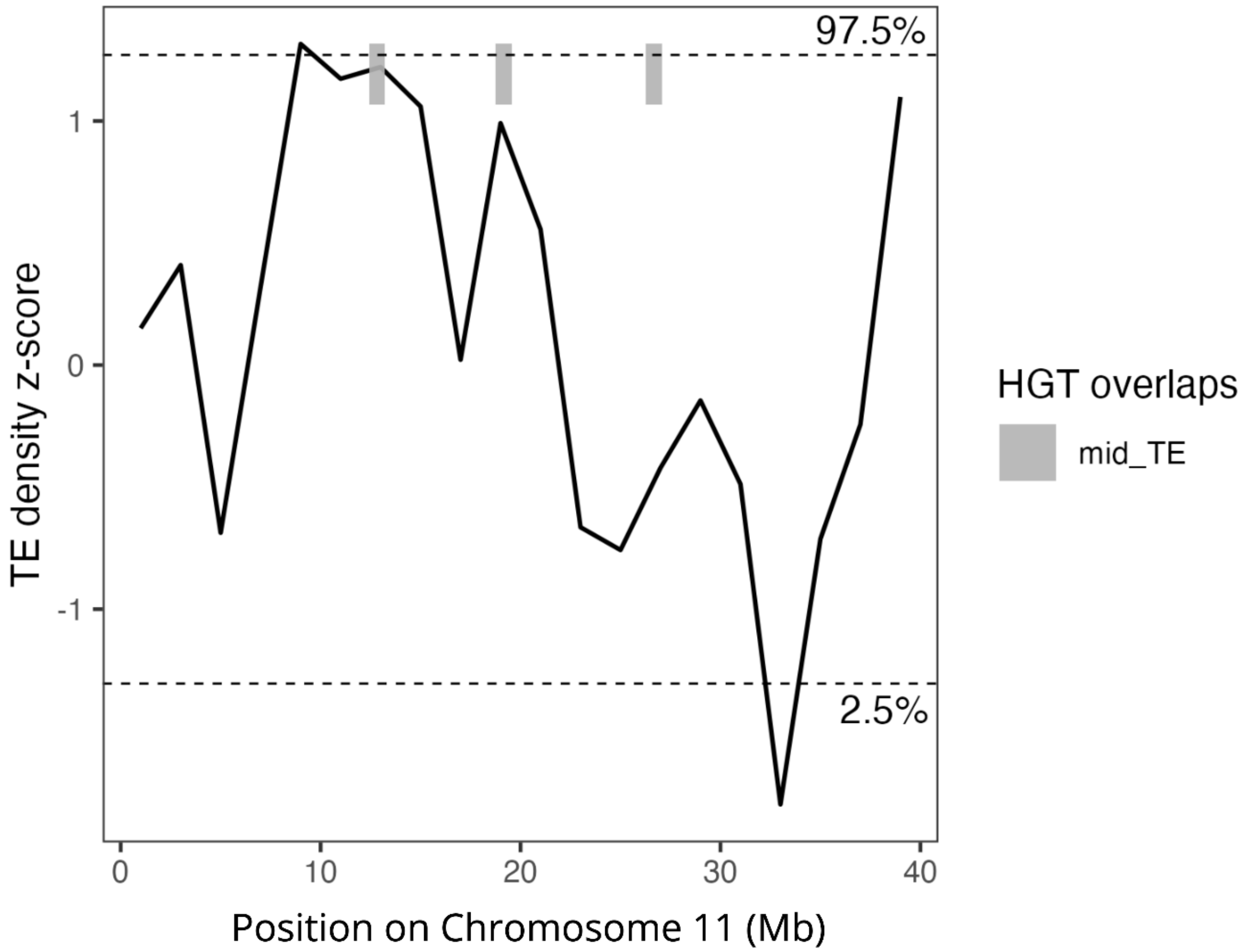

Supplementary Figure 37. Z-score transformed TE density by type in sliding 2 Mb windows across Chromosome 11 of *C. glandium*. Horizontally transferred DNA sequences (HGTs) located outside of the tails of the TE density distribution are indicated with gray bars.

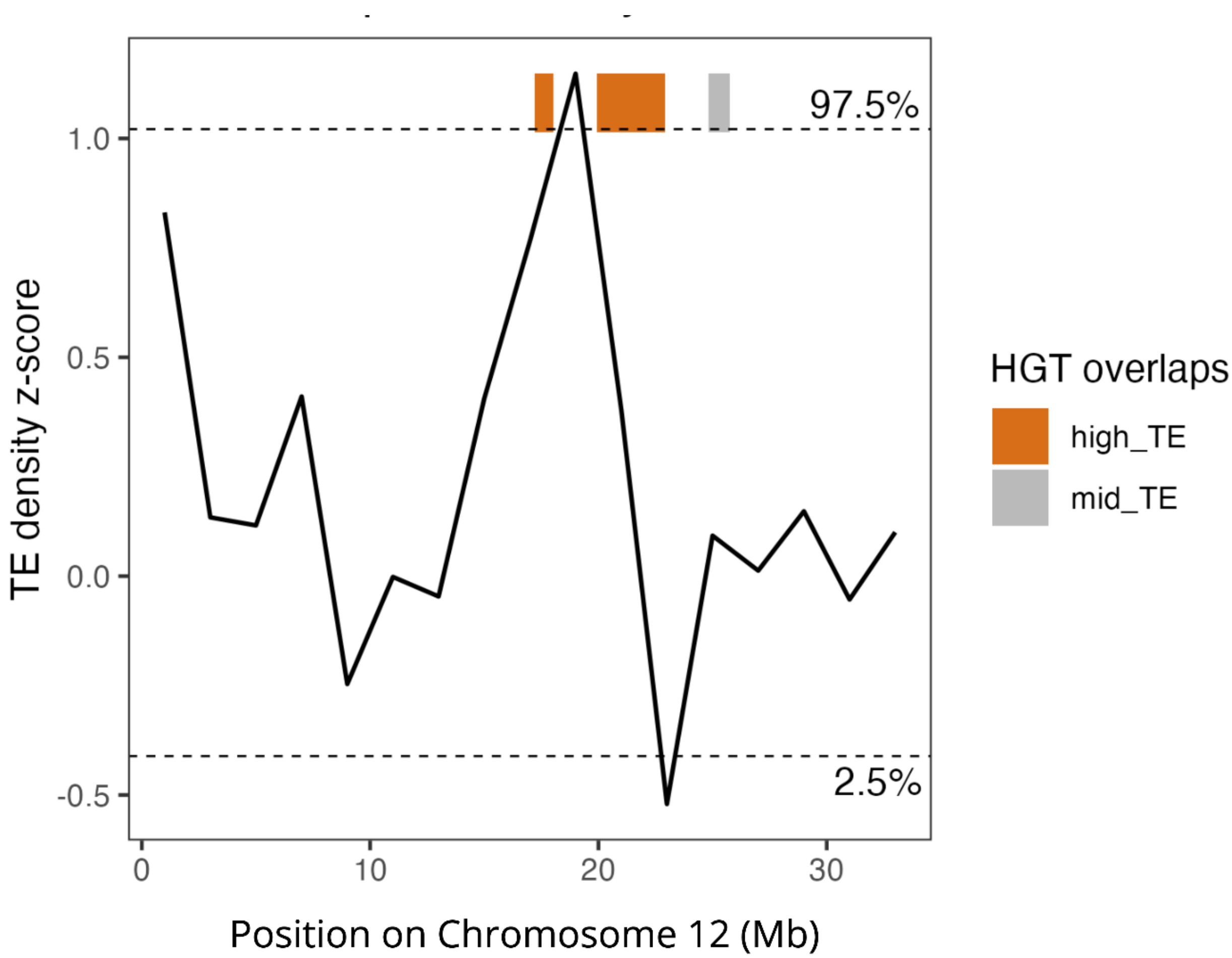

Supplementary Figure 38. Z-score transformed TE density by type in sliding 2 Mb windows across Chromosome 12 of *C. glandium*. HGTs located within the upper (97.5th percentile) tail of the TE density distribution are indicated by orange bars. Gray bars indicate HGTs falling outside this tail.

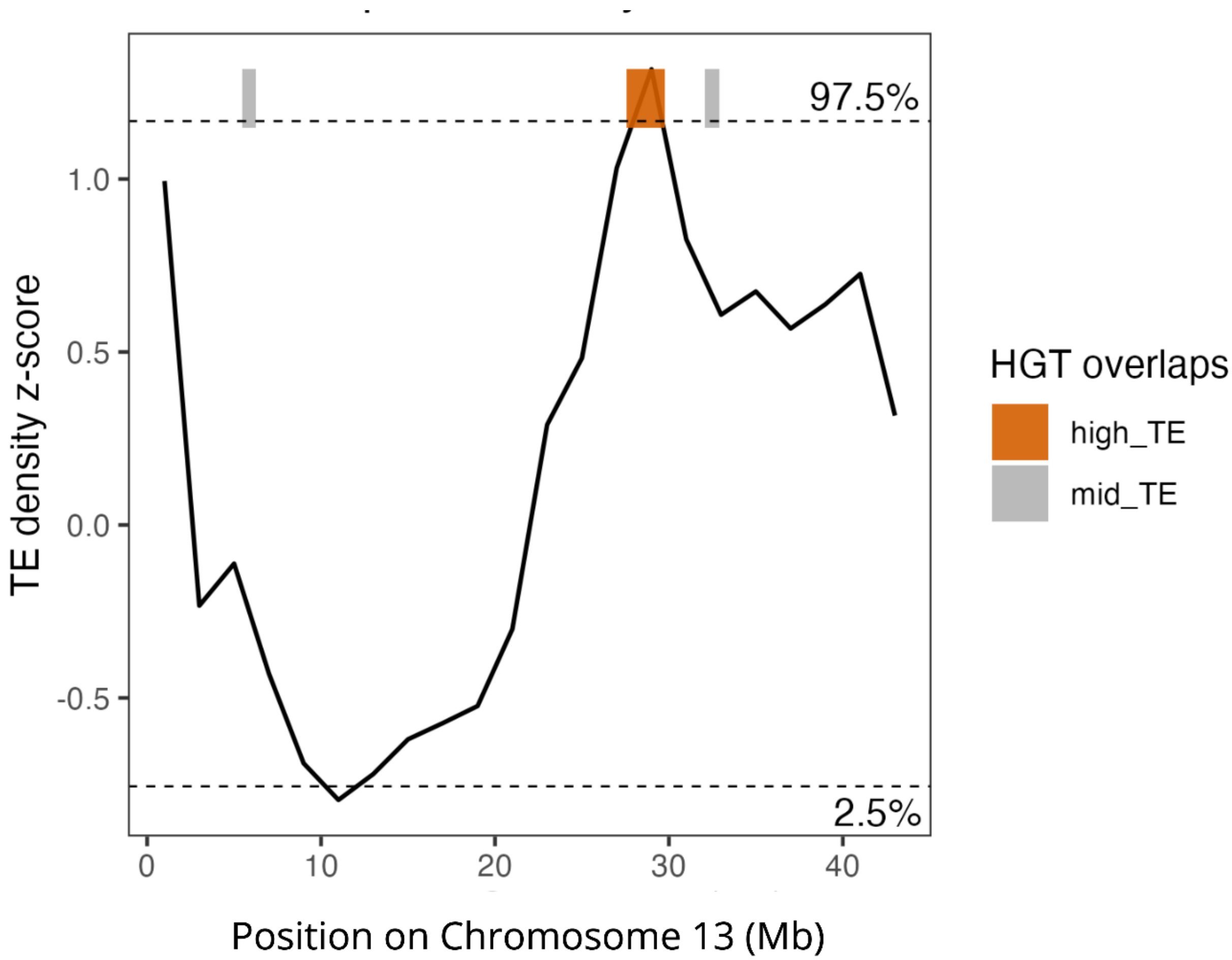

Supplementary Figure 39. Z-score transformed TE density by type in sliding 2 Mb windows across Chromosome 13 of *C. glandium*. HGTs located within the upper (97.5th percentile) tail of the TE density distribution are indicated by orange bars. Gray bars indicate HGTs falling outside this tail.

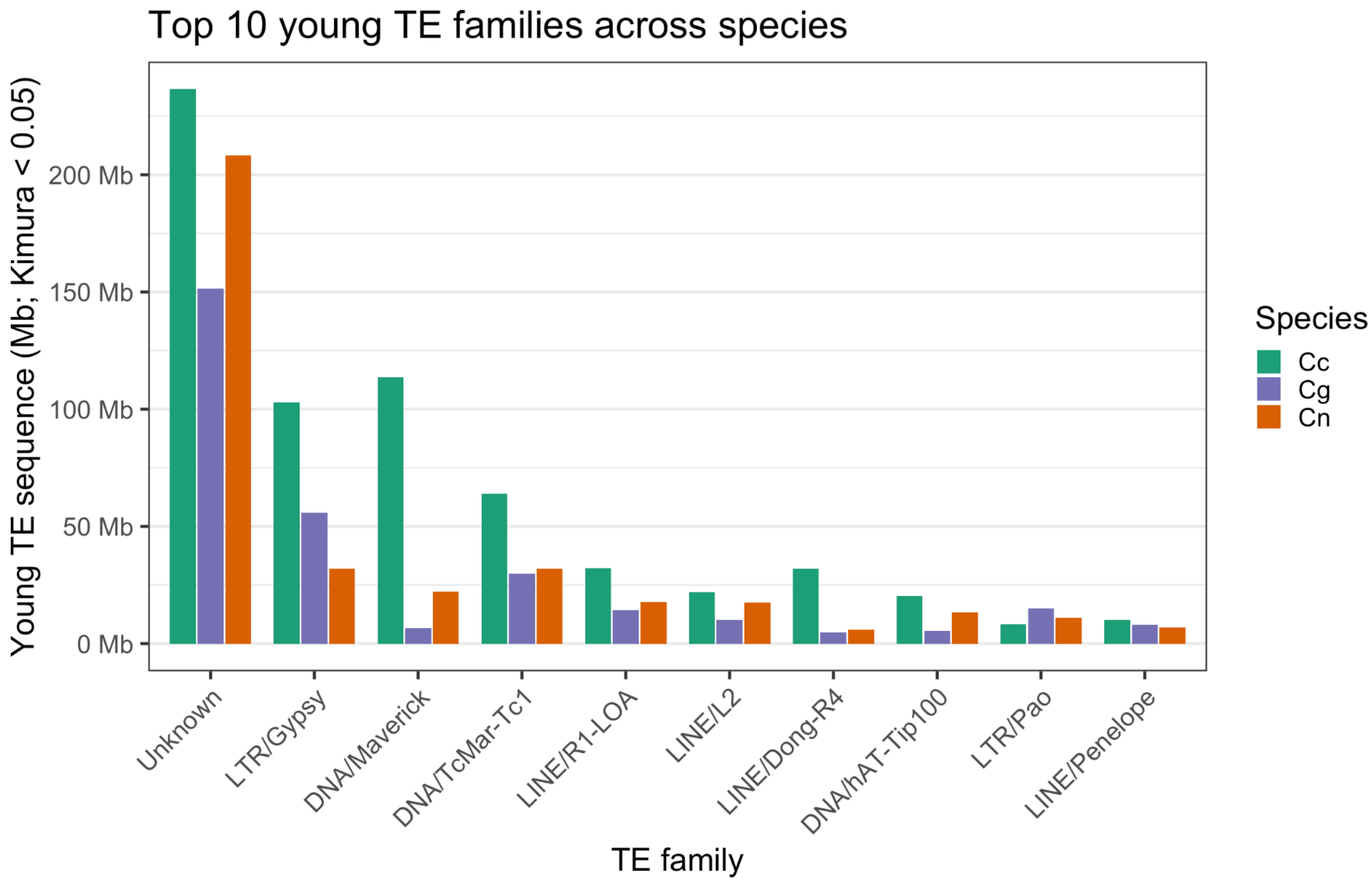

Supplementary Figure 40. Top 10 young (Kimura $D < 0.05$) genome-wide TE families across *Curculio* species: *C. caryae* (Cc, teal), *C. glandium* (Cg, purple), and *C. nanulus* (Cn, orange).

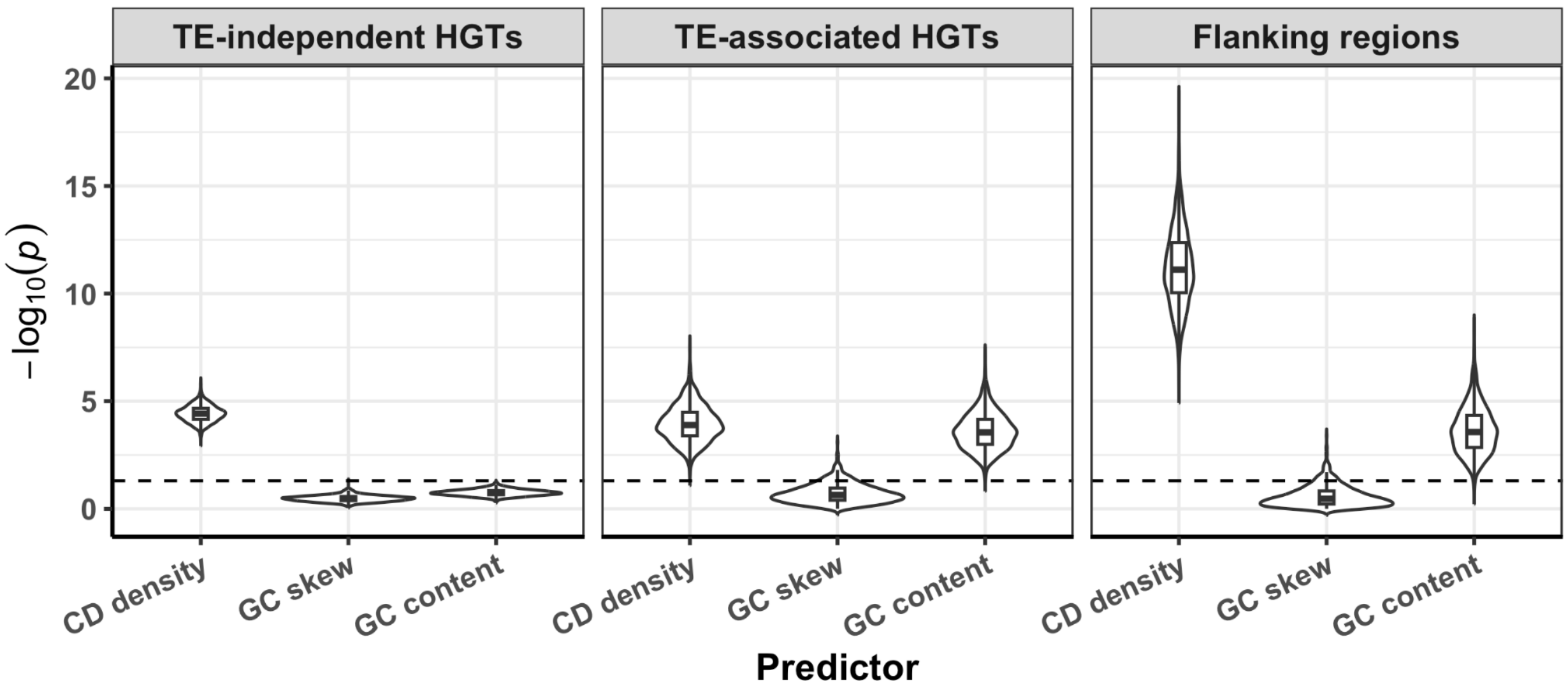

Supplementary Figure 41. Violin plots with embedded box plots summarizing the distributions of *z*-test -$\log_{10}$(*p*-values) for testing the null hypothesis that the regression coefficient β_j for a given predictor *j* is zero (H_0: $\beta_j = 0$), with the alternative hypothesis that β_j is not equal to zero (H_0: $\beta_j \neq 0$). The distribution summaries are based on 500 chromosome-matched multinomial regression replicates. Each model had three predictors: coding sequence (CD) density, GC skew, and GC content. The models predicted four response classes: TE-independent HGT cores, TE-associated HGT cores, HGT flanking windows, and chromosome-matched background windows as the reference class. The horizontal dashed lines denote the significance threshold of -$\log_{10}$(0.05).

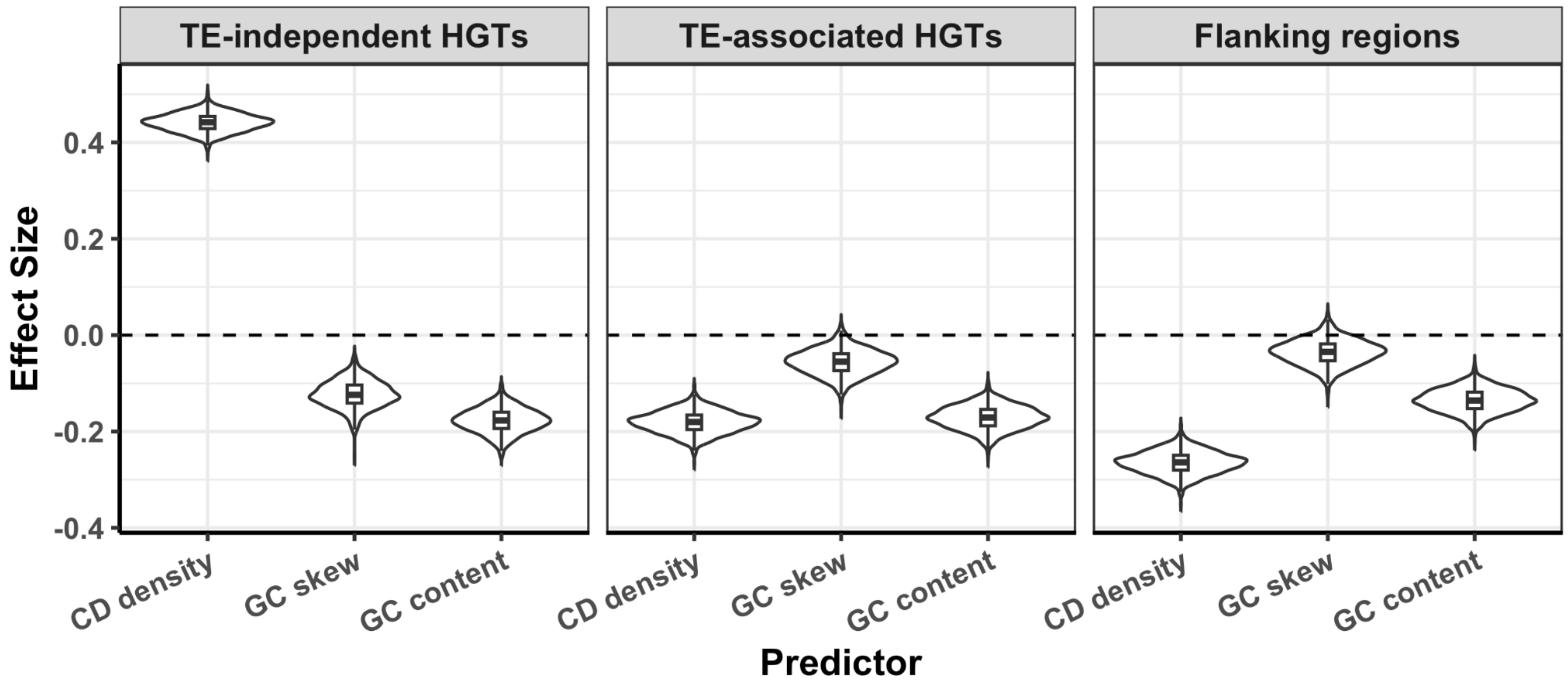

Supplementary Figure 42. Violin plots with embedded box plots summarizing the distributions of predictor effect sizes (regression coefficient values) across 500 chromosome-matched multinomial regression replicates. Each model had three predictors: coding sequence (CD) density, GC skew, and GC content. The models predicted four response classes: TE-associated HGT cores, TE-independent HGT cores, HGT flanking windows, and chromosome-matched background windows as the reference class. Positive coefficients indicate increased log odds of assignment to that response class for windows with higher values of the corresponding predictor, whereas negative coefficients indicate decreased log odds, relative to background. The horizontal dashed lines denote a regression coefficient of zero, indicating no association between the predictor and the response class.

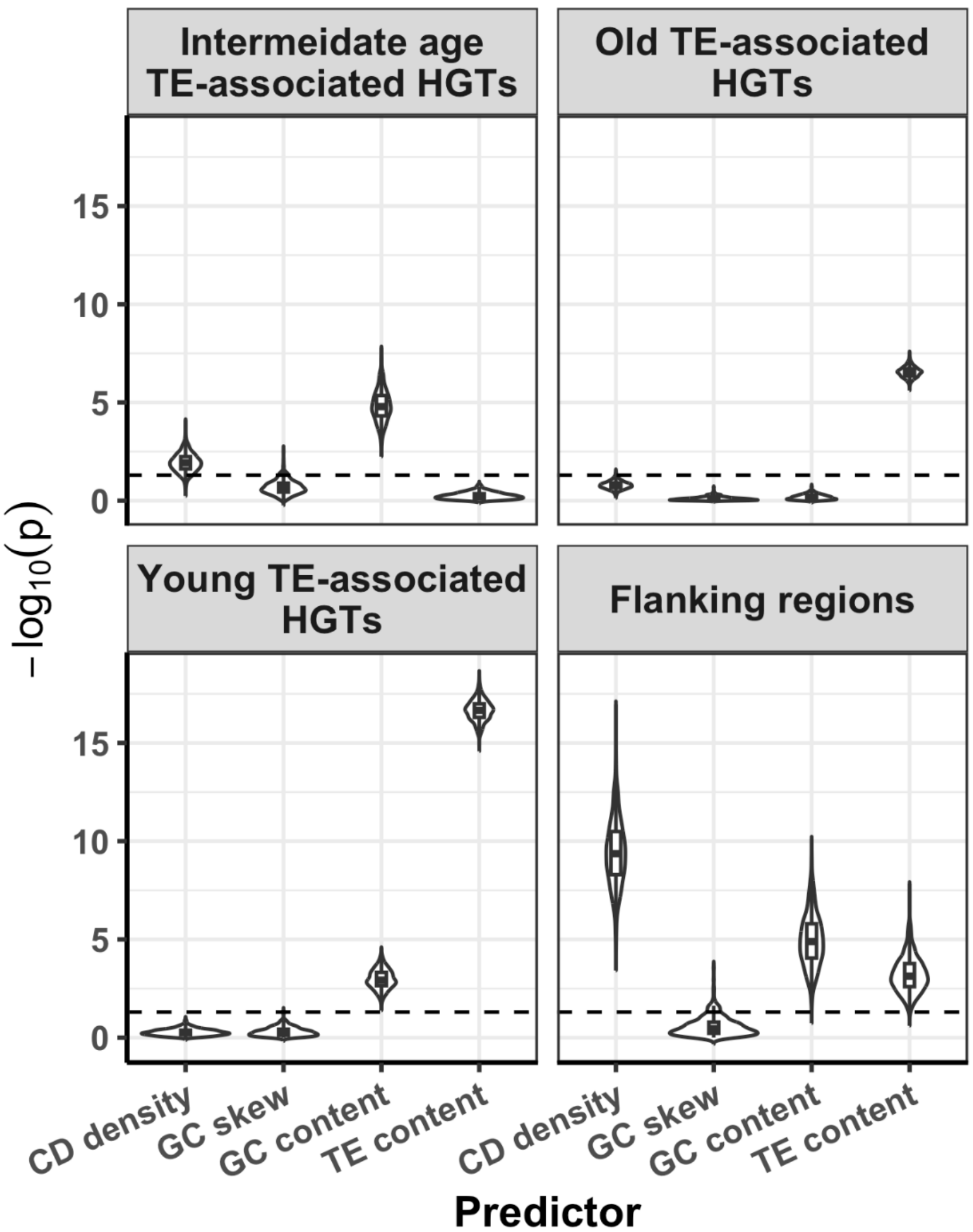

Supplementary Figure 43. Violin plots with embedded box plots summarizing the distributions of *z*-test -$\log_{10}$ (*p*-values) for testing the null hypothesis that the regression coefficient β_j for a given predictor *j* is zero (H_0: $\beta_j = 0$), with the alternative hypothesis that β_j is not equal to zero (H_0: $\beta_j \neq 0$). The distribution summaries are based on 500 chromosome-matched multinomial regression replicates. Each model had four predictors: coding sequence (CD) density, GC skew, GC content, and TE content. The models predicted five response classes: TE-associated HGT cores based on TE age as young, intermediate, and old, HGT flanking windows, and chromosome-matched background windows as the reference class. The horizontal dashed lines denote the significance threshold of -$\log_{10}(0.05)$.

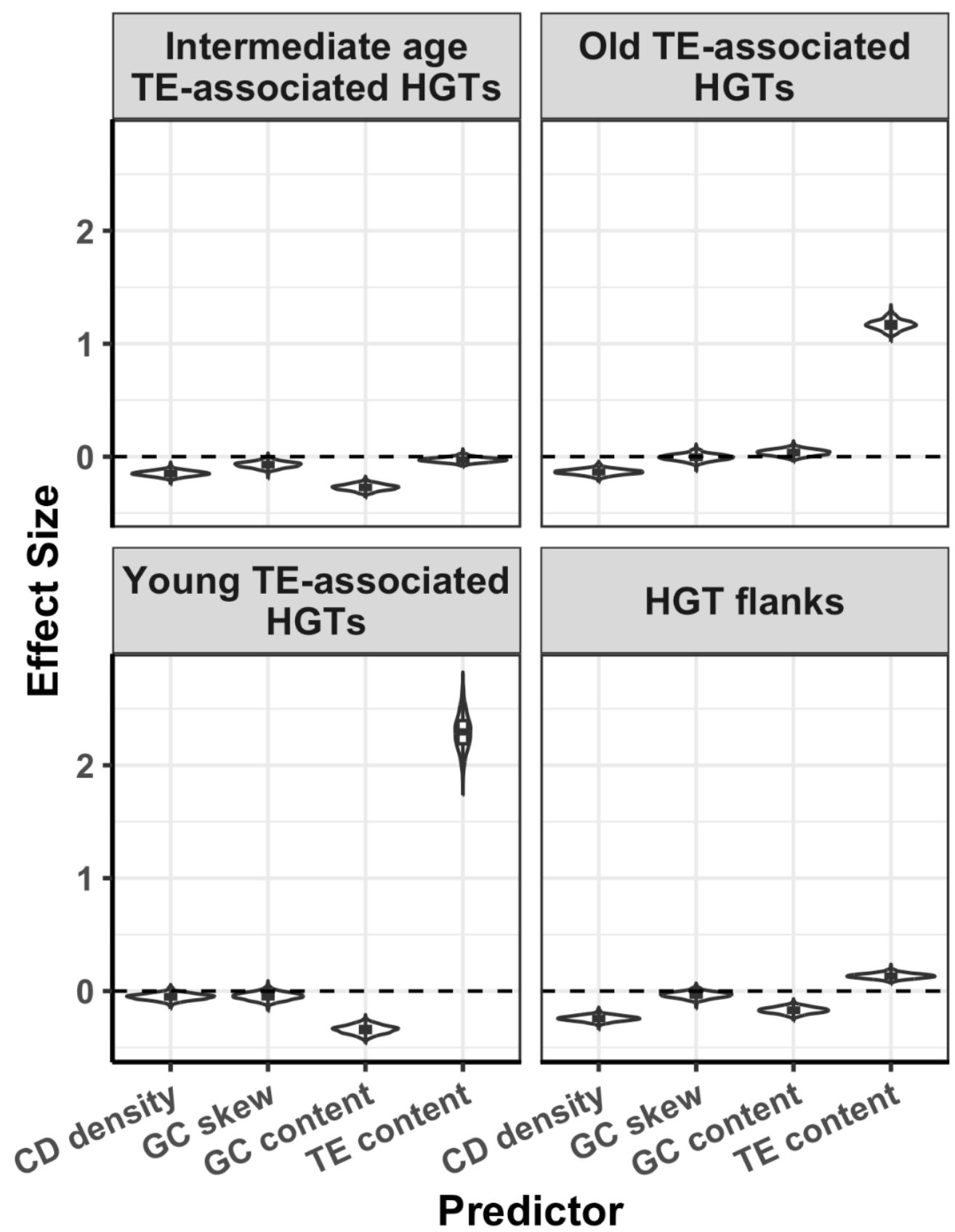

Supplementary Figure 44. Violin plots with embedded box plots summarizing the distributions of predictor effect sizes (regression coefficient values) across 500 chromosome-matched multinomial regression analyses. Each model had five predictors: coding sequence (CD) density, GC skew, GC content, and TE content. The models predicted five response classes: TE-associated HGT cores based on TE age as young, intermediate, and old, HGT flanking windows, and chromosome-matched background windows as the reference class. Positive coefficients indicate increased log odds of assignment to that response class for windows with higher values of the corresponding predictor, whereas negative coefficients indicate decreased log odds, relative to background. The horizontal dashed lines denote a regression coefficient of zero, indicating no association between the predictor and the response class.

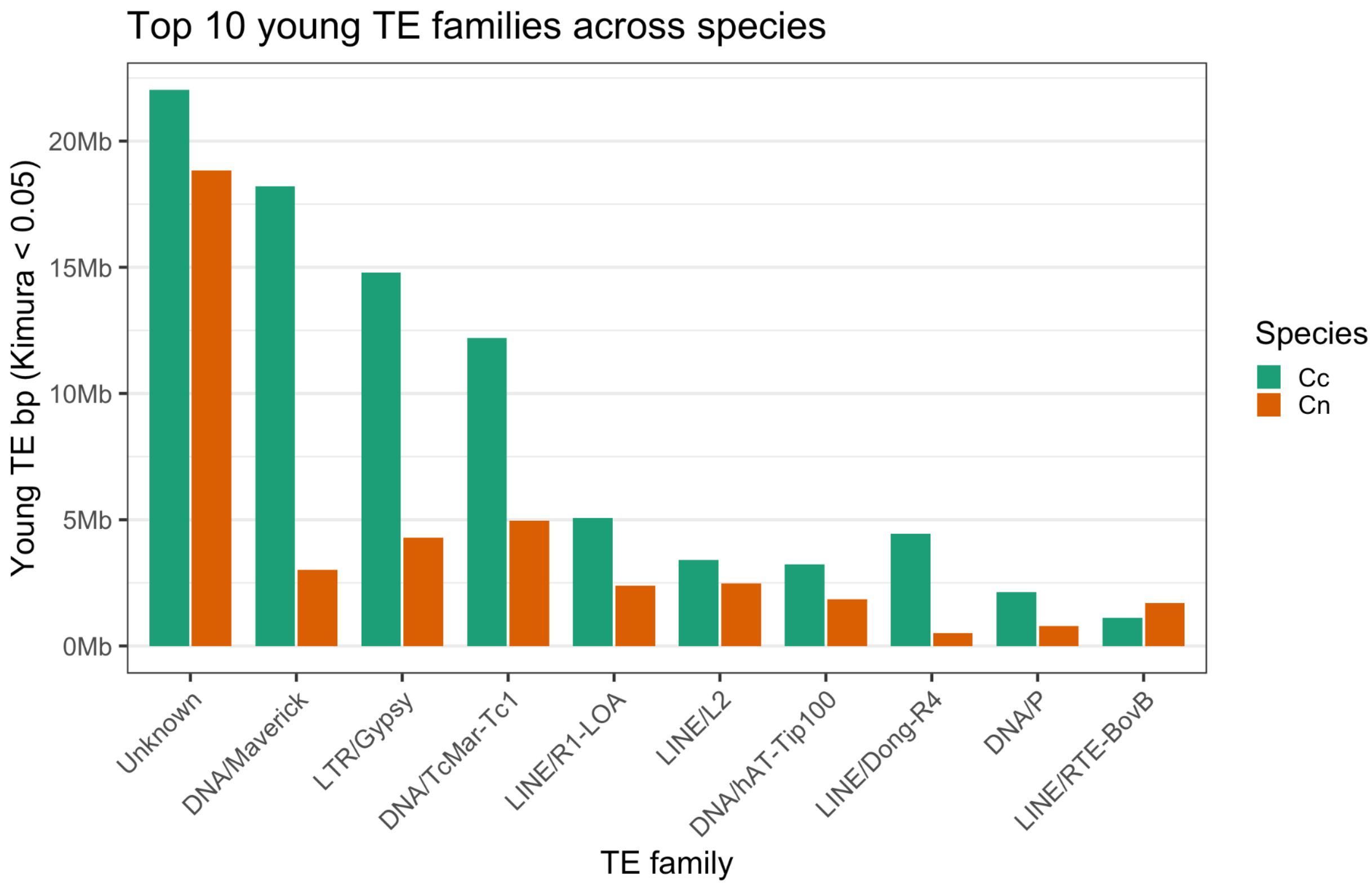

Supplementary Figure 45. Top 10 young (Kimura *D* < 0.05) node-specific TE families in *C. caryae* (Cc, teal) and *C. nanulus* (Cn, orange).

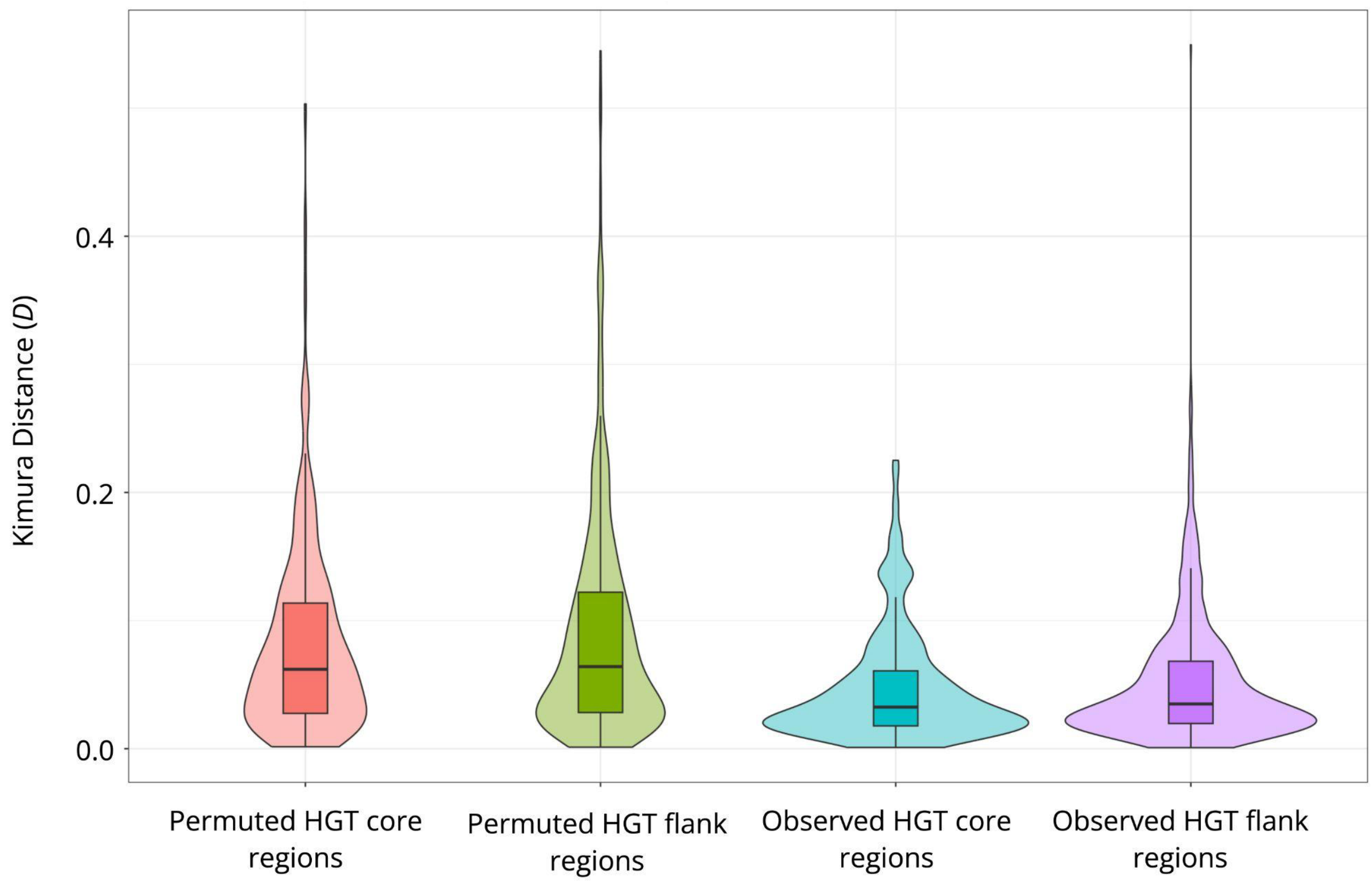

Supplementary Figure 46. Violin plots with embedded box plots summarizing the distribution of Maverick ages (Kimura *D*) across permuted HGT core, permuted HGT flank, observed HGT core, and observed HGT flank regions.

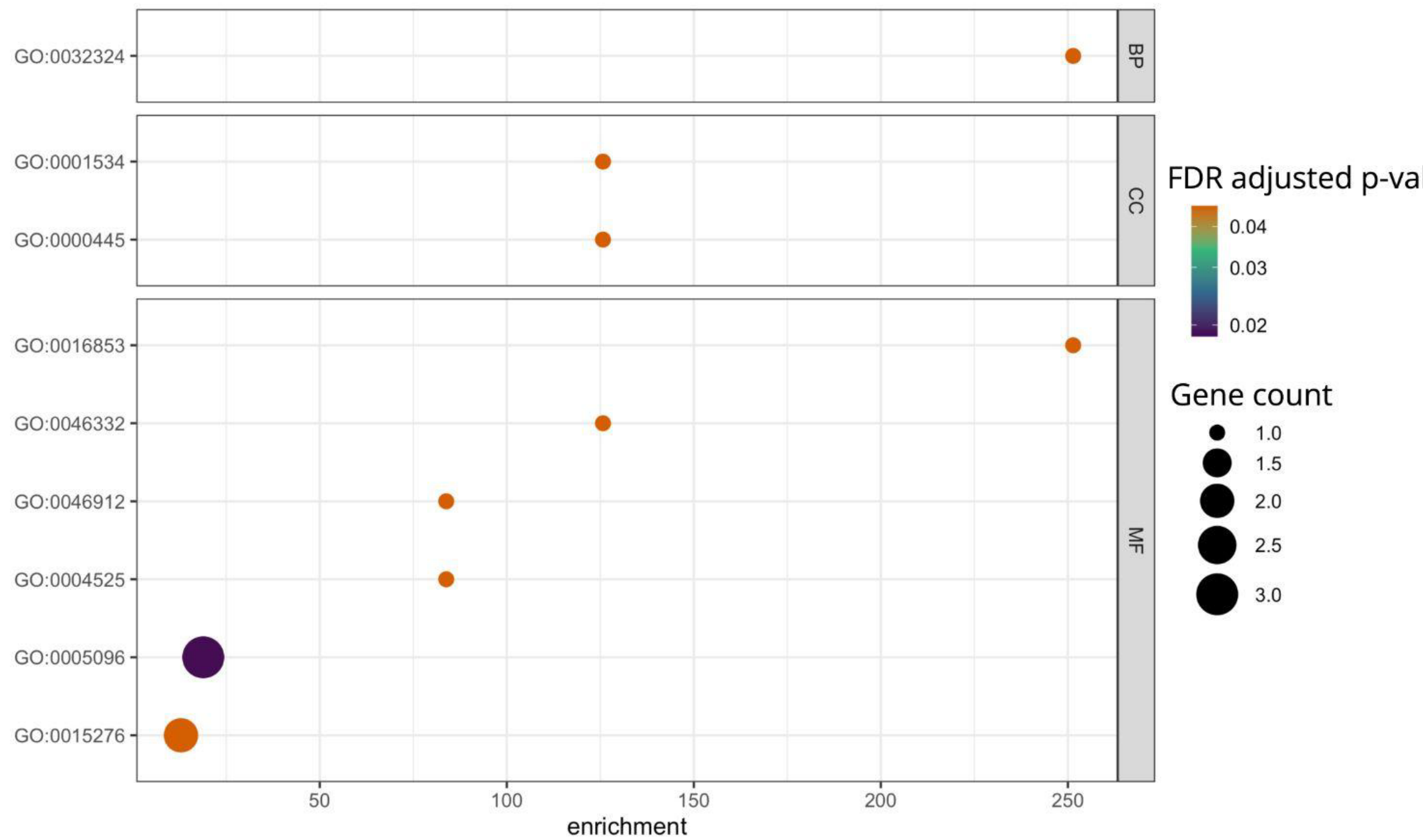

Supplemental Figure 47. Enriched Gene Ontology (GO) terms associated with HGTs in *C. caryae* by category: BP = biological process, CC = cellular component, and MF = molecular function. Gradient coloration represents FDR-adjusted *p*-values, and gene count indicates the number of genes associated with each enriched term.

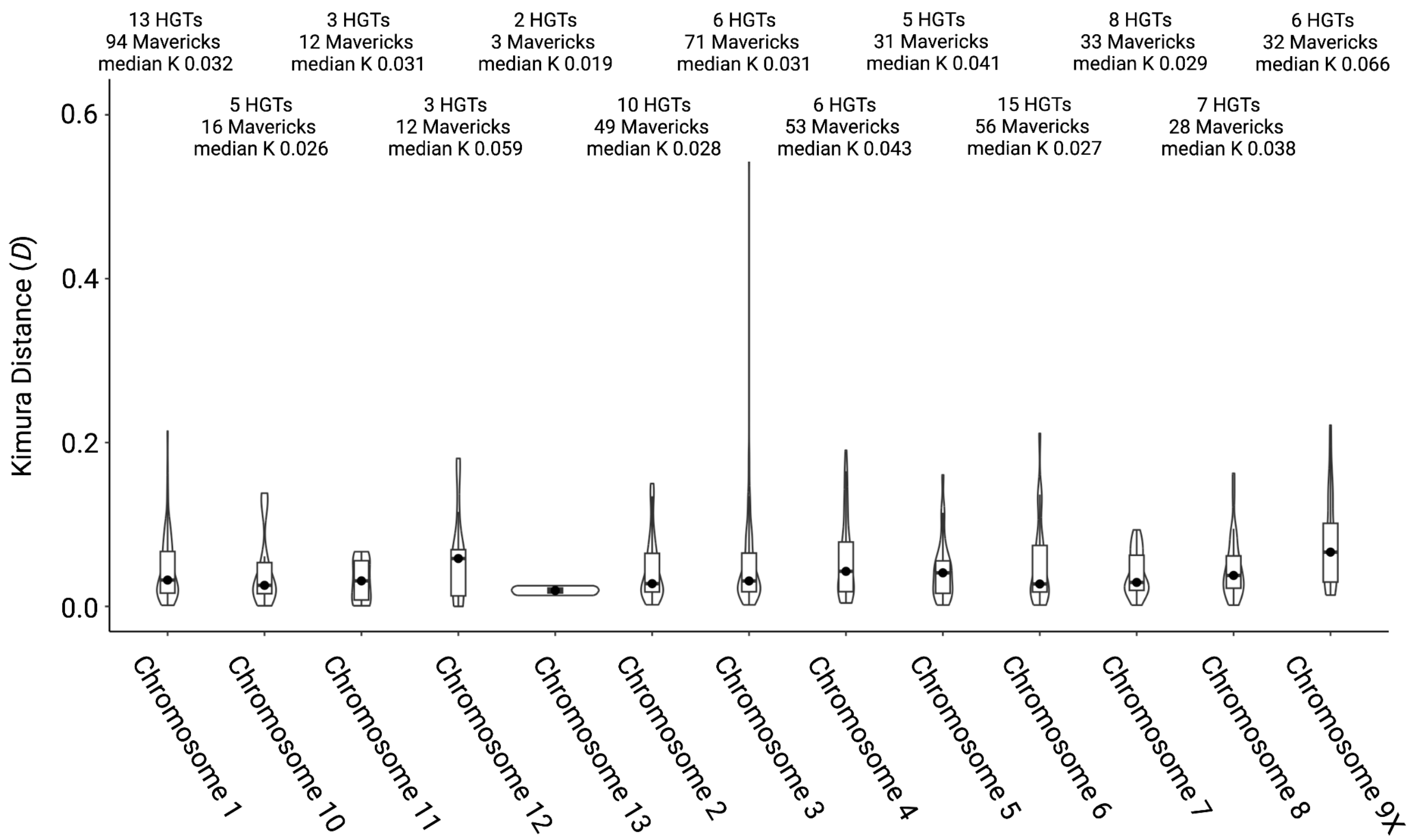

Supplemental Figure 48. Violin plots with embedded box plots summarizing the distribution of Kimura *D* estimates for Mavericks encapsulating HGTs in *C. caryae*. Chromosome 9 corresponds to the X chromosome.

Supplementary Table 1. Counts and cumulative sizes of structural variants (SVs), TEs and repeats, and HGT regions across homologous chromosomes in *Curculio* species: *C. caryae* (Cc), *C. nanulus* (Cn), and *C. glandium* (Cg).

Homologous chromosome	Species-specific SVs >100 bp (size in Mb)	TEs and repeats (size in Mb)	HGT regions (size in kb)
Cc: 1	21,047 (263.09)	355,659 (237.79)	235 (928.06)
Cn: 1	23,759 (143.80)	247,606 (133.89)	26 (87.353)
Cg: 2	2,233 (137.69)	225,385 (96.01)	18 (58.05)
Cc: 2	37,472 (223.79)	355,564 (225.38)	75 (201.28)
Cn: 2	22,632 (142.24)	251,738 (132.40)	44 (129.97)
Cg: 1	2,208 (149.66)	250,654 (107.15)	12 (32.10)
Cc: 3	34,856 (205.48)	320,544 (203.06)	213 (932.97)
Cn: 3	19,672 (112.92)	201,874 (104.25)	32 (93.78)
Cg: 3	2,131 (129.61)	214,146 (89.96)	10 (27.05)
Cc: 4	21,531 (180.09)	246,615 (156.21)	51 (140.09)
Cn: 4	18,736 (108.14)	194,417 (100.35)	29 (86.44)
Cg: 4	1,669 (101.45)	166,216 (70.39)	8 (22.01)
Cc: 5	20,885 (155.91)	238,793 (144.07)	30 (89.65)
Cn: 5	18,354 (104.93)	189,479 (97.58)	29 (87.44)

Cg: 5	1,799 (97.84)	158,256 (66.13)	15 (45.93)
Cc: 7	18,023 (127.05)	193,038 (114.34)	31 (87.39)
Cn: 7	15,910 (80.01)	147,303 (73.86)	20 (61.99)
Cg: 6	1,645 (85.93)	137,784 (58.90)	5 (16.19)
Cc: 6	16,665 (135.95)	192,167 (119.76)	56 (158.88)
Cn: 6	15,714 (102.89)	166,981 (87.99)	28 (84.54)
Cg: 7	1,716 (76.8)	113,913 (49.42)	14 (36.36)
Cc: 10	14,726 (85.21)	140,387 (73.36)	14 (40.68)
Cn: 8	15,224 (80.56)	125,292 (62.22)	15 (41.63)
Cg: 8	1,291 (70.47)	111,304 (46.46)	13 (31.77)
Cc: 8	14,450 (105.92)	162,234 (93.20)	27 (79.73)
Cn: 9	13,904 (69.67)	85,170 (42.94)	15 (46.37)
Cg: 9	1,424 (67.65)	104,771 (43.83)	9 (22.56)
Cc: 12	11,293 (67.98)	109,530 (56.71)	12 (32.79)
Cn: 10	11,824 (55.65)	140,140 (70.45)	14 (43.92)
Cg: 10	905 (45.32)	67,686 (28.04)	11 (29.99)
Cc: 13	6,741 (48.56)	72,574 (37.91)	4 (8.48)
Cn: 13	7,463 (44.075)	70,424 (32.34)	10 (29.76)
Cg: 11	727 (40.68)	54,183 (26.72)	3 (9.35)

Cc: 11	8,998 (76.39)	111,643 (65.12)	12 (33.22)
Cn: 12	8,949 (52.86)	100,271 (47.09)	17 (57.18)
Cg: 12	628 (34.46)	41,986 (20.14)	10 (27.67)
Cc: 9 (X chromosome)	14,276 (97.82)	131,038 (93.20)	30 (75.68)
Cn: 11 (X chromosome)	11,198 (44.26)	85,119 (44.32)	4 (11.54)
Cg: 13 (X chromosome)	2,217 (42.29)	58,444 (24.89)	8 (18.46)

Supplementary Table 2. *Ccb* HGT annotations for Chromosomes 1 (C1) and 3 (C3). Bolded rows denote genes with significant branch-site likelihood ratio tests for positive selection.

Gene ID	Protein annotation	C1 fragments	C3 fragments	C1 coordinates	C3 coordinates
**WP_395479791.1**	**ATP-dependent chaperone ClpB**	**1**	**1**	**134011286-134013155**	**136812448-136814956**
WP_395479792.1	transcription antiterminator/RNA stability regulator CspE	1	1	134205796-134206005	136615288-136615488
**WP_395480257.1**	**ATP-dependent zinc metalloprotease FtsH**	**1**	**1**	**134041437-134043351**	**136841934-136843849**
WP_395480164.1	type I glyceraldehyde-3-phosphate dehydrogenase	1	1	134532379-134533350	137265139-137266109
WP_395479678.1	chaperonin GroEL	1	2	134156614-134158268	137177374-137179012; 137221463-137222914
**WP_395479659.1**	**isoleucine--tRNA ligase**	**1**	**1**	**134001521-134004334**	**136804177-136807004**
WP_395479776.1	translation initiation factor IF-2	1	1	134033583-134036180	136836113-136837077
**WP_395479794.1**	**leucine--tRNA ligase**	**1**	**1**	**134201489-134204062**	**136617191-136619771**
WP_395479804.1	lipoyl synthase	1	2	134191493-134192395	136514630-136515532; 136628929-136629831
WP_395479660.1	signal peptidase II	1	1	134004334-134004799	136807004-136807469
WP_395479801.1	peptidoglycan DD-transpeptidase MrdA	1	1	134196682-[\n]134198534	136622725-136624567
WP_395480236.1	NADH-quinone oxidoreductase subunit NuoG	1	1	134515014-134517720	137247472-137250161
WP_395479860.1	two-component system response regulator PhoP	1	2	134246882-134247452	136523760-136524425; 136572776-136573441
WP_395479690.1	pyruvate kinase	1	2	134136677-134138116	137198008-137199435; 137201040-137202467

**WP_395479665.1**	**transcription termination factor Rho**	**1**	**1**	**134220083-134221345**	**136597410-136598672**
WP_395479658.1	bifunctional riboflavin kinase/FAD synthetase	1	1	134000742-134001468	136803206-136804124
WP_395480165.1	signal peptide peptidase SppA	1	1	134529506-134531177	137262271-137263938
WP_395480266.1	translational GTPase TypA	1	1	134283263-134285056	137224800-137226590
WP_395479685.1	2-octaprenyl-6-methoxyphenyl hydroxylase	1	2	134144156-134145323	137190796-137191961; 137208508-137209676
WP_395479688.1	glucose-6-phosphate dehydrogenase	1	2	134139312-134140447	137195331-137196774; 137203701-137205144
**WP_395479664.1**	**proline--tRNA ligase**	**1**	**1**	**134214784-134216504**	**136602250-136603991**
WP_395479677.1	co-chaperone GroES	1	1	134158315-134158608	137177034-137177327
**WP_395479781.1**	**DEAD/DEAH family ATP-dependent RNA helicase**	**1**	**1**	**134027062-134028334**	**136827634-136829330**
WP_395479784.1	3-deoxy-7-phosphoheptulonate synthase	1	1	134017859-134018869	136819167-136819807
WP_395479763.1	acetyl-CoA carboxylase biotin carboxylase subunit		1		136849019-136849673
WP_395480224.1	pyruvate dehydrogenase (acetyl-transferring), homodimeric type		1		136703291-136705035
WP_395479942.1	arginine--tRNA ligase	1		134321652-134323387	
WP_395480018.1	shikimate kinase AroK		1		136692605-136693119
WP_395479814.1	archaetidylserine decarboxylase	1		134180320-134181189	
WP_395480232.1	cytidine deaminase		1		137318499-137319314
WP_395479875.1	ATP-dependent Clp protease ATP-binding subunit ClpX	1		134308438-134309712	
WP_395480167.1	cardiolipin synthase		1		137256671-137257852

ACICP0_RS01840	(d)CMP kinase	1		134404298-134404935	
WP_395480219.1	magnesium/cobalt transporter CorA	1		134434714-134435659	
WP_395479880.1	ubiquinol oxidase subunit II	1		134297338-134298157	
WP_395479881.1	cytochrome o ubiquinol oxidase subunit I	1		134295226-134297220	
WP_395480211.1	ribosome biogenesis GTPase Der	1		134448976-134449738	
WP_395479918.1	molecular chaperone DnaK	2		134253849-134255290; 271083270-271083644	
WP_395480154.1	dipeptide/tripeptide permease DtpA		1		137329703-137331149
WP_395479887.1	1-deoxy-D-xylulose-5-phosphate synthase		2		137234124-137235917; 137227594-137229398
WP_395479830.1	phosphopyruvate hydratase		1		137155687-137156966
WP_395479636.1	3-hydroxyacyl-ACP dehydratase FabZ	1		134371636-134372091	
ACICP0_RS03070	cell division protein FtsW		1		136560104-136561200
WP_395479870.1	cell division protein FtsZ		2		136554166-136554764; 136638432-136639042
WP_395480233.1	class II fumarate hydratase		1		137338087-137339433
WP_395479771.1	phosphoglucosamine mutase	1		134039140-134040456	
WP_395479890.1	glutamine--fructose-6-phosphate transaminase (isomerizing)	1		134272349-134274188	
WP_395479984.1	glycine--tRNA ligase subunit alpha	1		134344071-134344975	
WP_395479985.1	glycine--tRNA ligase subunit beta	1		134341993-134344053	
WP_395479873.1	xanthine phosphoribosyltransferase	1		134314626-134315087	
WP_395479600.1	glutamate--cysteine ligase	1		134416623-134417111	

WP_395479732.1	protease modulator HflC	1		134067855-134068853	
WP_395480163.1	histone-like nucleoid-structuring protein H-NS		1		137267742-137268142
WP_395479808.1	molecular chaperone HtpG	2		134185083-134186956; 134189638-134189741	
WP_395479616.1	integration host factor subunit beta	1		134402094-134402377	
WP_395480226.1	translation initiation factor IF-3		1		136679537-136680084
WP_395479630.1	1-deoxy-D-xylulose-5-phosphate reductoisomerase	1		134379728-134380923	
WP_395480141.1	4-(cytidine 5'-diphospho)-2-C-methyl-D-erythritol kinase		1		137303168-137304015
WP_395480214.1	flavodoxin-dependent (E)-4-hydroxy-3-methylbut-2-enyl-diphosphate synthase	1		134443396-134444501	
WP_395480245.1	(2E,6E)-farnesyl-diphosphate-specific ditrans,polycis-undecaprenyl-diphosphate synthase	1		134378806-134379533	
WP_395479909.1	3-deoxy-8-phosphooctulonate synthase		1		136669688-136670530
WP_395479874.1	endopeptidase La	1		134309917-134312242	
WP_395480225.1	dihydrolipoyl dehydrogenase		1		136700382-136701816
WP_395479638.1	lipid-A-disaccharide synthase	1		134369696-134370818	
WP_395479871.1	UDP-3-O-acyl-N-acetylglucosamine deacetylase		2		136553153-136554051; 136639157-136640055
WP_395479722.1	cystathionine beta-lyase	1		134082788-134083960	
WP_395480008.1	methionine adenosyltransferase		1		136709752-136710876

WP_395480134.1	septum site-determining protein MinD		1		137281853-137282665
WP_395479714.1	membrane-bound lytic murein transglycosylase MltC	1		134093035-134094096	
WP_395479858.1	tRNA 2-thiouridine(34) synthase MnmA	1		134243406-134244529	
WP_395479759.1	rod shape-determining protein MreC	1		134063160-134063981	
WP_395479695.1	UDP-N-acetylglucosamine 1-carboxyvinyltransferase	1		134128210-134129446	
WP_395479868.1	UDP-N-acetylmuramate--L-alanine ligase		2		136557379-136558377; 136634831-136635829
WP_395480261.1	UDP-N-acetylmuramoyl-L-alanyl-D-glutamate--2,6-diaminopimelate ligase		1		136565065-136566435
WP_395479717.1	A/G-specific adenine glycosylase	1		134091633-134092462	
WP_395480176.1	NADH-quinone oxidoreductase subunit A	1		134510056-134510324	
WP_395479774.1	transcription termination factor NusA		1		136837121-136838586
WP_395479623.1	2-oxoglutarate dehydrogenase complex dihydrolipoyllysine-residue succinyltransferase	1		134386914-134388104	
WP_395479812.1	oligoribonuclease	1		134182206-134182742	
WP_395479906.1	leucyl aminopeptidase		1		136677432-136678358
WP_395480016.1	glucose-6-phosphate isomerase		1		136695734-136697346
WP_395479617.1	6-phosphogluconolactonase	1		134397434-134398412	

WP_395479861.1	two-component system sensor histidine kinase PhoQ		2		136571316-136572764; 136524437-136525882
WP_395480240.1	metalloprotease PmbA	1		134450640-134451977	
WP_395479656.1	nicotinate phosphoribosyltransferase		1		136798399-136799600
WP_395480143.1	aminoacyl-tRNA hydrolase		1		137305868-137306432
WP_395479832.1	glutamine hydrolyzing CTP synthase		1		137156994-137158623
WP_395479599.1	recombinase RecA	1		134421914-134422906	
WP_395480179.1	exodeoxyribonuclease V subunit beta	1		134497423-134499278	
WP_395480123.1	lipopolysaccharide heptosyltransferase RfaC		1		137375170-137376093
WP_395480002.1	50S ribosomal protein L1		1		136722923-136723607
WP_395480001.1	50S ribosomal protein L10		1		136723950-136724202
WP_395480000.1	50S ribosomal protein L7/L12		1		136724452-136724824
WP_395480190.1	50S ribosomal protein L16	1		134484908-134485310	
WP_395479999.1	DNA-directed RNA polymerase subunit beta		2		136725154-136727774; 136727782-136729112
WP_395479998.1	DNA-directed RNA polymerase subunit beta'		1		136729205-136731079
WP_395479940.1	RNA pyrophosphohydrolase		1		136543213-136543701
WP_395479615.1	30S ribosomal protein S1	1		134402458-134404144	
WP_395480200.1	30S ribosomal protein S5	1		134462941-134463428	
WP_395480197.1	30S ribosomal protein S8	1		134482306-134482698	
WP_395480206.1	30S ribosomal protein S11	1		134459877-134460261	

WP_395479963.1	16S rRNA (guanine(966)-N(2))-methyltransferase RsmD		1		136748028-136748551
WP_395479620.1	succinate dehydrogenase flavoprotein subunit	1		134391905-134393672	
ACICP0_RS03110	preprotein translocase subunit SecA		2		136549490-136551997; 136641211-136643722
WP_395480203.1	preprotein translocase subunit SecY	1		134460961-134462270	
WP_395479634.1	molecular chaperone Skp	1		134373247-134373744	
WP_395479959.1	transcriptional regulator SlyA	1		134330553-134330980	
WP_395479724.1	spermidine N1-acetyltransferase	1		134077399-134077937	
WP_395480221.1	Fe-S cluster assembly protein SufB		1		136751499-136752985
WP_395479640.1	tRNA adenosine(34) deaminase TadA	1		134364325-134364642	
WP_395479856.1	thiamine/thiamine pyrophosphate ABC transporter permease	1		134238793-134240350	
WP_395480026.1	threonine--tRNA ligase		2		136680660-136681890; 136680090-136680633
WP_395480015.1	metalloprotease TldD		1		136698690-136700122
WP_395480138.1	type I DNA topoisomerase		1		137289396-137291967
WP_395479650.1	elongation factor Tu		1		136771958-136773080
WP_395479935.1	4-hydroxy-3-polyprenylbenzoate decarboxylase		1		136533483-136534953
WP_395479718.1	uracil phosphoribosyltransferase	1		134089785-134090410	
WP_395480122.1	lipid IV(A) 3-deoxy-D-manno-octulosonic acid transferase		1		137384137-137385348
WP_395480259.1	tol-pal system protein YbgF		1		137150885-137151306

WP_395479922.1	membrane protein insertase YidC	1		134248610-134250247	
WP_395479923.1	membrane protein insertion efficiency factor YidD	1		134248360-134248597	
WP_395480242.1	fructose-1-phosphate/6-phosphogluconate phosphatase	1		134417204-134417763	
WP_395479621.1	succinate dehydrogenase iron-sulfur subunit	1		134391172-134391884	
WP_395479709.1	MBL fold metallo-hydrolase	1		134115558-134116146	
WP_395479723.1	single-stranded DNA-binding protein	1		134079177-134079696	
WP_395479760.1	rod shape-determining protein	1		134062011-134063050	
WP_395479846.1	metal ABC transporter permease	1		134178515-134179307	
WP_395480022.1	YchF/TatD family DNA exonuclease		1		136687410-136688080
WP_395480142.1	ribose-phosphate pyrophosphokinase		1		137304282-137305231
WP_395480158.1	2-hydroxyacid dehydrogenase		1		137342741-137343736
WP_395480207.1	DNA-directed RNA polymerase subunit alpha	1		134458242-134459201	
ACICP0_RS03380	DNA polymerase III subunit chi		1		136676868-136677295

Supplementary Table 3. Pairwise Jukes-Cantor genetic distances estimated across four *Ccb*-derived homologous regions: the Chromosome 1 region, the upstream Chromosome 3 region (A), the downstream Chromosome 3 region (B), the extrachromosomal contig (JAKZMK010000236.1), and the *Ccb* reference genome.

	**Chromosome 1**	**Chromosome 3A**	**Chromosome 3B**	***Ccb* genome**	**JAKZMK010000236.1**
**Chromosome 1**	-	0.0277	0.0560	0.222	0.00162
**Chromosome 3A**		-	0.110	0.217	0.0143
**Chromosome 3B**			-	0.229	0.0253
***Ccb* genome**				-	0.214

Supplementary Table 4. Genome-wide repeat family composition in *C. caryae.* Counts and cumulative sizes are shown for each TE family in regions classified as old or young based on the Kimura *D* thresholds described in *Methods*.

TE family	Counts of old TEs	Counts of young TEs	Cumulative size of old TEs (bp)	Cumulative size of young TEs (bp)	Proportion of young TE counts (col2/[col1+2)	Proportion of young TE size col4/(col3+4)
Unknown	973,281	297,975	368,832,962	236,460,405	0.234	0.391
DNA/Maverick	141,401	45,094	103,679,464	113,497,558	0.242	0.523
LTR/Gypsy	102,346	43,780	73,303,501	102,943,396	0.300	0.584
DNA/TcMar-Tc1	110,048	43,108	34,797,779	63,913,645	0.281	0.647
LINE/R1-LOA	99,143	26,974	53,294,578	32,231,503	0.214	0.377
LINE/Dong-R4	28,058	14,077	20,127,356	31,967,322	0.334	0.614
LINE/L2	49,374	14,496	18,841,058	21,892,504	0.227	0.537
DNA/hAT-Tip100	38,478	16,050	14,290,869	20,424,795	0.294	0.588
DNA/P	26,179	10,282	7,917,624	11,940,737	0.282	0.601

LINE/I	8,261	3,554	5,440,727	10,762,498	0.301	0.664
LINE/Penelope	25,131	11,384	7,916,460	10,210,735	0.312	0.563
LINE/I-Jockey	60,745	6,476	31,856,392	8,741,410	0.096	0.215
LTR/Pao	14,986	3,833	7,358,761	8,151,393	0.204	0.526
LINE/CR1	18,860	6,495	8,024,478	7,571,152	0.256	0.485
LINE/RTE-BovB	49,019	5,938	23,477,268	6,994,769	0.108	0.230
DNA/TcMar-m44	10,357	6,712	3,106,917	6,675,792	0.393	0.682
DNA/Academ-1	31,907	6,620	11,319,228	6,241,069	0.172	0.355
DNA/Crypton-I	12,571	6,397	3,205,642	5,933,605	0.337	0.649
LTR/Copia	7,146	2,796	3,639,347	5,702,881	0.281	0.610
DNA/hAT-Ac	13,572	5,356	3,225,804	5,427,226	0.283	0.627
DNA/CMC-Chapaev-3	7,419	3,830	2,170,790	3,778,964	0.340	0.635
DNA/hAT-Blackjack	4,003	2,560	908,160	3,623,481	0.390	0.800
DNA/CMC-Transib	13,861	2,256	3,856,709	3,429,823	0.140	0.471
DNA/hAT-Tag1	3,326	2,381	902,899	3,118,653	0.417	0.775
LTR/DIRS	2,705	1,330	1,414,810	3,112,689	0.330	0.688
LINE	8,379	3,616	2,482,089	3,042,868	0.301	0.551
DNA	13,209	2,767	2,825,787	2,912,762	0.173	0.508
DNA/MULE-MuDR	5,001	2,060	1,316,320	2,662,063	0.292	0.669
RC/Helitron	3,222	2,012	682,616	1,805,910	0.384	0.726
LINE/R1	9,841	992	4,927,087	1,596,994	0.092	0.245
DNA/PiggyBac	10,682	1,934	3,003,633	1,580,541	0.153	0.345
DNA/Crypton-V	9,767	812	2,116,807	1,523,719	0.077	0.419
DNA/PIF-ISL2EU	4,853	924	1,203,164	1,203,482	0.160	0.500
DNA/TcMar-Mariner	29,155	1,395	6,204,198	1,155,689	0.046	0.157
DNA/Crypton-A	3,202	1,898	797,748	1,118,009	0.372	0.584

DNA/hAT-hATm	3,311	770	1,113,751	1,097,921	0.189	0.496
DNA/PIF-Harbinger	11,632	1,137	3,247,565	1,028,550	0.089	0.241
DNA/TcMar-Fot1	7,340	588	2,977,426	890,098	0.074	0.230
DNA/Sola-1	2,029	422	598,101	637,317	0.172	0.516
DNA/MULE-NOF	18	162	8,959	577,712	0.900	0.985
DNA/hAT-Charlie	7,059	1,223	1,504,390	569,520	0.148	0.275
DNA/Merlin	3,943	761	2,114,116	543,556	0.162	0.205
DNA/CMC-EnSpm	1,662	216	208,814	542,776	0.115	0.722
DNA/TcMar-Pogo	3,229	542	905,917	463,905	0.144	0.339
LINE/RTE	2,423	576	426,344	427,680	0.192	0.501
LINE/RTE-RTE	5,417	333	3,131,003	416,433	0.058	0.117
DNA/Sola-2	71	189	42,950	412,220	0.727	0.906
DNA/Ginger-2	1,731	494	445,313	342,540	0.222	0.435
DNA/TcMar-Tigger	4,146	263	1,169,562	311,588	0.060	0.210
SINE/5S-Deu-L2	4,230	1,451	891,936	299,471	0.255	0.251
DNA/hAT-hAT19	314	300	96,011	289,610	0.489	0.751
LINE/CRE	956	154	372,149	154,666	0.139	0.294
DNA/hAT-hobo	258	324	76,252	135,410	0.557	0.640
DNA/Kolobok-E	746	187	81,816	109,145	0.200	0.572
DNA/TcMar-ISRm11	74	54	38,270	38,360	0.422	0.501
DNA/P-Fungi	308	28	60,178	28,566	0.083	0.322
LINE/R2	135	3	81,543	7,827	0.022	0.088
DNA/hAT-hATx	288	10	124,293	1,467	0.034	0.012

Supplementary Table 5. Genome-wide repeat family composition in *C. nanulus.* Counts and cumulative sizes are shown for each TE family in regions classified as old or young based on the Kimura *D* thresholds described in *Methods*.

TE family	Counts of old TEs	Counts of young TEs	Cumulative size of old TEs (bp)	Cumulative size of young TEs (bp)	Proportion of young TE counts (col2/[col1+2)	Proportion of young TE size col4/(col3+4)
Unknown	860198	260337	302511088	208,232,464	0.232	0.408
DNA/TcMar-Tc1	80666	26423	23127520	31989071	0.247	0.58
LTR/Gypsy	44402	12243	24497727	31863409	0.216	0.565
DNA/Maverick	33164	7098	21049344	22138849	0.176	0.513
LINE/R1-LOA	76653	17104	40687808	17760158	0.182	0.304
LINE/L2	32337	11171	13849309	17604366	0.257	0.56
LINE/RTE-BovB	61032	9083	33051603	13526336	0.13	0.29
DNA/hAT-Tip100	36628	14475	12368538	13298514	0.283	0.518
LTR/Pao	20461	5576	9273342	10939533	0.214	0.541
DNA/Academ-1	33921	7455	13040093	8988532	0.18	0.408
LINE/Penelope	20346	8566	6447707	6848810	0.296	0.515
LINE/Dong-R4	3271	2460	2265263	5847783	0.429	0.721
DNA/P	19102	5079	4814945	5377613	0.21	0.528
LINE/I-Jockey	42426	3664	25562598	4947234	0.079	0.162
LINE/CR1	10254	4057	3829837	4725926	0.283	0.552
DNA/hAT-Ac	12018	5323	2227163	4013086	0.307	0.643
LINE/RTE-RTE	10414	2644	5790173	3884001	0.202	0.401
DNA/CMC-Transib	9056	3801	3024239	3237049	0.296	0.517
DNA/TcMar-m44	6211	3207	1788629	2905524	0.341	0.619
LTR/Unknown	10003	2602	5278446	2865878	0.206	0.352
LINE/I	9375	1935	5217129	2855489	0.171	0.354
DNA/hAT-Tag1	4475	2519	1033830	2783305	0.36	0.729
RC/Helitron	4790	2064	1120022	2591361	0.301	0.698
LTR/Copia	2749	1718	1081923	2515427	0.385	0.699
DNA/Crypton-I	11594	2313	2666221	2007677	0.166	0.43
DNA/hAT-Blackjack	6069	2737	1111437	1665107	0.311	0.6
DNA	9460	1606	1740290	1325904	0.145	0.432

LINE/R1	10262	1130	5448623	1137964	0.099	0.173
DNA/PIF-Harbinger	9246	1267	2319490	1017153	0.121	0.305
DNA/PiggyBac	5812	819	1471122	983247	0.124	0.401
DNA/MULE-MuDR	6013	956	1522523	831278	0.137	0.353
DNA/CMC-Chapaev-3	5359	975	1555815	796680	0.154	0.339
DNA/TcMar-Mariner	36584	1550	6938399	610651	0.041	0.081
DNA/Sola-1	3330	513	769268	604814	0.133	0.44
LTR/DIRS	2998	388	1042305	551993	0.115	0.346
DNA/PIF-ISL2EU	3805	576	818300	544116	0.131	0.399
DNA/hAT-hATm	3146	670	940214	540416	0.176	0.365
LINE	1589	446	273277	491207	0.219	0.643
DNA/TcMar-Fot1	6482	360	2362612	476519	0.053	0.168
DNA/Crypton-V	3456	591	690060	423050	0.146	0.38
DNA/hAT-Charlie	2149	368	563546	411466	0.146	0.422
DNA/TcMar-Pogo	3297	404	866928	342591	0.109	0.283
LTR	2358	228	699280	321452	0.088	0.315
DNA/Sola-2	245	119	86740	250419	0.327	0.743
DNA/Crypton-A	1056	288	334374	249879	0.214	0.428
LINE/RTE	375	138	374971	226368	0.269	0.376
DNA/hAT-hATx	1170	291	359224	217521	0.199	0.377
DNA/P-Fungi	213	75	99828	207693	0.26	0.675
DNA/TcMar-ISRm11	121	188	72152	193501	0.608	0.728
DNA/Ginger-2	1881	290	587495	183350	0.134	0.238
DNA/Kolobok-E	363	94	86261	179904	0.206	0.676
DNA/TcMar-Tigger	4560	279	1021166	151625	0.058	0.129
LINE/CRE	830	110	356181	139651	0.117	0.282
DNA/MULE-NOF	33	141	5363	134550	0.81	0.962
DNA/CMC-Chapaev	234	55	85393	125985	0.19	0.596

DNA/hAT-hAT5	129	73	60617	118998	0.361	0.663
DNA/PIF-Spy	198	40	111411	107056	0.168	0.49
SINE/5S-Deu-L2	2359	568	680975	85843	0.194	0.112
DNA/hAT-hAT19	826	201	138464	85611	0.196	0.382
DNA/CMC-EnSpm	233	26	65423	54667	0.1	0.455
DNA/hAT-hobo	177	140	46377	52914	0.442	0.533
DNA/Zator	72	83	6888	43826	0.535	0.864
DNA/Merlin	77	44	21021	16412	0.364	0.438
LINE/R2	309	7	165209	125	0.022	0.001
LINE/CR1-Zenon	233	0	25961	0	0.0	0.0

Supplementary Table 6. Genome-wide repeat family composition in *C. glandium.* Counts and cumulative sizes are shown for each TE family in regions classified as old or young based on the Kimura *D* thresholds described in *Methods*.

TE family	Counts of old TEs	Counts of young TEs	Cumulative size of old TEs (bp)	Cumulative size of young TEs (bp)	Proportion of young TE counts (col2/[col1+2)	Proportion of young TE size col4/(col3+4)
Unknown	743872	250051	191785851	151502164	0.252	0.441
LTR/Gypsy	45924	31407	22461590	55787330	0.406	0.713
DNA/TcMar-Tc1	116649	33615	32025015	29768072	0.224	0.482
LTR/Pao	22403	11226	10771189	15084764	0.334	0.583
LINE/R1-LOA	44497	10659	13030921	14172632	0.193	0.521
LINE/L2	36957	8537	15664468	10065055	0.188	0.391
LINE/Penelope	17216	10663	4852781	8059290	0.382	0.624
DNA/Academ-1	25865	7488	6151110	7379495	0.225	0.545
DNA/TcMar-m44	9500	7420	2301472	6814775	0.439	0.748
DNA/Maverick	8958	2322	3764669	6665791	0.206	0.639
LINE/CR1	12905	4637	5246366	5881359	0.264	0.529
DNA/hAT-Tip100	17266	8160	3691537	5501090	0.321	0.598
LINE/Dong-R4	1249	2491	668075	4762971	0.666	0.877

DNA/Crypton-I	10389	4863	2821320	4312149	0.319	0.604
LINE/I	8536	2592	4651643	4254887	0.233	0.478
LINE/RTE-BovB	20074	2433	7066956	3667257	0.108	0.342
DNA/P	10564	4183	2418784	3373657	0.284	0.582
LTR/Unknown	5695	4349	2208040	3140765	0.433	0.587
LTR/Copia	4420	1994	1671567	3035052	0.311	0.645
LINE/RTE-RTE	2496	1124	1636015	2061595	0.310	0.558
DNA/hAT-hATm	1592	1491	587392	1922984	0.484	0.766
DNA	5455	2678	1030447	1907902	0.329	0.649
LINE/I-Jockey	12222	1741	4421364	1742375	0.125	0.283
DNA/Sola-1	5058	2447	1103116	1732967	0.326	0.611
RC/Helitron	1903	1892	339178	1572617	0.499	0.823
DNA/CMC-Transib	6354	1215	1482578	1239609	0.161	0.455
DNA/CMC-EnSpm	2192	1470	617442	1041833	0.401	0.628
DNA/CMC-Chapaev-3	4327	909	1306944	1011536	0.174	0.436
DNA/PIF-Harbinger	4790	1150	1122901	965153	0.194	0.462
DNA/PIF-ISL2EU	3036	1470	386093	951258	0.326	0.711
DNA/hAT-Ac	2838	1336	502184	797476	0.320	0.614
DNA/PiggyBac	7941	1169	2657553	755198	0.128	0.221
DNA/TcMar-Fot1	3916	725	1435248	717265	0.156	0.333
LINE	2200	1061	725017	629303	0.325	0.465
DNA/Crypton-V	4457	693	634542	584270	0.135	0.479
DNA/hAT-Charlie	625	394	121299	505776	0.387	0.807
DNA/TcMar-Tigger	758	400	125732	327220	0.345	0.722
DNA/MULE-MuDR	1578	486	320271	316670	0.235	0.497
DNA/Crypton-A	843	525	287581	290684	0.384	0.503
DNA/TcMar-Mariner	8400	410	1328198	290543	0.047	0.179
LTR/DIRS	1439	153	590006	234679	0.096	0.285

DNA/Sola-2	253	267	25990	231561	0.513	0.899
DNA/PIF-Spy	173	160	41185	209842	0.480	0.836
DNA/hAT-Tag1	1467	378	341837	196726	0.205	0.365
DNA/Ginger-1	50	332	3984	192706	0.869	0.980
DNA/hAT-Blackjack	6555	436	1577829	168779	0.062	0.097
DNA/hAT-hAT19	1676	191	371910	166050	0.102	0.309
LINE/R1	4644	119	1614508	139193	0.025	0.079
DNA/Kolobok-E	1870	346	435300	128692	0.156	0.228
DNA/hAT-hATx	702	173	108731	104353	0.198	0.490
DNA/TcMar-Pogo	475	137	133787	101343	0.224	0.431
DNA/TcMar-ISRm11	468	94	158617	98868	0.167	0.384
DNA/CMC-Chapaev	158	76	27970	53379	0.325	0.656
DNA/Ginger-2	406	15	114491	23142	0.036	0.168
SINE/tRNA-V	188	75	37941	15488	0.285	0.290
LTR	7	4	2604	3007	0.364	0.536
LINE/R2	145	1	76491	2404	0.007	0.030
LINE/RTE	947	3	201869	93	0.003	0.000
LINE/CRE	245	1	46362	28	0.004	0.001
LINE/RTE-X	103	0	47729	0	0.000	0.000
